# Large language models in rheumatology: applications, methodological quality and clinical translation – a systematic review

**DOI:** 10.1136/rmdopen-2026-007538

**Published:** 2026-10-28

**Authors:** Phillip Kremer, Isabell Haase, Zhivana Boyadzhieva, Martin Krusche

**Affiliations:** 1 Division of Rheumatology and Systemic Inflammatory Diseases, III. Department of Medicine, University Medical Center Hamburg-Eppendorf, Hamburg, Germany; 2 Department of Rheumatology and Clinical Immunology, Charité – University Medical Center Berlin, Berlin, Germany

**Keywords:** Qualitative research, Autoimmune Diseases, Internet

## Abstract

**Objectives:**

To map applications of large language models (LLMs) in rheumatology and evaluate the methodological quality and clinical translation of the evidence.

**Methods:**

MEDLINE, Embase and Web of Science were searched from inception to 13 July 2026 for primary studies of LLMs in rheumatology. Two reviewers independently screened records and analysed data. Each study was assigned to one application domain and to the highest level of a review-derived translational maturity framework. Risk of bias was assessed with the Alberta Quality Assessment Tool: Risk of Bias (AQAT:RoB; worst-item rule), where human evaluation of free-text answers was reported; findings were synthesised narratively.

**Results:**

93 studies were included, 79 (84.9%) from 2025 or 2026. Patient education (39, 41.9%) and clinical decision support (38, 40.9%) dominated. Accuracy in clinical decision support was strongly task-dependent, with medians of 90.9% for structured diagnostic tasks and 40.0% for open-ended differential diagnosis (best-performing model per study). Patient-facing output had a median Flesch-Kincaid grade of 12.2; none of the 13 readability studies met the recommended sixth-grade target. 22 studies (23.7%) used real-world clinical data, mostly retrospectively; only four (4.3%) evaluated an LLM prospectively in a clinical workflow. Prompts were fully reported in 59.1% of studies, and answer stability was tested in 19.4%. Among the 68 studies amenable to AQAT:RoB assessment, five (7.4%) were at low risk of bias.

**Conclusions:**

LLM research in rheumatology is expanding rapidly but remains dominated by prompt-response benchmarks with surrogate outcomes, incomplete reporting and, among AQAT:RoB-assessable studies, predominantly unclear or high risk of bias. Whether LLMs improve rheumatology care remains unresolved; prospective evaluations with patient-relevant endpoints are required.

**WHAT IS ALREADY KNOWN ON THIS TOPIC**

- Large language models (LLMs) are increasingly evaluated across clinical, educational and patient-facing tasks in rheumatology, but the evidence is dispersed, and existing rheumatology-specific syntheses have addressed model openness and reporting practices rather than a study-level assessment of methodological quality and clinical translation.
- Cross-specialty reviews have shown that LLM evaluations rarely use real patient care data and rely predominantly on examination-style benchmarks.

**WHAT THIS STUDY ADDS**

- Across 93 studies, the evidence remains focused on patient education and clinical decision support, with most studies relying on prompt–response benchmarking and surrogate outcomes. Translational maturity is beginning to improve: 22 studies (23.7%) used real-world clinical data, in most cases retrospectively; only six studies (6.5%) reached prospective, workflow-embedded evaluation: four non-randomised prospective evaluations (4.3%) and two randomised trials (2.2%).
- However, evidence on patient-relevant outcomes remained very limited. Disease activity was assessed in three studies (3.2%), exclusively as a model prediction or scoring target, while validated patient-reported outcomes were measured in only two studies (2.2%), both without a control group. No study demonstrated improvement in downstream clinical outcomes attributable to LLM-supported care.
- Reporting and risk of bias remain the central weakness: prompts were fully reported in 59.1%, answer stability was tested in 19.4%, and only 7.4% of the Alberta Quality Assessment Tool: Risk of Bias (AQAT:RoB)-assessable studies were at low risk of bias, while the tool was not applicable in 26.9% of the studies.

**HOW THIS STUDY MIGHT AFFECT RESEARCH, PRACTICE OR POLICY**

- Future evaluations should move from retrospective benchmarking to prospective, workflow-embedded designs with patient-relevant endpoints and systematic safety assessment.
- Complete reporting of models, versions, prompts, access dates and inference settings, following design-appropriate reporting guidance for artificial intelligence, is a prerequisite for reproducibility and for any clinical adoption decision.

## Introduction

 Artificial intelligence (AI) is being adopted across healthcare at an accelerating pace, with recent momentum driven by the emergence of large language models (LLMs) deployed in conversational systems such as ChatGPT (OpenAI).^1 2^ Unlike predictive AI, which primarily estimates outcomes or classifies inputs, generative AI creates new content, including text, images and audio.^3^ Among generative approaches, LLMs are designed to produce structured, coherent text in response to prompts, enabling a wide range of applications in clinical and health system workflows.^4^

In rheumatology, early studies suggest that LLMs may support diagnostic reasoning, treatment planning and patient communication.^5^ Recent evaluations indicate that LLMs may achieve diagnostic accuracy above chance when presented with case summaries,^6^ outperform classic diagnostic decision-support systems and identify rare rheumatic diseases with high accuracy.^7 8^ Moreover, LLM-generated treatment plans can be clinically plausible, yet are overall less preferred than specialist decisions and require careful governance for higher-stakes or complex scenarios.^9^ Patient-facing rheumatology use cases are also emerging, including assessments of responses to frequently asked questions in connective tissue diseases and AI-generated information for psoriatic arthritis.^10 11^

However, the rapid evolution of LLM capabilities, including substantial performance differences between model versions and frequent updates, creates challenges for evidence synthesis and temporal validity that warrant focused attention.^12 13^ Across healthcare, current LLM evaluations are methodologically heterogeneous and predominantly vignette- or examination-based, with only approximately 5% using real patient care data.^13^

To date, LLM-specific evidence in rheumatology remains dispersed across tasks, settings and evaluation paradigms, limiting the conclusions that can be drawn for clinical practice.^14^ A recent scoping review has mapped model openness and reporting practices in rheumatology-centred LLM research,^15^ but a rheumatology-specific synthesis at the level of individual reported outcomes, combined with a formal risk-of-bias assessment, is still needed to determine what the available evidence actually supports.

In this systematic review, we map the applications of LLMs in rheumatology and assess the methodological quality, reproducibility, safety evaluation and translational maturity of the available evidence.

## Methods

### Protocol and registration

This systematic review was conducted in accordance with the Preferred Reporting Items for Systematic Reviews and Meta-Analyses (PRISMA) statement.^16^ The review protocol was prospectively registered in PROSPERO on 20 May 2026 (registration number CRD420261281893). The objective was to systematically identify and synthesise the current evidence on the use of LLMs in rheumatology, including their performance, safety, validity and clinical or educational utility.

### Eligibility criteria

Studies were eligible if they investigated LLMs in a rheumatology-related context. Eligible conditions included all rheumatic and musculoskeletal diseases, including but not limited to rheumatoid arthritis, psoriatic arthritis, axial spondyloarthritis, systemic lupus erythematosus, Sjögren’s disease, systemic sclerosis, idiopathic inflammatory myopathies, vasculitides, osteoarthritis and autoinflammatory diseases. No restrictions were placed on the setting or context of the evaluation.

Comparators, where applicable, included rheumatologists or other human experts, standard-of-care processes, alternative LLM systems or no comparator in single-arm evaluations.

Primary research studies of any design were eligible, including experimental, comparative, observational, retrospective, prospective, simulation-based and vignette-based studies, provided that they reported outcomes relevant to LLM use in rheumatology. Non-data publications, editorials, narrative reviews, letters and other correspondence formats, irrespective of whether they reported original data, case reports and conference papers were excluded.

### Search strategy and study selection

A systematic search of MEDLINE, Embase and Web of Science was conducted from database inception to 8 January 2026 and updated on 13 July 2026 to capture studies published online up to that date. Only published studies were sought, and no restrictions regarding publication date were applied. In accordance with the PROSPERO protocol, only studies published in English were included.

The search strategy combined terms related to LLMs and generative AI with terms related to rheumatology and rheumatic and musculoskeletal diseases. Model-specific terms, including GPT-4 and GPT-3.5, were carried over from the prospectively registered protocol and were combined with generic terms such as ‘large language model’ and ‘generative artificial intelligence’; these generic terms ensured the retrieval of studies evaluating models released after protocol registration, and eight studies of GPT-5-based systems were captured in this way. Individual disease entities that were not named explicitly in the search string, such as osteoarthritis, gout and other crystal arthropathies and fibromyalgia, were intended to be captured by the broader terms ‘arthritis’ and ‘rheumatol*’. Records indexed as animal studies were removed using a standard animal filter. Potentially eligible reports that were excluded after full-text assessment, together with the specific reasons for exclusion, are provided in online supplemental table 3 in accordance with PRISMA 2020 item 16b. The complete search strategies for all databases, including the animal filter and all model-specific terms, are provided in online supplemental material 1.

The retrieved publications were imported to Rayyan,^17^ duplicates were deleted, and titles and abstracts were screened independently by PK and ZB, with uncertainties being resolved by a third reviewer. The full text of selected abstracts was assessed for eligibility by PK and ZB, with disagreements resolved by a third reviewer (MK). Several included studies were co-authored by PK or MK; screening, extraction and risk-of-bias assessment for these reports were performed by ZB, who is not an author of any of these reports. A residual reviewer conflict can nevertheless not be fully excluded.

### Data extraction

For each included study, OpenAI’s GPT-5.4, assessed through the web interface, generated an initial structured extraction for each report (reasoning set to ‘high’, full prompt in online supplemental material 1), as recently reported^18^: bibliographic information, publication year, country, study design, rheumatologic disease, study objective, evaluated LLM or LLM-based system, model version, input data or prompt type, comparator, outcome domain, outcome metric, sample size and numerical results were extracted.

Studies that did not name a specific disease entity, such as evaluations covering rheumatology in general, were categorised as ‘disease not specified’; studies explicitly addressing two or more named entities were categorised as ‘mixed diseases’. Each study was assigned to exactly one of six application domains (patient education, clinical decision support, medical education, conversational agents or patient-facing interaction, text analysis or information extraction, research support or evidence synthesis) based on the intended user and purpose of the evaluated system.

Where studies reported multiple outcomes, each was extracted separately and assigned to one of 19 predefined domains: performance and validity (accuracy, diagnostic or classification performance, reliability or agreement and consistency or reproducibility), content quality (quality, completeness or usefulness; guideline concordance; readability or comprehensibility; response length or verbosity and citation or reference quality), safety (safety or misinformation and risk appetite), user-oriented and implementation outcomes (usability, acceptability or adoption; attitudes or trust; empathy; sentiment or emotion; efficiency or productivity and cost) and clinical outcomes (disease activity and patient-reported outcomes). Outcome-domain counts were not mutually exclusive. For clinical outcome domains, we distinguished three levels: clinical variables used as model targets (eg, prediction, classification or extraction of disease activity from clinical text), clinical or patient-reported endpoints measured within a study and downstream outcomes attributable to LLM-supported care. Real-world clinical data were defined as patient-level data from clinical practice (electronic health records, medical records, pathology or imaging reports). This criterion describes the provenance of the data, irrespective of whether they were used retrospectively or prospectively and does not imply workflow-embedded evaluation. Given uncertainty about the quality of the LLM-assisted extraction, two reviewers (PK and ZB) independently verified every extracted field against the source publication and performed corrections if discrepancies occurred. Disagreements between the reviewers were resolved through discussion or adjudication by a third reviewer.

### Risk of bias and quality assessment

Risk of bias and methodological quality were assessed independently with the Alberta Quality Assessment Tool: Risk of Bias (AQAT:RoB).^19^ AQAT:RoB was selected because the included studies primarily consisted of heterogeneous LLM evaluations and benchmarking studies, for which a unified AI-specific tool was considered most appropriate. AQAT:RoB consists of five AQAT:RoB domains comprising nine assessable items (questions (items 1–3): question source, question selection and question manipulation; reference answers (items 4–5): reference answer source and reference answer selection; LLM answers (item 6): LLM answer selection; evaluators (items 7–8): evaluator selection and blinding of evaluators; outcomes (item 9): performance metrics), each item rated as low, high or unclear risk of bias. In cases of missing or partial reporting of a specific item, the rating of 'unclear’ was assigned. In addition to the item-level assessments, we calculated an exploratory review-level classification using a conservative worst-item rule. Studies were rated as high risk if at least one item was judged high risk, unclear risk if at least one item was unclear and no item was high risk, and low risk only if all items were judged low risk. In a post hoc analysis, overall risk-of-bias judgements were cross-tabulated against the translational maturity level. Studies to which AQAT:RoB does not apply are shown as a separate ‘not assessable’ category.

### Data synthesis

Each study was assigned post hoc to the highest level reached within a hierarchical translational maturity framework adapted from previously reported clinical AI evaluation frameworks.^20 21^ The framework ranges from prompt-response benchmarking through reference or guideline comparison, human expert comparison and use of real-world clinical data to prospective workflow evaluation and randomised clinical trial evidence. Given the heterogeneity of the included studies in tasks, models, comparators and metrics, findings were synthesised narratively and summarised quantitatively without meta-analysis. For outcome domains with a sufficient number of comparable quantitative estimates—accuracy in clinical decision support, Flesch-Kincaid Grade Level and guideline concordance—study-level estimates were summarised as medians with ranges and IQRs. The Flesch-Kincaid Grade Level is a readability formula derived from average sentence length and average number of syllables per word that expresses the difficulty of a text as a US school grade level; higher values indicate more demanding text, and a level of grade 6 or below is widely recommended for patient-facing health information.^22^ One value was taken per study: where several models were evaluated, the best-performing model was used for accuracy and guideline concordance, and the median across models for readability. Study-level values, the metric used and the reasons for exclusion are given in online supplemental table 2. All statistical analyses were performed using RStudio software (R Foundation for Statistical Computing, V.4.5.2).

## Results

### Study selection

The systematic database search identified 2184 records, comprising 362 records from MEDLINE, 329 from Web of Science and 1493 from Embase. After the removal of 609 duplicate records, 1575 records underwent title and abstract screening. Of these, 1465 records were excluded, leaving 110 reports for full-text assessment. No reports were unavailable for retrieval. Following full-text assessment, 17 reports were excluded because they did not meet the predefined inclusion criteria, including 16 reports with an ineligible publication or study design and one report that was not related to rheumatology. Overall, 93 studies were included in the final analysis.

The study selection process is summarised in the PRISMA flow diagram (figure 1).

**Figure 1:**
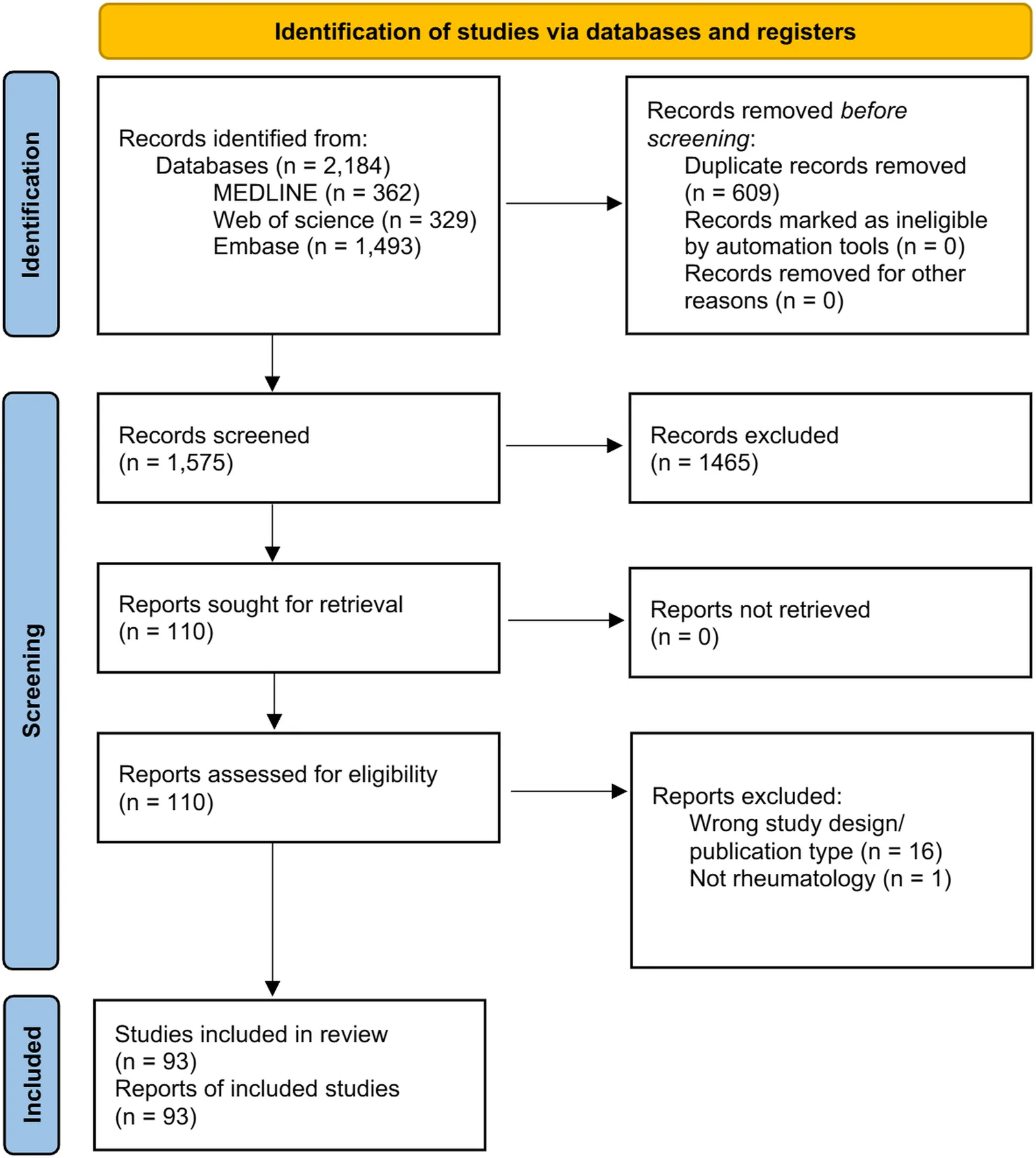
Flow chart showing the selection process of the systematic review. Preferred Reporting Items for Systematic Reviews and Meta-Analyses flow diagram for study selection: 2184 records identified (MEDLINE, n=362; Web of Science, n=329; and Embase, n=1493), 1575 records screened by title and abstract after removal of duplicates, 110 reports assessed in full text and 93 studies included.

### Characteristics

The 93 included studies were published between 2023 and 2026. Most studies were published in 2026 (n=44, 47.3%) and 2025 (n=35, 37.6%), with 11 (11.8%) published in 2024 and 3 (3.2%) in 2023. In total, 71 studies (76.3%) were comparative evaluations, five (5.4%) cross-sectional studies, five (5.4%) prospective studies, four (4.3%) retrospective studies, two (2.2%) validation studies, one (1.1%) a cross-sectional survey, and five (5.4%) were classified as other or unclear designs. Only two studies randomly allocated participants to an LLM-based intervention (2.2%).

The studies covered a broad range of rheumatic and musculoskeletal diseases. The most frequent categories were diseases not further specified (n=26, 28.0%), osteoarthritis (n=15, 16.1%), mixed rheumatic diseases (n=10, 10.8%), systemic lupus erythematosus (n=9, 9.7%), axial spondyloarthritis (n=9, 9.7%) and rheumatoid arthritis (n=7, 7.5%) (figure 2a).

**Figure 2:**
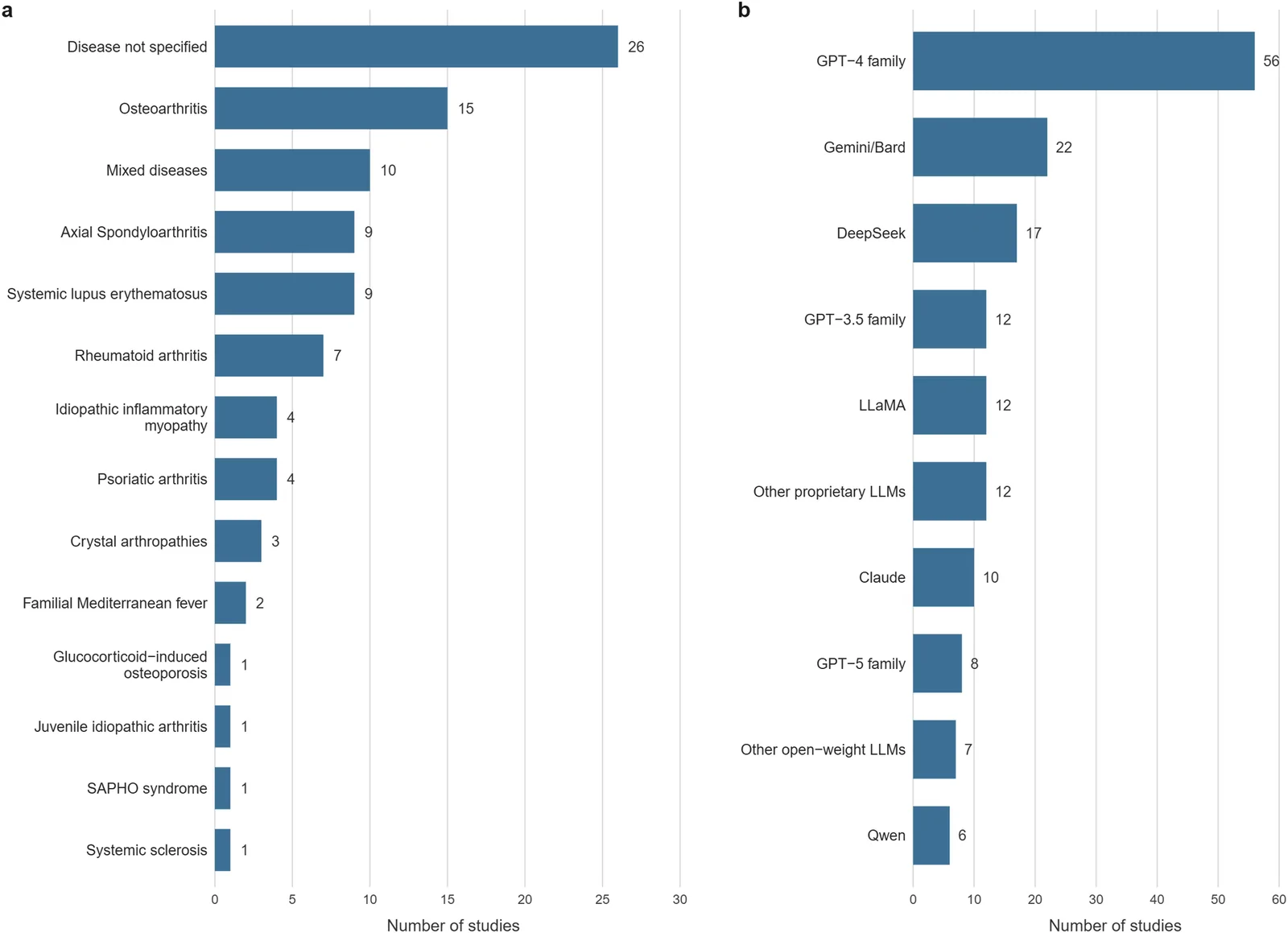
Landscape of LLMs in rheumatology. (**a**) Rheumatic and musculoskeletal diseases addressed by the 93 included studies, most frequently diseases not further specified (n=26, 28.0%) and osteoarthritis (n=15, 16.1%). (**b**) Model families evaluated across the 93 studies. Counts are not mutually exclusive because individual studies frequently evaluated more than one model. Proprietary models evaluated in fewer than three studies each are pooled as a single category (n=12, 12.9%), as are open-weight models (n=7, 7.5%). Non-generative comparators (eg, gradient-boosting or logistic-regression baselines and traditional decision-support tools) and six studies (6.5%) without a reported ChatGPT version are not displayed. LLMs, large language models; SAPHO, synovitis, acne, pustulosis, hyperostosis and osteitis.

### Model landscape

Because individual studies frequently evaluated multiple models, model-family counts were not mutually exclusive. GPT-4-based systems, including ChatGPT-4, GPT-4o and GPT-4 variants, were evaluated in 56 studies (60.2%). Gemini or Bard was evaluated in 22 studies (23.7%), DeepSeek in 17 (18.3%), GPT-3.5 and LLaMA-based systems in 12 each (12.9%), Claude in 10 (10.8%), the GPT-5 family in 8 (8.6%) and Qwen in 6 (6.5%). A further 12 studies (12.9%) evaluated other proprietary models with fewer than three studies each (Perplexity, Copilot/Bing, Grok, Kimi and OpenAI o-series reasoning models). GPT-4o was counted within the GPT-4 family, whereas the o-series reasoning models were counted separately from that family because they represent a distinct reasoning-model line; both are proprietary systems, and seven studies (7.5%) evaluated open-weight models pooled as a single category (including Mistral/Mixtral, Gemma, GLM, Meditron, Falcon, Yi, Baichuan, Orca, HuatuoGPT and Korean-language models); most of the latter derived from two open-weight benchmarking studies. Systems that did not use generative language models, such as gradient-boosting or logistic-regression baselines and traditional diagnostic decision-support tools, were not counted as LLMs and are therefore not represented in the model landscape. In six studies (6.5%), the ChatGPT version was not reported and could therefore not be assigned to a model generation (not shown) (figure 2b)*.*

### Application and outcome domain

Patient education represented the largest application domain (n=39, 41.9%), closely followed by clinical decision support (n=38, 40.9%). Medical education was investigated in seven studies (7.5%), while conversational agents or other patient-facing interactions and text analysis or information extraction were each evaluated in four studies (4.3%). Research support or evidence synthesis was represented by one study (1.1%). The evidence map shows that the literature concentrated on a limited number of application–outcome combinations, with patient education and clinical decision support and their corresponding outcome domains accounting for the large majority of the evidence (figure 3).

**Figure 3:**
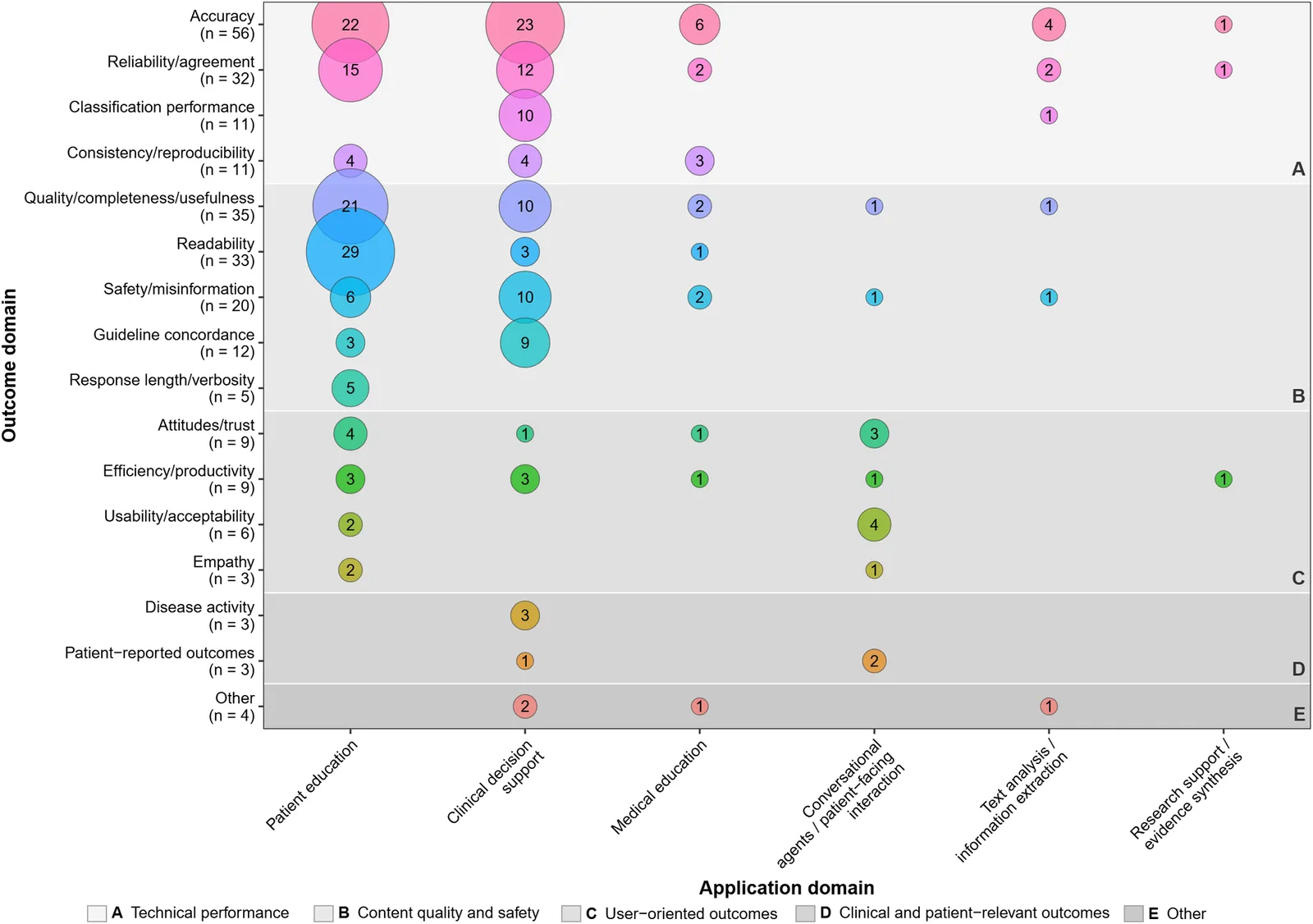
Bubble map displaying outcome domains across applications. Distribution of reported outcome domains across the application domains of the 93 included studies. Bubble size is proportional to the number of studies contributing to each application–outcome combination. Each study was assigned to exactly one application domain, so the application domains are mutually exclusive and sum to 93. Outcomes were assigned to 19 predefined domains, and individual studies could contribute outcomes to several domains, so outcome-domain counts are not mutually exclusive and sum to more than 93. The total number of studies contributing to each outcome domain is shown on the y-axis. For visual organisation, outcome domains were grouped into five conceptual categories: (**A**) technical performance, (**B**) content quality and safety, (**C**) user-oriented outcomes, (**D**) clinical and patient-relevant outcomes and (**E**) other outcomes. Patient education and clinical decision support dominate the application domains, and accuracy-related outcomes dominate the outcome domains, whereas patient-relevant clinical outcomes (disease activity, validated patient-reported outcomes) contribute only isolated studies. Disease activity appears here exclusively as a model prediction or scoring target, as does the validated instrument contributing to the patient-reported outcome domain in one of the three studies in that domain; measurements taken after large language model use are confined to the remaining two patient-reported outcome studies.

Patient education studies most frequently assessed the LLM-generated patient information material for readability, accuracy, quality, completeness or usefulness and reliability or agreement. Clinical decision-support studies were predominantly concentrated around accuracy-based outcomes but also commonly evaluated reliability or agreement, quality, completeness or usefulness, safety or misinformation, classification performance and guideline concordance. Medical education studies primarily assessed accuracy of answers to examination or board-style questions, while studies of conversational agents or other patient-facing interactions focused mainly on usability or acceptability and attitudes or trust. Text-analysis and information-extraction studies predominantly evaluated accuracy.

Across all studies, the most frequently reported study-level outcome domain was accuracy (n=56, 60.2%), followed by quality, completeness or usefulness (n=35, 37.6%), readability (n=33, 35.5%), reliability or agreement (n=32, 34.4%) and safety or misinformation (n=20, 21.5%). Guideline concordance was assessed in 12 studies (12.9%), while classification performance and consistency or reproducibility were each evaluated in 11 studies (11.8%). Less frequently reported domains included attitudes or trust (n=9, 9.7%), efficiency or productivity (n=9, 9.7%), usability or acceptability (n=6, 6.5%), response length or verbosity (n=5, 5.4%) and empathy (n=3, 3.2%). The four least frequently reported outcome domains—sentiment or emotion, citation or reference quality, risk appetite and cost—were each assessed by a single study (1.1% each). Only three studies (3.2%) assessed disease activity in each case as a prediction or scoring target for the model rather than as an outcome of LLM use. Outcomes were assigned to the patient-reported outcome domain in three studies (3.2%); in one of these, a validated instrument was used only as a prediction target, so that validated patient-reported outcome measures were applied as study outcomes in two studies (2.2%).

### Methodological reporting

Methodological reporting was heterogeneous across the included studies. When studies were mapped according to their highest level of clinical validation maturity, most remained at the level of prompt-response benchmarking or reference-standard comparison (figure 4a). 31 studies (33.3%) were classified as prompt-response benchmarks, 14 (15.1%) as reference or guideline comparisons, and 24 (25.8%) included human expert comparison. 18 studies (19.4%) reached the level of real-world clinical data as their highest category; four further studies using such data were assigned to higher levels, so that 22 studies (23.7%) used real-world clinical data overall; in most of these, such data were analysed retrospectively. Only six studies (6.5%) reached the top two categories: four (4.3%) prospectively evaluated an LLM-based application in a clinical or patient-facing workflow, and two (2.2%) randomly allocated participants to an LLM-based intervention versus a comparator.

**Figure 4:**
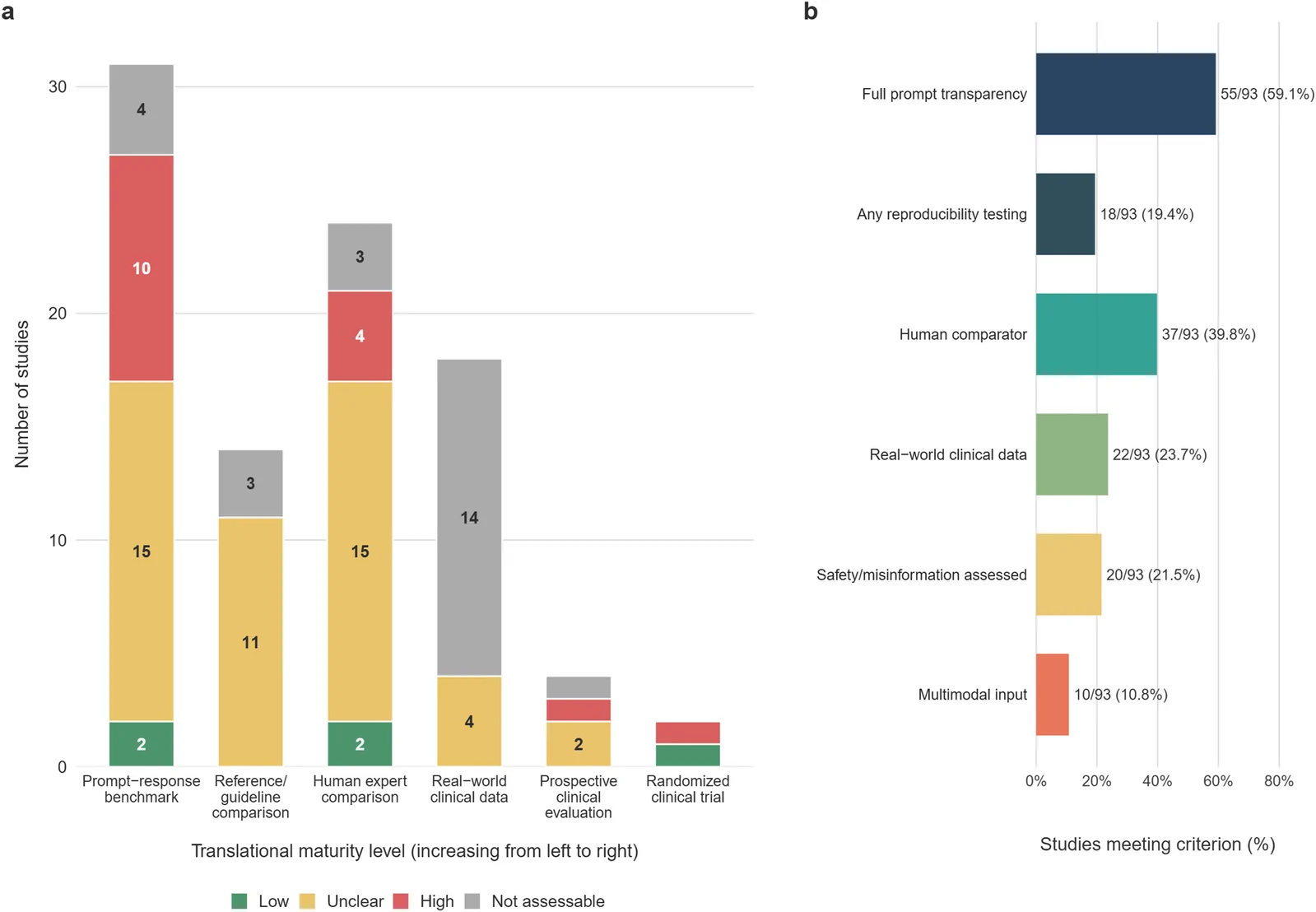
Translational maturity, risk of bias and implementation readiness of LLM studies in rheumatology. (a) Number of included studies by highest translational maturity level reached, stratified by overall AQAT:RoB judgement. Each of the 93 studies was counted once and assigned hierarchically, from prompt-response benchmarking to randomised clinical trial evidence, using a review-derived post hoc framework. ‘Not assessable’ denotes studies without human evaluation of free-text model answers, to which AQAT:RoB does not apply (n=25). (b) Clinical-readiness indicators across all 93 studies, shown as the proportion meeting each criterion. Counts are independent of the hierarchical assignment in panel a; 22 studies used real-world clinical data in total, of which 18 had this as their highest maturity level, and four reached higher levels. AQAT:RoB, Alberta Quality Assessment Tool: Risk of Bias; LLM, large language model.

Complete prompt information was reported in 55 studies (59.1%), partial information in 27 studies (29.0%), and absent or unclear information in 11 studies (11.8%) (figure 4b). Repeated-run or test–retest evaluation was reported in 18 studies (19.4%), whereas 75 studies (80.6%) reported no reproducibility assessment. 37 studies (39.8%) included a human comparator, and 20 studies (21.5%) assessed safety-related outcomes, including misinformation. 10 studies (10.8%) evaluated image-based or multimodal input, 81 studies (87.1%) used text-only input, and two studies (2.2%) used mixed or other input modalities. Agreement between the study design reported by the authors and our harmonised design classification was limited, as 53 of 93 studies (57.0%) were classified differently in our final taxonomy.

### Quantitative outcome synthesis

Table 1 summarises the study-level estimates for the outcome domains with sufficient quantitative reporting; the individual outcome domains are described in detail below. 17 of the 38 clinical decision-support studies reported an interpretable overall accuracy estimate; nine were excluded because accuracy was reported only on ordinal rating scales, only as agreement coefficients, or without an overall estimate. The median accuracy of the best-performing model per study was 86.3% (IQR 50.0%–95.4%). Accuracy was strongly task-dependent. It was highest for criteria-based classification or structured diagnostic tasks with complete clinical information (six studies; median, 90.9%; range, 76.8%–97.0%) and for knowledge or benchmark questions (four studies; median, 91.2%; range, 85.9%–100%). Performance was substantially lower for image-based grading (two studies; 30.4% and 66.0%) and open-ended free-text differential diagnosis assessed using a top-1 criterion (five studies; median, 40.0%; range, 35.0%–100%), although the upper estimate was based on only nine vignettes.^23^

**Table 1:** Quantitative synthesis of reported outcomes across the included studies

Outcome parameter	Studies or comparisons contributing, n	Summary value	Variability or additional detail
Accuracy in clinical decision support, by task type*—*median % (IQR or range)			
All studies with an overall accuracy estimate (best-performing model per study)	17	86.3	IQR 50.0–95.4
Criteria-based classification or structured diagnosis	6	90.9	76.8–97.0
Knowledge or benchmark questions	4	91.2	85.9–100
Image-based grading	2	48.2	30.4 and 66.0
Free-text differential diagnosis (top-1 criterion)	5	40.0	35.0–100; upper value from nine cases only
Readability of patient-facing output*—*Flesch-Kincaid Grade Level			
Median grade level	13	12.2	IQR 11.6–13.7
Studies at or below grade 6 (recommended target), n (%)	13	0 (0)	—
Studies at or below grade 8, n (%)	13	1 (8)	Purpose-built custom model, grade 7.8
Studies above grade 12, n (%)	13	7 (54)	—
Guideline concordance*—*median % (IQR or range)			
Best-performing model per study	10	75.5	IQR 71.8–80.5
Worst-performing model in multimodel studies	6	50.0	32.0–60.0 on the same questions
Studies with concordance ≥90%, n (%)	10	2 (20)	—
Studies with concordance ≥80%, n (%)	10	4 (40)	—
Studies with concordance ≥70%, n (%)	10	8 (80)	—
Direction of effect versus a human comparator (20 paired comparisons from 15 studies)—n of comparisons favouring large language model/no clear difference/favouring human			
Diagnostic accuracy versus clinicians	4	1/1/1	One further comparison not directional
Answer quality versus human written or reference material	6	2/2/2	—
Readability versus human written or reference material	7	4/0/3	—
Time or efficiency versus humans	3	3/0/0	Differences of fivefold to 14-fold

One value was taken per study. Where several models were evaluated, the best-performing model was used for accuracy and guideline concordance and the median across models for readability. Per-study values, the metric used and reasons for exclusion are given in online supplemental table 2.

Medians and ranges describe the distribution of study-level estimates and do not represent pooled effects.

LLM, large language model.

Although readability was assessed in 33 studies, a numerical Flesch-Kincaid grade level was reported only in 13 studies. The median was grade 12.2 (IQR 11.6–13.7), which is 6.2 grade levels above the recommended sixth-grade ceiling for patient-facing material. No study reached grade 6, one (8%) reached grade 8, 12 of 13 (92%) exceeded grade 8 and seven of 13 exceeded grade 12. The two lowest values were achieved by approaches specifically designed for simplification, namely an explicit fifth-grade rewrite prompt (grade 8.3)^24^ and a purpose-built custom model (grade 7.8),^25^ both of which still exceeded the sixth-grade target.

10 studies reported guideline concordance as a proportion. The median concordance of the best-performing model was 75.5% (IQR 71.8%–80.5%); concordance was at least 90% in two of 10 studies (20%), at least 80% in four of 10 (40%) and at least 70% in eight of 10 (80%). Across the six studies comparing multiple models, the worst-performing model achieved a median concordance of 50.0% (range 32.0%–60.0%) on the same questions.

Exploratory vote counting of 20 paired comparisons from 15 studies showed a consistent direction of effect only for time or efficiency, where all three comparisons favoured the LLM with differences of fivefold to 14-fold. Direction was inconsistent for diagnostic accuracy, answer quality and readability.

### Application-specific outcome synthesis

Application-specific findings and the studies underlying them are summarised in table 2 and online supplemental table 1. In patient education, LLM-generated responses were frequently rated as informative, and in selected blinded comparisons, they matched or exceeded physician-written or reference material.^10 26–28^ In the Consulting ChatGPT-4 for 100 Frequently Asked Lupus Question study (ChatSLE*s*) by Haase et al., GPT-4 responses achieved higher quality scores than Lupus100.org answers (4.55 vs 4.31; p=0.001), comparable empathy scores (4.14 vs 4.07; p=0.27) and higher rater preference (57% vs 43%; p=0.02).^29^ In clinical decision support, the contrast between structured and open-ended tasks extended to treatment planning, where rheumatology-board plans were preferred in 68.8% of ratings compared with 16.3% for GPT-4 and 15.0% for GPT-3.5.^9^ Safety-relevant deviations occurred even where overall guideline concordance was high: for instance, in hip and knee osteoarthritis, ChatGPT and Bard encouraged non-recommended treatments in 3 of 10 (30%) and 6 of 10 (60%) instances, respectively.^30 31^ Evidence from medical education, multimodal tasks, text analysis and research support remained limited to a small number of studies.^32–34^

**Table 2:** Methodological maturity and RoB by application domain (n=93 studies)

Application domain	Studies, n (%)	Human comparator, n (%)	Real-world clinical data, n (%)	Prospective or randomised design, n (%)	Prompts fully reported/reproducibility tested, n (%)	RoB assessable, n (high/unclear/low)
Patient education	39 (42)	13 (33)	1 (3)	1 (3)	29 (74) / 6 (15)	36—10/23/3
Clinical decision support	38 (41)	19 (50)	16 (42)	0 (0)	20 (53) / 8 (21)	23—4/18/1
Medical education	7 (8)	2 (29)	0 (0)	1 (14)	2 (29) / 2 (29)	4—0/3/1
Conversational agents/patient-facing interaction	4 (4)	0 (0)	4 (100)	4 (100)	1 (25)/0 (0)	3—1/2/0
Text analysis/information extraction	4 (4)	2 (50)	1 (25)	0 (0)	2 (50)/2 (50)	1—1/0/0
Research support/evidence synthesis	1 (1)	1 (100)	0 (0)	0 (0)	1 (100)/0 (0)	1—0/1/0
All studies	93 (100)	37 (40)	22 (24)	6 (6)	55 (59)/18 (19)	68—16/47/5

Percentages are row percentages (denominator=studies in that application domain); the column ‘Studies’ uses n=93 as denominator. Each study is assigned to exactly one application domain.

Human comparator: physician, clinician or expert panel named as comparator for the model output.

Real-world clinical data: patient-level data from clinical practice (electronic health records, medical records, pathology or imaging reports).

Prospective or randomised design: prospective patient- or workflow-level evaluation or random allocation of participants to a large language model-based intervention.

Prompts fully reported: verbatim reproduction of all prompts and test questions in the article or its supplement. Reproducibility tested=repeated querying of the model to assess answer stability.

RoB assessable: number of studies eligible for the Alberta Quality Assessment Tool: Risk of Bias, which applies to studies with human evaluation of free-text model answers; overall judgement follows the worst-item rule.

RoB, risk of bias.

### Risk of bias

Risk of bias was assessed using AQAT:RoB in 68 studies (73.1%) for which the tool was considered applicable. The remaining 25 studies (26.9%) did not include human evaluation of free-text model answers and were classified as not assessable. Across the nine AQAT:RoB items, low risk of bias was most frequent in Item 1 (question source) (54/68, 79.4%) and Item 5 (reference *answer selection*) (46/68, 67.6%). Unclear ratings were most common in Item 9 (performance metrics) (48/68, 70.6%) and Item 8 (blinding *of evaluators*) (34/68, 50.0%), while high risk of bias was most frequent in Item 8 (blinding of evaluators) (11/68, 16.2%), followed by Item 7 (evaluator selection) (10/68, 14.7%) and Item 6 (LLM answer selection) (8/68, 11.8%) (figure 5a). Item-level judgements for each individual study are shown in online supplemental figure 1.

**Figure 5:**
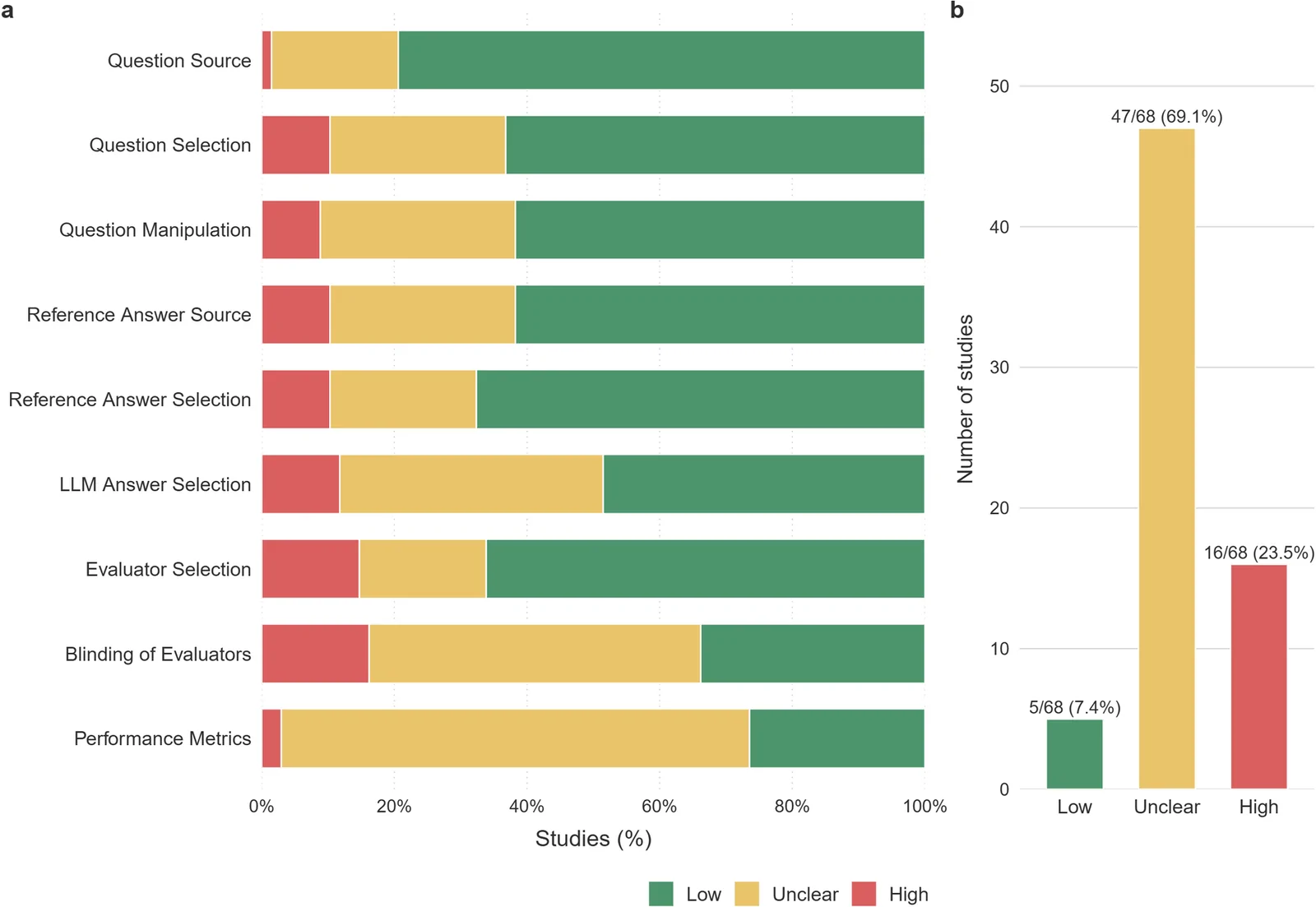
Risk of bias. (a) per Alberta Quality Assessment Tool: Risk of Bias item; (b) overall risk classified by the highest-risk item per study. LLM, large language model.

Among the 68 AQAT:RoB-assessable studies, only five (7.4%) were judged to be at low risk of bias, while 47 studies (69.1%) had an unclear risk and 16 studies (23.5%) were at high risk (figure 5b). Among studies assessable with AQAT:RoB, no clear improvement in risk of bias was observed with increasing translational maturity. However, non-assessability was concentrated at higher maturity levels (14 of 18 studies (77.8%)) (figure 4a). Among assessable studies, low risk of bias was confined to prompt-response benchmarks (n=2), human expert comparisons (n=2) and one randomised trial, whereas no study at the real-world clinical data or prospective evaluation level was rated as low risk.

## Discussion

This systematic review demonstrates a rapidly growing body of research on LLMs in rheumatology, although it remains at an early stage of clinical translation and methodological development. The reviewed literature suggests that LLMs can generate plausible patient-facing information, perform well in selected structured diagnostic tasks and reproduce parts of guideline content, but that most studies relied on text-based prompt–response testing and surrogate outcomes.

Although 22 studies (23.7%) used data derived from routine clinical care, these were largely retrospective records rather than workflow-embedded use, and only four studies (4.3%) prospectively evaluated LLMs within a rheumatology workflow.

Furthermore, disease activity was assessed in only three studies and exclusively as a model prediction or scoring target rather than as an outcome of LLM-supported care. Validated patient-reported outcomes were measured in only two studies, both in exposed participants without a control group. Thus, no included study demonstrated an improvement in downstream clinical outcomes attributable to LLM-supported care.

This gap mirrors the broader medical literature: a recent large-scale review identified 4609 clinical LLM studies, of which 1048 used real-world patient data, and 19 were prospective randomised trials.^18^ The proportion of studies using real-world data in our review (23.7%) exceeded the 5% reported by Bedi *et al*,^13^ but this largely reflects timing rather than a genuine shift towards clinical evaluation. Their search closed before 85% of our included studies were published, and only four studies prospectively collected real-world data. The apparent progress therefore reflects a transition from vignette-based evaluations to retrospective clinical datasets rather than true clinical implementation. Prospective studies of clinician-facing LLM assistance for diagnostic or therapeutic decision-making are beginning to emerge in general medicine, with effects varying by task and setting,^35–37^ but remain absent in rheumatology.

Patient education represented the largest evidence cluster, plausibly reflecting both its relevance to chronic disease self-management and the relative accessibility of text-based patient-information tasks.^38^ LLM-generated material was frequently rated as accurate, empathetic and comparable in quality to physician-written or reference sources.^29 39 40^ Readability, however, remained a recurring limitation, with outputs exceeding recommended reading levels even when simplification was explicitly prompted.^24 40^ A patient-derived benchmark in connective tissue diseases nevertheless reported high understandability, indicating that measured performance depends strongly on prompt design, rater population and metric.^11 41^ This matters clinically because the use of written information depends on patients’ health literacy.^42^

It is important to point out that strong performance in structured tasks should not be equated with clinical reasoning. LLMs can reproduce individual guideline statements,^31 43^ but factual agreement does not establish reliable navigation of conditional care pathways.^44^ Case vignettes with complete information also do not reproduce the uncertainty and incomplete information of real-life care. Accuracy, readability, reliability and guideline concordance remain surrogate measures that do not establish clinical benefit. Consistent with this, a rubric-based benchmark of rheumatology cases, in which 1020 criteria were weighted for clinical appropriateness and potential harm by board-certified rheumatologists, found that no evaluated system exceeded an overall score of 54.0% and that potentially severe errors, predominantly omissions of clinically necessary content, occurred with every system.^45^

Other medical fields have already begun to assess patient-reported outcomes and workflow effects, particularly in mental health and ambient documentation.^46 47^ However, in rheumatology, validated patient-reported outcome measures were used only in two studies.^48 49^ No study assessed treatment adherence, diagnostic delay or patient outcomes as downstream consequences of LLM-supported care, indicating that evidence for the real-world clinical impact of LLMs in rheumatology remains very limited.

Methodological reporting further limited interpretability: complete prompt information was only reported in 55 studies (59.1%), and 75 (80.6%) did not report repeated-run or test–retest evaluation. Because outputs vary with model updates, access date, system prompts, sampling settings and retrieval functions, reproducibility depends on the full evaluation pipeline rather than the prompt alone.^50^ Consistent with this, the reliability of repeated outputs from a generative system has been examined through appraisal by paediatric rheumatologists in a multicentre expert survey^51^—a design that targets output stability rather than task-level performance. Unclear risk of bias ratings were driven mainly by such reporting gaps, whereas high-risk ratings reflected substantive design limitations in comparator choice, evaluator procedures and reproducibility assessment. Unlike the large cross-specialty review, which did not conduct a study-level risk of bias assessment,^18^ we applied AQAT:RoB with a conservative worst-item approach, rating only a minority of AQAT:RoB-assessable studies at low risk of bias (7.4%) and most as unclear (69.1%) or high risk (23.5%). Among studies amenable to AQAT:RoB assessment, risk of bias was therefore predominantly unclear or high, while the applicability of the tool was limited for several more clinically embedded study designs. Design-appropriate reporting guidance should therefore be followed (Consolidated Standards of Reporting Trials–AI (CONSORT-AI) and Standard Protocol Items: Recommendations for Interventional Trials–AI (SPIRIT-AI) for trials and protocols, Transparent Reporting of a multivariable prediction model for Individual Prognosis or Diagnosis-LLM (TRIPOD-LLM) for diagnostic or prognostic model evaluations).^52–54^

For the near term, evaluation should prioritise bounded, auditable and clinician-supervised tasks such as drafting patient information, summarising clinical text or extracting predefined data. The available evidence is insufficient to support autonomous diagnostic or treatment recommendations, and human oversight should not be assumed to ensure safety but treated as an integral component of the intervention and evaluated explicitly in terms of verification burden, residual errors and automation bias. Future studies should therefore progress from retrospective benchmarks to prospective workflow evaluations and, where appropriate, pragmatic or randomised trials assessing task-specific outcomes such as medication safety, patient understanding and clinician workload.

Strengths of this review include the rheumatology-specific full-text synthesis, extraction of study-level and outcome-level data, mapping of application and outcome domains, assessment of translational maturity, and a study-level risk-of-bias assessment where AQAT:RoB was applicable. The substantial heterogeneity of the included studies in tasks, evaluated models, comparators and outcome metrics precluded any formal meta-analysis or pooled quantitative estimate. The synthesis is therefore predominantly narrative, and the strength of the quantitative conclusions that can be drawn is correspondingly limited: the medians reported here describe the distribution of study-level estimates rather than pooled effect estimates, and direct comparisons between studies remain restricted. The rapid evolution of LLMs also challenges the temporal validity of the findings, while incomplete reporting of model versions, prompts, access dates and testing procedures reduced reproducibility. Although every LLM-generated extraction was independently verified against the source publication by two reviewers, the use of an LLM to generate the initial structured draft for each report is a comparatively new approach in systematic reviews and may still have introduced residual errors or misclassifications, particularly for complex tables, units of measurement, outcome definitions and methodological details. Restriction to English-language, peer-reviewed publications may have introduced language and publication bias. In addition, several disease entities were captured only through broader terms such as ‘arthritis’ and ‘musculoskeletal disease’ rather than being named individually in the search string. This may have reduced the sensitivity of the search for conditions such as gout and other crystal arthropathies, fibromyalgia and osteoporosis, and individual eligible reports may therefore have been missed. Because accuracy and guideline concordance were summarised using the best-performing model per study, these study-level summary estimates are best-case values and may overstate typical model performance. In addition, because AQAT:RoB was not applicable to all study designs, particularly several prospective and workflow-embedded evaluations, the risk of bias findings should not be interpreted as a comprehensive quality assessment of the entire evidence base. The conservative worst-item approach to overall judgements may also have resulted in stringent ratings. Finally, AQAT:RoB is itself a recently developed instrument: it was created by a geographically concentrated expert panel, pilot-validated in a small number of reports and evaluated only in English-language publications. Geographical, cultural and language-related biases in the tool, and limited generalisability across the range of study designs included here, can therefore not be excluded.

## Conclusion

LLMs show task-specific performance in rheumatology, particularly in patient education, structured diagnostic tasks and guideline-based information retrieval. The evidence base, however, still rests on prompt-response benchmarks with surrogate outcomes, incomplete reporting and, among AQAT:RoB-assessable studies, predominantly unclear or high risk of bias and therefore do not establish clinical effectiveness or safety. Prospective, workflow-embedded evaluations with patient-relevant endpoints and transparent reporting are therefore the decisive next step.

## Supplementary material

10.1136/rmdopen-2026-007538 online supplemental file 1

10.1136/rmdopen-2026-007538 online supplemental file 2

10.1136/rmdopen-2026-007538 online supplemental file 3

## Data Availability

Data are available upon reasonable request. All data relevant to the study are included in the article or uploaded as supplementary information.
