# Supplementary material for: Large language models in rheumatology: applications, methodological quality and clinical translation – a systematic review
Source: RMD Open. 2026 Oct 28;12(4):e007538. doi: 10.1136/rmdopen-2026-007538 (PMC13723917; doi:10.1136/rmdopen-2026-007538)
Supplement: Supplementary file 1 [file rmdopen-12-4-s002.pdf]

Supplementary R Code

Large language models in rheumatology: applications, methodological quality and clinical translation - a systematic review

---

Journal: RMD Open
Source file: Supplementary_R_Code_LLMs_Rheumatology.R
Code lines: 3,948

Formatting note: The R code is reproduced verbatim. Line numbers were added only for reference. No code statements, comments, spacing, or ordering were edited for this PDF.

SHA-256 of source .R file:

`1e301479b625fdc97f45f24cf2e215e0402d9c6b4cd2ec09c7750e5097e1fc1c`

```
# ============================================================================
# Supplementary R code
# Large language models in rheumatology: a systematic review
#
# This file contains the R code used to generate the figures of the review and
# the descriptive summary tables underlying them. Each figure is produced by one
# self-contained function; sourcing the file defines these functions but does
# not execute any analysis.
#
# ----------------------------------------------------------------------------
# FIGURES
# ----------------------------------------------------------------------------
#   Function                                Figure
#   analysis_bubble_map()                   Application x outcome bubble map
#   analysis_model_landscape()              Model landscape (primary and
#                                           sensitivity version)
#   analysis_disease_landscape()            Disease landscape
#   analysis_rob_domains()                  AQAT:RoB domain-level risk of bias
#   analysis_rob_overall()                  Overall AQAT:RoB risk of bias
#   analysis_rob_combined()                 Domain-level and overall risk of
#                                           bias as panels a and b
#   analysis_rob_heatmap()                  Risk of bias per study and domain
#   analysis_maturity_rob()                 Risk of bias by translational
#                                           maturity
#   analysis_implementation_readiness()     Implementation-readiness indicators
#   analysis_maturity_readiness_combined()  Maturity and readiness as panels
#                                           a and b
#
# ----------------------------------------------------------------------------
# DENOMINATORS
# ----------------------------------------------------------------------------
# The review included 93 studies. The AQAT:RoB tool was applicable to 68 of
# them; the remaining 25 studies fall outside the scope of the tool and are
# deliberately not forced into the risk-of-bias figures. Accordingly, all
# risk-of-bias figures use n = 68 and all other figures use n = 93. Both
# denominators are asserted at run time and a deviating input aborts the
# analysis rather than producing a silently rescaled figure.
#
# ----------------------------------------------------------------------------
# REQUIREMENTS
# ----------------------------------------------------------------------------
# R (>= 4.1) and the packages readxl, dplyr, tidyr, ggplot2, stringr, scales,
# forcats and patchwork. This file checks for them but never installs them:
#
#   install.packages(c("readxl", "dplyr", "tidyr", "ggplot2", "stringr",
#                      "scales", "forcats", "patchwork"))
#
# ----------------------------------------------------------------------------
# INPUT DATA
# ----------------------------------------------------------------------------
# The figures are generated from prepared Excel workbooks. Expected file names,
# sheets and columns:
#
# Application_Outcome_BubbleMap_Input_Data_Rheumatology_LLMs.xlsx
#   sheet Bubble_Map_Data   Application_domain, Outcome_domain, n_studies,
#                           n_outcome_rows, Study_IDs
#   sheet Category_Orders   Application_order, Outcome_order
#
# Model_Landscape_Data.xlsx
#   sheet Model_Family_Summary   Model_Family, n_studies,
#                                Include_in_primary_plot
#
# Disease_Landscape_Data.xlsx
#   sheet Disease_Summary_Primary   Disease_Category, n_studies,
#                                   Include_in_primary_plot, optional Plot_Order
#
# Implementation_Readiness_Input.xlsx
#   first sheet    indicator_order, indicator, n_studies, percent (0-100)
#                  In the workbook used here this is Implementation_Indicators.
#   sheet README   field, value; one row with field == "n_total"
#
# Maturity_RoB_Input.xlsx
#   first sheet    level_order, maturity_level, rob_overall, n_studies
#                  In the workbook used here this is Maturity_RoB.
#
# RoB_rawdata_and_rscript_input.xlsx
#   sheet Risk_of_Bias_Raw   Study_ID, Tool_1 (a column named "Tool 1" is
#                            accepted), Domain_1 to Domain_9
#   Studies are selected on Tool_1; the values "AQAT" and "AQAT:RoB" are both
#   recognised, whereas "AQAT doesnt fit" marks the studies outside the scope of
#   the tool. File name and sheet have no default and must be passed to the
#   analysis functions, because the risk-of-bias assessments were maintained in
```

```r
#   a separate workbook.
#
# Colours are defined in this file, not read from the workbooks. Where an input
# workbook carries its own colour column, that column is not used.
#
# -----------------------------------------------------------------------------
# DATA AVAILABILITY
# -----------------------------------------------------------------------------
# The input workbooks described above are not included with this supplement.
# They are available from the corresponding author on reasonable request. This
# file documents how the figures of the review were generated from those
# workbooks; it is not intended to run without them.
#
# -----------------------------------------------------------------------------
# HOW TO RUN
# -----------------------------------------------------------------------------
# 1. Place the workbooks listed above in a folder, for example "data".
# 2. source("Supplementary_R_Code_LLMs_Rheumatology.R", encoding = "UTF-8")
# 3. Run the figures for which the inputs are available, for example:
#
#      result <- run_review_analysis(
#        sections = "bubble_map",
#        data_dir = "data"
#      )
#
#      result <- run_review_analysis(
#        sections  = "rob_combined",
#        data_dir  = "data",
#        rob_file  = "RoB_rawdata_and_rscript_input.xlsx",
#        rob_sheet = "Risk_of_Bias_Raw"
#      )
#
#    sections = "all" runs every figure. A sheet may be given by name or by
#    numeric index.
#
# Each function returns the figure object together with the summary table it is
# based on, so that every plotted number can be inspected. The model landscape,
# disease landscape and risk-of-bias heatmap are additionally written to
# output_dir as PDF, PNG and, where applicable, TIFF; the remaining figures are
# returned for export with ggsave(). run_review_analysis() also writes the
# sessionInfo() of the run to output_dir.
#
# -----------------------------------------------------------------------------
# NOTES ON THE ANALYSIS
# -----------------------------------------------------------------------------
# - Overall risk of bias is derived from the domain ratings by the worst-domain
#   rule: High if any domain is High, otherwise Unclear if any domain is
#   unclear, not assessable or missing, otherwise Low. Unrecognised entries are
#   treated conservatively as Unclear.
# - Bubble-map counts are taken from the prepared input and verified against the
#   study identifiers listed for each combination. Repeated application x
#   outcome combinations are merged by taking the union of their identifiers.
# - Study counts in the model landscape are not mutually exclusive, because a
#   single study may evaluate more than one model family.
# - Implementation-readiness percentages are checked against n_studies / 93
#   before plotting.
# =============================================================================

# -----------------------------------------------------------------------------
# Packages
# -----------------------------------------------------------------------------

.review_packages <- c("readxl", "dplyr", "tidyr", "ggplot2", "stringr",
                      "scales", "forcats", "patchwork")

.review_missing <- .review_packages[
  !vapply(.review_packages, requireNamespace, logical(1), quietly = TRUE)
]

if (length(.review_missing)) {
  stop("Install the following packages before sourcing this file: ",
       paste(.review_missing, collapse = ", "), call. = FALSE)
}

library(readxl)
library(dplyr)
library(tidyr)
library(ggplot2)
library(stringr)
library(scales)
library(forcats)
library(patchwork)
```

```r


# ----------------------------------------------------------------------------
# Review denominators
# ----------------------------------------------------------------------------

# Studies included in the review.
REVIEW_N_STUDIES <- 93L

# Studies assessable with the AQAT:RoB tool.
REVIEW_N_AQAT_ROB <- 68L

# Identifies the studies assessed with AQAT:RoB. Case and punctuation are
# normalised first, so that "AQAT", "aqat" and "AQAT:RoB" are all recognised,
# while a label such as "AQAT doesnt fit" is not.
.review_is_aqat_rob <- function(x) {
  normalised <- gsub("[^a-z0-9]", "", tolower(trimws(as.character(x))))
  normalised %in% c("aqat", "aqatrob")
}

.review_study_ids <- function(x) {
  x <- as.character(x)
  x <- x[!is.na(x)]
  ids <- trimws(unlist(strsplit(x, "\\s*[;,]\\s*")))
  unique(ids[nzchar(ids)])
}

.review_assert_count <- function(actual, expected, label) {
  if (length(actual) != 1L || is.na(actual) || actual != expected) {
    stop(
      label, " must contain ", expected, " studies for the final review, but ",
      actual, " were found.", call. = FALSE
    )
  }
  invisible(TRUE)
}

.review_validate_readiness <- function(data, n_total, tolerance = 0.11) {
  if (any(!is.finite(data$n_studies)) || any(data$n_studies < 0) ||
      any(data$n_studies > n_total)) {
    stop("Implementation-readiness n_studies values must range from 0 to ",
         n_total, ".", call. = FALSE)
  }
  if (any(!is.finite(data$percent)) || any(data$percent < 0) ||
      any(data$percent > 100)) {
    stop("Implementation-readiness percent values must range from 0 to 100.",
         call. = FALSE)
  }
  expected_percent <- 100 * data$n_studies / n_total
  if (any(abs(data$percent - expected_percent) > tolerance)) {
    stop(
      "Implementation-readiness percentages are inconsistent with n_studies/",
      n_total, ".", call. = FALSE
    )
  }
  invisible(TRUE)
}

.review_validate_maturity <- function(data) {
  if (any(!is.finite(data$n_studies)) || any(data$n_studies < 0)) {
    stop("Maturity/RoB n_studies values must be finite and non-negative.",
         call. = FALSE)
  }
  if (any(is.na(data$maturity_level)) || any(is.na(data$rob_overall))) {
    stop("Maturity level and overall RoB must be present and use recognised labels.",
         call. = FALSE)
  }
  .review_assert_count(
    sum(data$n_studies),
    REVIEW_N_STUDIES,
    "Maturity/RoB study-count matrix"
  )
}

.review_input <- function(data_dir, filename) {
  path <- file.path(data_dir, filename)
  if (!file.exists(path)) stop("Required input file not found: ", path, call. = FALSE)
  path
}

.review_read_rob <- function(data_dir, filename, sheet) {
  if (is.null(filename) || length(filename) != 1L || !nzchar(filename)) {
```

```r
    stop("The risk-of-bias workbook has no default. Supply it via rob_file ",
         "(or heatmap_file for the heatmap).", call. = FALSE)
  }
  if (is.null(sheet) || length(sheet) != 1L || is.na(sheet)) {
    stop("Specify the sheet of ", filename, " via rob_sheet (or heatmap_sheet), ",
         "by name or numeric index.", call. = FALSE)
  }
  readxl::read_excel(.review_input(data_dir, filename), sheet = sheet)
}

# Reads the logical columns of the input workbooks. Workbooks edited in a
# German locale may contain WAHR/FALSCH or JA/NEIN instead of TRUE/FALSE.
# Anything not recognised as true is treated as FALSE.
.review_as_logical <- function(x) {
  normalised <- tolower(trimws(as.character(x)))
  out <- normalised %in% c("true", "t", "yes", "y", "1", "wahr", "ja")
  out[is.na(x)] <- FALSE
  out
}

# Shared theme of the horizontal bar charts (model and disease landscape).
.review_theme_bar <- function(base_size = 11) {

  theme_minimal(base_size = base_size) +
    theme(
      # No title inside the figure
      plot.title = element_blank(),
      plot.subtitle = element_blank(),
      plot.caption = element_blank(),

      # Grid lines
      panel.grid.major.y = element_blank(),
      panel.grid.minor = element_blank(),

      panel.grid.major.x = element_line(
        colour = "grey85",
        linewidth = 0.35
      ),

      # Axis titles
      axis.title.x = element_text(
        size = base_size,
        colour = "grey20",
        margin = margin(t = 8)
      ),

      axis.title.y = element_blank(),

      # Axis labels
      axis.text.x = element_text(
        size = base_size - 2,
        colour = "grey25"
      ),

      axis.text.y = element_text(
        size = base_size - 1,
        colour = "grey15"
      ),

      # Remove ticks and legend
      axis.ticks = element_blank(),
      legend.position = "none",

      # White background
      plot.background = element_rect(
        fill = "white",
        colour = NA
      ),

      panel.background = element_rect(
        fill = "white",
        colour = NA
      ),

      # Additional right margin for the value labels
      plot.margin = margin(
        t = 8,
        r = 34,
        b = 8,
        l = 8
      )
    )
```

```
}


# ==============================================================================
# SECTION: bubble_map
# ==============================================================================
analysis_bubble_map <- function(data_dir = "data", output_dir = "output") {

  if (!dir.exists(output_dir)) {
    dir.create(output_dir, recursive = TRUE)
  }

  # ============================================================
  # Bubble Map: LLMs in Rheumatology Systematic Review
  # ============================================================
  # Input:
  # Application_Outcome_BubbleMap_Input_Data_Rheumatology_LLMs.xlsx
  #
  # Sheets:
  # - Bubble_Map_Data
  # - Category_Orders
  #
  # Required columns:
  # - Application_domain
  # - Outcome_domain
  # - n_studies
  # - n_outcome_rows
  # - Study_IDs
  #
  # Figure settings:
  # - Bubble size = number of distinct studies
  # - Standard ggplot2 discrete colour palette
  # - No legend
  # - No title or subtitle
  # - No automatic export
  # ============================================================


  # ------------------------------------------------------------
  # 1. Define input file
  # ------------------------------------------------------------

  input_file <- .review_input(
    data_dir,
    "Application_Outcome_BubbleMap_Input_Data_Rheumatology_LLMs.xlsx"
  )


  # ------------------------------------------------------------
  # 2. Load bubble-map data
  # ------------------------------------------------------------

  Application_Outcome_BubbleMap_Input_Data_Rheumatology_LLMs <-
    read_excel(
      input_file,
      sheet = "Bubble_Map_Data"
    )

  bubble_df <-
    Application_Outcome_BubbleMap_Input_Data_Rheumatology_LLMs


  # ------------------------------------------------------------
  # 3. Load predefined category order
  # ------------------------------------------------------------

  category_orders <- read_excel(
    input_file,
    sheet = "Category_Orders"
  )

  application_order <- category_orders %>%
    pull(Application_order) %>%
    na.omit() %>%
    as.character() %>%
    str_squish() %>%
    unique()

  outcome_order <- category_orders %>%
    pull(Outcome_order) %>%
    na.omit() %>%
    as.character() %>%
```

```
    str_squish() %>%
    unique()


  # ------------------------------------------------------------
  # 4. Check required columns
  # ------------------------------------------------------------

  required_columns <- c(
    "Application_domain",
    "Outcome_domain",
    "n_studies",
    "n_outcome_rows",
    "Study_IDs"
  )

  missing_columns <- setdiff(
    required_columns,
    names(bubble_df)
  )

  if (length(missing_columns) > 0) {
    stop(
      paste0(
        "The following required columns are missing: ",
        paste(
          missing_columns,
          collapse = ", "
        )
      )
    )
  }


  # ------------------------------------------------------------
  # 5. Clean data
  # ------------------------------------------------------------

  bubble_df <- bubble_df %>%
    mutate(
      Application_domain = str_squish(
        as.character(Application_domain)
      ),

      Outcome_domain = str_squish(
        as.character(Outcome_domain)
      ),

      n_studies = as.numeric(
        n_studies
      ),

      n_outcome_rows = as.numeric(
        n_outcome_rows
      ),

      Study_IDs = as.character(
        Study_IDs
      )
    )

  # Confirm that the complete bubble-map input represents all included studies.
  study_id_lists <- lapply(
    bubble_df$Study_IDs,
    .review_study_ids
  )
  listed_counts <- lengths(study_id_lists)

  if (any(is.na(bubble_df$n_studies)) ||
      any(listed_counts != bubble_df$n_studies)) {
    stop(
      "Bubble-map n_studies values do not match the unique IDs in Study_IDs.",
      call. = FALSE
    )
  }

  .review_assert_count(
    length(unique(unlist(study_id_lists))),
    REVIEW_N_STUDIES,
    "Bubble-map unique Study_ID set"
  )

```

```
# ------------------------------------------------------------
# 6. Retain any additional categories from the data
# ------------------------------------------------------------

application_order <- unique(
  c(
    application_order,
    bubble_df$Application_domain
  )
)

outcome_order <- unique(
  c(
    outcome_order,
    bubble_df$Outcome_domain
  )
)


# ------------------------------------------------------------
# 7. Combine duplicate combinations if present
# ------------------------------------------------------------

bubble_df <- bubble_df %>%
  group_by(
    Application_domain,
    Outcome_domain
  ) %>%
  summarise(
    # Recount from the union of the listed Study_IDs, so that duplicate rows
    # for one combination are merged rather than counted twice.
    n_studies = length(
      .review_study_ids(Study_IDs)
    ),

    n_outcome_rows = sum(
      n_outcome_rows,
      na.rm = TRUE
    ),

    Study_IDs = paste(
      .review_study_ids(Study_IDs),
      collapse = "; "
    ),

    .groups = "drop"
  ) %>%
  mutate(
    Study_IDs = na_if(
      Study_IDs,
      ""
    )
  )


# ------------------------------------------------------------
# 8. Create complete application x outcome grid
# ------------------------------------------------------------

# This ensures that all application and outcome categories
# remain visible on the axes, even if a specific combination
# contains no study.

full_grid <- expand_grid(
  Application_domain = application_order,
  Outcome_domain = outcome_order
)

bubble_df_complete <- full_grid %>%
  left_join(
    bubble_df,
    by = c(
      "Application_domain",
      "Outcome_domain"
    )
  ) %>%
  mutate(
    n_studies = replace_na(
      n_studies,
      0
    ),
```

```
    n_outcome_rows = replace_na(
      n_outcome_rows,
      0
    ),

    Study_IDs = replace_na(
      Study_IDs,
      ""
    )
  )


# ------------------------------------------------------------
# 9. Prepare plotting data
# ------------------------------------------------------------

plot_df <- bubble_df_complete %>%
  mutate(
    Application_domain = factor(
      Application_domain,
      levels = application_order
    ),

    Outcome_domain = factor(
      Outcome_domain,
      levels = rev(outcome_order)
    ),

    label = if_else(
      n_studies > 0,
      as.character(n_studies),
      ""
    )
  )


# Only draw bubbles for combinations represented by at least
# one study. Empty combinations remain visible through the grid.

plot_df_bubbles <- plot_df %>%
  filter(
    n_studies > 0
  )


# ------------------------------------------------------------
# 10. Create bubble map
# ------------------------------------------------------------

p_bubble <- ggplot(
  data = plot_df_bubbles,
  aes(
    x = Application_domain,
    y = Outcome_domain,
    size = n_studies,
    fill = Outcome_domain
  )
) +

  geom_point(
    shape = 21,
    alpha = 0.70,
    color = "grey25",
    stroke = 0.35
  ) +

  geom_text(
    aes(
      label = label
    ),
    size = 3.5,
    color = "black"
  ) +

  # Fill colours come from the default discrete ggplot2 palette.

  scale_size_area(
    max_size = 26
  ) +

  scale_x_discrete(
```

```
      limits = application_order,
      labels = function(x) {
        str_wrap(
          x,
          width = 24
        )
      },
      drop = FALSE
    ) +

    scale_y_discrete(
      limits = rev(outcome_order),
      labels = function(x) {
        str_wrap(
          x,
          width = 32
        )
      },
      drop = FALSE
    ) +

    labs(
      x = "Application domain",
      y = "Outcome domain"
    ) +

    theme_bw(
      base_size = 12
    ) +

    theme(
      axis.text.x = element_text(
        angle = 45,
        hjust = 1,
        vjust = 1,
        color = "black"
      ),

      axis.text.y = element_text(
        size = 10,
        color = "black"
      ),

      axis.title.x = element_text(
        face = "bold",
        color = "black"
      ),

      axis.title.y = element_text(
        face = "bold",
        color = "black"
      ),

      panel.grid.major = element_line(
        linetype = "dotted",
        linewidth = 0.3
      ),

      panel.grid.minor = element_blank(),

      legend.position = "none"
    )


  # ------------------------------------------------------------
  # 11. Show plot
  # ------------------------------------------------------------

  print(p_bubble)

  invisible(list(
    p_bubble = p_bubble,
    plot_df = plot_df
  ))

}

# ==============================================================================
# SECTION: model_landscape
# ==============================================================================
analysis_model_landscape <- function(data_dir = "data", output_dir = "output") {

```

```r
if (!dir.exists(output_dir)) {
  dir.create(output_dir, recursive = TRUE)
}

# ============================================================
# Model landscape figure
# Sorted by descending number of studies
# Harmonized with the implementation-readiness figure style
# Dark blue tone: #31688E
# ============================================================


# -------------------------------------------------------------------------
# 1. Define file path
# -------------------------------------------------------------------------

resolved_file_path <- .review_input(data_dir, "Model_Landscape_Data.xlsx")

message("Excel file used: ", normalizePath(resolved_file_path))


# -------------------------------------------------------------------------
# 2. Read Excel file
# -------------------------------------------------------------------------

available_sheets <- excel_sheets(resolved_file_path)

if ("Model_Family_Summary" %in% available_sheets) {

  sheet_to_read <- "Model_Family_Summary"

} else {

  sheet_to_read <- available_sheets[1]

  warning(
    paste0(
      "Sheet 'Model_Family_Summary' was not found. ",
      "The first sheet is used instead: ",
      sheet_to_read
    )
  )
}

Model_Landscape_Data <- read_excel(
  path = resolved_file_path,
  sheet = sheet_to_read
)

# -------------------------------------------------------------------------
# 3. Check required columns
# -------------------------------------------------------------------------

required_columns <- c(
  "Model_Family",
  "n_studies",
  "Include_in_primary_plot"
)

missing_columns <- setdiff(
  required_columns,
  names(Model_Landscape_Data)
)

if (length(missing_columns) > 0) {

  stop(
    paste0(
      "The following required columns are missing from the workbook: ",
      paste(missing_columns, collapse = ", ")
    )
  )
}


# -------------------------------------------------------------------------
# 4. Prepare model-family data
# -------------------------------------------------------------------------

model_data <- Model_Landscape_Data %>%
  transmute(
    Model_Family = trimws(
```

```r
        as.character(Model_Family)
      ),

      n_studies = suppressWarnings(
        as.numeric(n_studies)
      ),

      Include_in_primary_plot = .review_as_logical(
        Include_in_primary_plot
      )
    ) %>%
    filter(
      !is.na(Model_Family),
      Model_Family != "",
      !is.na(n_studies),
      n_studies >= 0
    )


# ------------------------------------------------------------------------------
# 5. Primary plot data
# ------------------------------------------------------------------------------
# Only model families marked TRUE / WAHR in the Excel file.
#
# Sorting:
#   - descending number of studies
#   - alphabetical order in the case of equal frequencies

plot_data <- model_data %>%
  filter(
    Include_in_primary_plot
  ) %>%
  arrange(
    desc(n_studies),
    Model_Family
  ) %>%
  mutate(
    Model_Family = factor(
      Model_Family,
      levels = rev(
        unique(Model_Family)
      )
    )
  )

if (nrow(plot_data) == 0) {
  stop(
    paste0(
      "No model families remain for the primary plot after applying ",
      "Include_in_primary_plot."
    )
  )
}


# ------------------------------------------------------------------------------
# 6. Sensitivity plot data
# ------------------------------------------------------------------------------
# Includes all valid model categories, including categories marked FALSE,
# such as:
# - Bespoke / fine-tuned LLM systems
# - Unspecified ChatGPT

plot_data_sensitivity <- model_data %>%
  arrange(
    desc(n_studies),
    Model_Family
  ) %>%
  mutate(
    Model_Family = factor(
      Model_Family,
      levels = rev(
        unique(Model_Family)
      )
    )
  )


# ------------------------------------------------------------------------------
# 7. Display final datasets
# ------------------------------------------------------------------------------

```

```
message("\nPrimary plot data, sorted by descending frequency:")

print(
  plot_data %>%
    select(
      Model_Family,
      n_studies
    )
)

message("\nSensitivity plot data, sorted by descending frequency:")

print(
  plot_data_sensitivity %>%
    select(
      Model_Family,
      n_studies
    )
)


# ------------------------------------------------------------------------
# 8. Plot function
# ------------------------------------------------------------------------

make_model_landscape_plot <- function(data) {

  if (nrow(data) == 0) {
    stop(
      "The data set passed to the plotting function contains no rows."
    )
  }

  max_x <- max(
    data$n_studies,
    na.rm = TRUE
  )

  # Avoid errors if all values are zero
  if (!is.finite(max_x) || max_x <= 0) {
    max_x <- 1
  }

  label_offset <- max_x * 0.025
  x_axis_limit <- max_x * 1.16

  data_for_plot <- data %>%
    mutate(
      label = as.character(n_studies),
      label_x = n_studies + label_offset
    )

  ggplot(
    data = data_for_plot,
    aes(
      x = n_studies,
      y = Model_Family
    )
  ) +
    geom_col(
      width = 0.68,
      fill = "#31688E",
      alpha = 0.95
    ) +
    geom_text(
      aes(
        x = label_x,
        label = label
      ),
      hjust = 0,
      size = 3.5,
      colour = "grey20"
    ) +
    scale_x_continuous(
      limits = c(
        0,
        x_axis_limit
      ),
      breaks = pretty(
        c(0, max_x),
        n = 6
      ),
```

```
      expand = expansion(
        mult = c(0, 0.02)
      )
    ) +
    labs(
      x = "Number of studies",
      y = NULL
    ) +
    .review_theme_bar(
      base_size = 11
    )
}


# ---------------------------------------------------------------------------
# 9. Generate primary plot
# ---------------------------------------------------------------------------

fig_model_landscape <- make_model_landscape_plot(
  plot_data
)

print(fig_model_landscape)


# ---------------------------------------------------------------------------
# 10. Generate sensitivity plot
# ---------------------------------------------------------------------------

fig_model_landscape_sensitivity <- make_model_landscape_plot(
  plot_data_sensitivity
)

print(fig_model_landscape_sensitivity)


message(
  "\nFigures will be saved in: ",
  normalizePath(output_dir)
)


# ---------------------------------------------------------------------------
# 11. Export primary plot
# ---------------------------------------------------------------------------

ggsave(
  filename = file.path(
    output_dir,
    "Figure_Model_Landscape_primary.pdf"
  ),
  plot = fig_model_landscape,
  width = 180,
  height = 110,
  units = "mm",
  bg = "white"
)

ggsave(
  filename = file.path(
    output_dir,
    "Figure_Model_Landscape_primary.png"
  ),
  plot = fig_model_landscape,
  width = 180,
  height = 110,
  units = "mm",
  dpi = 600,
  bg = "white"
)

ggsave(
  filename = file.path(
    output_dir,
    "Figure_Model_Landscape_primary.tiff"
  ),
  plot = fig_model_landscape,
  width = 180,
  height = 110,
  units = "mm",
  dpi = 600,
  compression = "lzw",
```

```
    bg = "white"
  )


  # ------------------------------------------------------------------------
  # 12. Export sensitivity plot
  # ------------------------------------------------------------------------

  ggsave(
    filename = file.path(
      output_dir,
      "Figure_Model_Landscape_sensitivity.pdf"
    ),
    plot = fig_model_landscape_sensitivity,
    width = 180,
    height = 125,
    units = "mm",
    bg = "white"
  )

  ggsave(
    filename = file.path(
      output_dir,
      "Figure_Model_Landscape_sensitivity.png"
    ),
    plot = fig_model_landscape_sensitivity,
    width = 180,
    height = 125,
    units = "mm",
    dpi = 600,
    bg = "white"
  )

  ggsave(
    filename = file.path(
      output_dir,
      "Figure_Model_Landscape_sensitivity.tiff"
    ),
    plot = fig_model_landscape_sensitivity,
    width = 180,
    height = 125,
    units = "mm",
    dpi = 600,
    compression = "lzw",
    bg = "white"
  )


  # ------------------------------------------------------------------------
  # 13. Completion message
  # ------------------------------------------------------------------------

  message(
    paste0(
      "\nModel landscape figures created successfully.\n",
      "The model families are sorted by descending study frequency.\n",
      "Categories with equal frequencies are sorted alphabetically.\n",
      "Study counts are not mutually exclusive because individual studies ",
      "may evaluate more than one model family."
    )
  )

  invisible(list(
    fig_model_landscape = fig_model_landscape,
    fig_model_landscape_sensitivity = fig_model_landscape_sensitivity,
    plot_data = plot_data,
    plot_data_sensitivity = plot_data_sensitivity
  ))

}

# ==============================================================================
# SECTION: disease_landscape
# ==============================================================================
analysis_disease_landscape <- function(data_dir = "data", output_dir = "output") {

  if (!dir.exists(output_dir)) {
    dir.create(output_dir, recursive = TRUE)
  }

  # ================================================================
  # Disease landscape figure
```

```r
# Sorted by descending number of studies
# Harmonized with the model-landscape and implementation-readiness style
# Bar colour: #31688E
# ============================================================


# ---------------------------------------------------------------------------
# 1. Define file path
# ---------------------------------------------------------------------------

resolved_file_path <- .review_input(data_dir, "Disease_Landscape_Data.xlsx")

message("Excel file used: ", normalizePath(resolved_file_path))


# ---------------------------------------------------------------------------
# 2. Read Excel input
# ---------------------------------------------------------------------------

available_sheets <- excel_sheets(resolved_file_path)

if ("Disease_Summary_Primary" %in% available_sheets) {

  sheet_to_read <- "Disease_Summary_Primary"

} else {

  sheet_to_read <- available_sheets[1]

  warning(
    paste0(
      "Sheet 'Disease_Summary_Primary' was not found. ",
      "The first sheet is used instead: ",
      sheet_to_read
    )
  )
}

Disease_Landscape_Data <- read_excel(
  path = resolved_file_path,
  sheet = sheet_to_read
)

# ---------------------------------------------------------------------------
# 3. Clean column names
# ---------------------------------------------------------------------------

clean_names <- function(x) {
  x <- trimws(x)
  x <- gsub("\\s+", "_", x)
  x <- gsub("[^A-Za-z0-9_]", "", x)
  x
}

names(Disease_Landscape_Data) <- clean_names(
  names(Disease_Landscape_Data)
)


# ---------------------------------------------------------------------------
# 4. Check required columns
# ---------------------------------------------------------------------------

required_columns <- c(
  "Disease_Category",
  "n_studies",
  "Include_in_primary_plot"
)

missing_columns <- setdiff(
  required_columns,
  names(Disease_Landscape_Data)
)

if (length(missing_columns) > 0) {

  stop(
    paste0(
      "The following required columns are missing from the workbook: ",
      paste(missing_columns, collapse = ", ")
    )
  )
```

```
}


# ---------------------------------------------------------------------------
# 5. Prepare disease data
# ---------------------------------------------------------------------------

disease_data <- Disease_Landscape_Data %>%
  transmute(
    Disease_Category = trimws(
      as.character(Disease_Category)
    ),

    n_studies = suppressWarnings(
      as.numeric(n_studies)
    ),

    Plot_Order = if ("Plot_Order" %in% names(Disease_Landscape_Data)) {
      suppressWarnings(
        as.numeric(Plot_Order)
      )
    } else {
      NA_real_
    },

    Include_in_primary_plot = .review_as_logical(
      Include_in_primary_plot
    )
  ) %>%
  filter(
    !is.na(Disease_Category),
    Disease_Category != "",
    Disease_Category != "Summe",
    !is.na(n_studies),
    n_studies > 0
  )


# ---------------------------------------------------------------------------
# 6. Create missing tie-order values if necessary
# ---------------------------------------------------------------------------

disease_data <- disease_data %>%
  mutate(
    Plot_Order = ifelse(
      is.na(Plot_Order),
      row_number(),
      Plot_Order
    )
  )


# ---------------------------------------------------------------------------
# 7. Prepare primary plot data
# ---------------------------------------------------------------------------
# Sorting:
#   - descending number of studies
#   - Plot_Order for categories with the same frequency
#   - alphabetical order as final tie-breaker

plot_data <- disease_data %>%
  filter(
    Include_in_primary_plot
  ) %>%
  arrange(
    desc(n_studies),
    Plot_Order,
    Disease_Category
  ) %>%
  mutate(
    Disease_Category = factor(
      Disease_Category,
      levels = rev(
        unique(Disease_Category)
      )
    )
  )

if (nrow(plot_data) == 0) {
  stop(
    paste0(
      "No disease categories remain for the figure after applying ",
```

```
      "Include_in_primary_plot."
    )
  )
}

.review_assert_count(
  sum(plot_data$n_studies),
  REVIEW_N_STUDIES,
  "Disease-landscape assignments"
)


# --------------------------------------------------------------------------
# 8. Display final data
# --------------------------------------------------------------------------

message(
  "\nDisease landscape data, sorted by descending frequency:"
)

print(
  plot_data %>%
    select(
      Disease_Category,
      n_studies,
      Plot_Order
    )
)

message(
  "\nTotal number of included study assignments: ",
  sum(plot_data$n_studies, na.rm = TRUE)
)


# --------------------------------------------------------------------------
# 9. Plot function
# --------------------------------------------------------------------------

make_disease_landscape_plot <- function(data) {

  if (nrow(data) == 0) {
    stop(
      "The data set passed to the plotting function contains no rows."
    )
  }

  max_x <- max(
    data$n_studies,
    na.rm = TRUE
  )

  if (!is.finite(max_x) || max_x <= 0) {
    max_x <- 1
  }

  label_offset <- max_x * 0.025
  x_axis_limit <- max_x * 1.16

  data_for_plot <- data %>%
    mutate(
      label = as.character(n_studies),
      label_x = n_studies + label_offset
    )

  ggplot(
    data = data_for_plot,
    aes(
      x = n_studies,
      y = Disease_Category
    )
  ) +
    geom_col(
      width = 0.68,
      fill = "#31688E",
      alpha = 0.95
    ) +
    geom_text(
      aes(
        x = label_x,
        label = label
      ),
```

```
        hjust = 0,
        size = 3.5,
        colour = "grey20"
      ) +
      scale_x_continuous(
        limits = c(
          0,
          x_axis_limit
        ),
        breaks = pretty(
          c(0, max_x),
          n = 6
        ),
        expand = expansion(
          mult = c(0, 0.02)
        )
      ) +
      labs(
        x = "Number of studies",
        y = NULL
      ) +
      .review_theme_bar(
        base_size = 11
      )
}


# ---------------------------------------------------------------------------
# 10. Generate figure
# ---------------------------------------------------------------------------

fig_disease_landscape <- make_disease_landscape_plot(
  plot_data
)

print(fig_disease_landscape)


message(
  "\nFigures will be saved in: ",
  normalizePath(output_dir)
)


# ---------------------------------------------------------------------------
# 11. Export PDF
# ---------------------------------------------------------------------------

ggsave(
  filename = file.path(
    output_dir,
    "Figure_Disease_Landscape.pdf"
  ),
  plot = fig_disease_landscape,
  width = 180,
  height = 135,
  units = "mm",
  bg = "white"
)


# ---------------------------------------------------------------------------
# 12. Export PNG
# ---------------------------------------------------------------------------

ggsave(
  filename = file.path(
    output_dir,
    "Figure_Disease_Landscape.png"
  ),
  plot = fig_disease_landscape,
  width = 180,
  height = 135,
  units = "mm",
  dpi = 600,
  bg = "white"
)


# ---------------------------------------------------------------------------
# 13. Export TIFF
# ---------------------------------------------------------------------------
```

```r

  ggsave(
    filename = file.path(
      output_dir,
      "Figure_Disease_Landscape.tiff"
    ),
    plot = fig_disease_landscape,
    width = 180,
    height = 135,
    units = "mm",
    dpi = 600,
    compression = "lzw",
    bg = "white"
  )


  # ------------------------------------------------------------------------
  # 14. Completion message
  # ------------------------------------------------------------------------

  message(
    paste0(
      "\nDisease landscape figure created successfully.\n",
      "Disease categories are sorted by descending study frequency.\n",
      "Categories with identical frequencies follow the Excel Plot_Order.\n",
      "Blank rows and the summary row are excluded automatically.\n",
      "Total number of study assignments: ",
      sum(plot_data$n_studies, na.rm = TRUE),
      "."
    )
  )

  invisible(list(
    fig_disease_landscape = fig_disease_landscape,
    plot_data = plot_data
  ))

}

# ==============================================================================
# SECTION: implementation_readiness
# ==============================================================================
analysis_implementation_readiness <- function(data_dir = "data", output_dir = "output") {

  if (!dir.exists(output_dir)) {
    dir.create(output_dir, recursive = TRUE)
  }

  # Implementation-readiness indicators


  # ------------------------------------------------------------------------
  # 1. Load input
  # ------------------------------------------------------------------------

  input_path <- .review_input(data_dir, "Implementation_Readiness_Input.xlsx")

  Implementation_Readiness_Input <- read_excel(input_path)

  # Total number of included studies, read from the README sheet.
  readme_data <- read_excel(
    input_path,
    sheet = "README"
  )

  n_total <- readme_data %>%
    filter(field == "n_total") %>%
    pull(value) %>%
    as.numeric()

  # The denominator must be explicitly documented in the input workbook.
  if (length(n_total) != 1L || is.na(n_total)) {
    stop("README must contain exactly one numeric n_total value.", call. = FALSE)
  }

  .review_assert_count(
    n_total,
    REVIEW_N_STUDIES,
    "Implementation-readiness denominator"
  )

  # ------------------------------------------------------------------------
```

```
# 2. Define colour palette
# --------------------------------------------------------------------------

pyramid_palette_1 <- c(
  "Deep Navy"  = "#1F3B5B",
  "Petrol"     = "#264653",
  "Teal"       = "#2A9D8F",
  "Sage Green" = "#8AB17D",
  "Sand"       = "#E9C46A",
  "Terracotta" = "#E76F51"
)

# --------------------------------------------------------------------------
# 3. Prepare data
# --------------------------------------------------------------------------

indicators <- Implementation_Readiness_Input %>%
  mutate(
    indicator_order = as.numeric(indicator_order),
    n_studies = as.numeric(n_studies),
    percent = as.numeric(percent)
  ) %>%
  arrange(indicator_order) %>%
  mutate(
    indicator = factor(
      indicator,
      levels = rev(indicator)
    ),

    # Recycled so that more indicators than palette colours still get a fill.
    bar_color = rep(
      unname(pyramid_palette_1),
      length.out = n()
    ),

    label = paste0(
      n_studies,
      "/",
      n_total,
      " (",
      sprintf("%.1f", percent),
      "%)"
    ),

    label_x = percent + 2.5
  )

.review_validate_readiness(indicators, n_total)

# Upper x-axis limit: 80 %, extended in 20 % steps if an indicator exceeds it,
# so that coord_cartesian() cannot clip a bar or its label.
x_axis_max <- max(
  80,
  ceiling(max(indicators$percent, na.rm = TRUE) / 20) * 20
)

# --------------------------------------------------------------------------
# 4. Plot
# --------------------------------------------------------------------------

p_readiness <- ggplot(
  indicators,
  aes(
    x = percent,
    y = indicator,
    fill = bar_color
  )
) +
  geom_col(
    width = 0.68,
    alpha = 0.95
  ) +
  geom_text(
    aes(
      x = label_x,
      label = label
    ),
    hjust = 0,
    size = 3.5,
    color = "grey20"
  ) +
  scale_fill_identity() +
```

```
    scale_x_continuous(
      breaks = seq(0, x_axis_max, 20),
      labels = function(x) paste0(x, "%"),
      expand = expansion(mult = c(0, 0))
    ) +
    coord_cartesian(
      xlim = c(0, x_axis_max),
      clip = "off"
    ) +
    labs(
      x = "Studies meeting criterion (%)",
      y = NULL
    ) +
    theme_minimal(base_size = 11) +
    theme(
      legend.position = "none",

      panel.grid.major.y = element_blank(),
      panel.grid.minor = element_blank(),
      panel.grid.major.x = element_line(
        color = "grey85",
        linewidth = 0.35
      ),

      axis.text.y = element_text(
        size = 10,
        color = "grey15"
      ),
      axis.text.x = element_text(
        size = 9,
        color = "grey25"
      ),
      axis.title.x = element_text(
        size = 10,
        color = "grey20",
        margin = margin(t = 8)
      ),

      plot.background = element_rect(
        fill = "white",
        colour = NA
      ),
      panel.background = element_rect(
        fill = "white",
        colour = NA
      ),

      plot.margin = margin(8, 45, 8, 8)
    )

  # Display the figure
  print(p_readiness)

  invisible(list(
    p_readiness = p_readiness,
    indicators = indicators
  ))

}

# ============================================================================
# SECTION: rob_domains
# ============================================================================
analysis_rob_domains <- function(data_dir = "data", output_dir = "output",
                                 rob_file = NULL, rob_sheet = NULL) {

  if (!dir.exists(output_dir)) {
    dir.create(output_dir, recursive = TRUE)
  }

  # ============================================================
  # AQAT:RoB domain-level risk of bias
  # 100% stacked bar chart
  #
  # Included studies: Tool 1 == "AQAT"
  # No Overall RoB row
  # No count labels inside bars
  # ============================================================


  # ------------------------------------------------------------
  # 1. Load the risk-of-bias workbook
```

```
# ------------------------------------------------------------

rob_raw <- .review_read_rob(data_dir, rob_file, rob_sheet)

# Harmonise column names:
# Example: "Tool 1" becomes "Tool_1"
rob_raw <- rob_raw %>%
  rename_with(
    ~ str_replace_all(
      str_trim(.x),
      "\\s+",
      "_"
    )
  )

# ------------------------------------------------------------
# 2. Define and check required columns
# ------------------------------------------------------------

domain_cols <- paste0("Domain_", 1:9)

required_cols <- c(
  "Study_ID",
  "Tool_1",
  domain_cols
)

missing_cols <- setdiff(
  required_cols,
  names(rob_raw)
)

if (length(missing_cols) > 0) {
  stop(
    paste0(
      "The following required columns were not found: ",
      paste(missing_cols, collapse = ", "),
      ". Please check the column names in the Excel file."
    )
  )
}

# ------------------------------------------------------------
# 3. Nature-style RoB colour palette
# ------------------------------------------------------------

rob_cols_nature <- c(
  "Low"     = "#4C956C",
  "Unclear" = "#E9C46A",
  "High"    = "#D95F5F"
)

text_col <- "#2E2E2E"
grid_col <- "#D9D9D9"

# ------------------------------------------------------------
# 4. Define accepted RoB labels
# ------------------------------------------------------------

unclear_values <- c(
  "unclear",
  "not assessable",
  "unclear / not assessable",
  "unclear/not assessable"
)

# ------------------------------------------------------------
# 5. Clean data and include only AQAT-assessed studies
# ------------------------------------------------------------

rob_clean <- rob_raw %>%
  mutate(
    Tool_1 = str_to_lower(
      str_squish(
        as.character(Tool_1)
      )
    ),
    Study_ID = as.character(Study_ID),
    across(
      all_of(domain_cols),
      ~ str_to_lower(
        str_squish(
```

```
          as.character(.x)
        )
      )
    )
  ) %>%
  filter(.review_is_aqat_rob(Tool_1))

n_included <- nrow(rob_clean)

message(
  "Number of AQAT-assessed studies included: ",
  n_included
)

.review_assert_count(
  n_included,
  REVIEW_N_AQAT_ROB,
  "AQAT:RoB domain analysis"
)

# ------------------------------------------------------------
# 6. Convert data to long format
# ------------------------------------------------------------

rob_domain_long <- rob_clean %>%
  select(
    Study_ID,
    all_of(domain_cols)
  ) %>%
  pivot_longer(
    cols = all_of(domain_cols),
    names_to = "Domain",
    values_to = "Risk"
  ) %>%
  mutate(

    # Rename AQAT domains
    Domain = recode(
      Domain,
      "Domain_1" = "Question Source",
      "Domain_2" = "Question Selection",
      "Domain_3" = "Question Manipulation",
      "Domain_4" = "Reference Answer Source",
      "Domain_5" = "Reference Answer Selection",
      "Domain_6" = "LLM Answer Selection",
      "Domain_7" = "Evaluator Selection",
      "Domain_8" = "Blinding of Evaluators",
      "Domain_9" = "Performance Metrics"
    ),

    # Harmonise RoB judgements
    Risk = case_when(
      Risk == "low" ~ "Low",
      Risk == "high" ~ "High",
      Risk %in% unclear_values ~ "Unclear",
      is.na(Risk) ~ "Unclear",
      TRUE ~ "Unclear"
    ),

    # Define RoB order
    Risk = factor(
      Risk,
      levels = c(
        "Low",
        "Unclear",
        "High"
      )
    ),

    # Define domain order
    Domain = factor(
      Domain,
      levels = rev(c(
        "Question Source",
        "Question Selection",
        "Question Manipulation",
        "Reference Answer Source",
        "Reference Answer Selection",
        "LLM Answer Selection",
        "Evaluator Selection",
        "Blinding of Evaluators",
        "Performance Metrics"
```

```
      ))
    )
  )

# ------------------------------------------------------------
# 7. Summarise counts and percentages
# ------------------------------------------------------------

risk_levels <- factor(
  c(
    "Low",
    "Unclear",
    "High"
  ),
  levels = c(
    "Low",
    "Unclear",
    "High"
  )
)

rob_domain_summary <- rob_domain_long %>%
  count(
    Domain,
    Risk,
    name = "n"
  ) %>%
  complete(
    Domain,
    Risk = risk_levels,
    fill = list(n = 0)
  ) %>%
  group_by(Domain) %>%
  mutate(
    total = sum(n),
    pct = n / total
  ) %>%
  ungroup()

print(rob_domain_summary)

# Optional check:
# Each AQAT domain should include the same number of studies
rob_domain_summary %>%
  group_by(Domain) %>%
  summarise(
    total_studies = sum(n),
    .groups = "drop"
  ) %>%
  print()

# ------------------------------------------------------------
# 8. Create the figure
# ------------------------------------------------------------

p_rob_domains <- ggplot(
  rob_domain_summary,
  aes(
    y = Domain,
    x = pct,
    fill = Risk
  )
) +
  geom_col(
    width = 0.70,
    colour = "white",
    linewidth = 0.45
  ) +
  scale_x_continuous(
    labels = percent_format(
      accuracy = 1
    ),
    limits = c(0, 1),
    breaks = seq(
      0,
      1,
      by = 0.2
    ),
    expand = expansion(
      mult = c(0, 0)
    )
  ) +
```

```
    scale_fill_manual(
      values = rob_cols_nature,
      breaks = c(
        "Low",
        "Unclear",
        "High"
      ),
      drop = FALSE
    ) +
    labs(
      x = "Studies (%)",
      y = NULL,
      fill = NULL
    ) +
    theme_minimal(
      base_size = 14
    ) +
    theme(
      text = element_text(
        colour = text_col
      ),
      axis.text = element_text(
        colour = text_col
      ),
      axis.text.y = element_text(
        size = 12
      ),
      axis.title.x = element_text(
        size = 14,
        margin = margin(
          t = 8
        )
      ),
      panel.grid.major.y = element_blank(),
      panel.grid.minor = element_blank(),
      panel.grid.major.x = element_line(
        colour = grid_col,
        linetype = "dotted",
        linewidth = 0.4
      ),
      legend.position = "bottom",
      legend.text = element_text(
        size = 13
      ),
      plot.margin = margin(
        t = 10,
        r = 20,
        b = 10,
        l = 10
      )
    )

  # Display the figure
  print(p_rob_domains)

  invisible(list(
    p_rob_domains = p_rob_domains,
    rob_domain_summary = rob_domain_summary
  ))

}

# ==============================================================================
# SECTION: rob_overall
# ==============================================================================
analysis_rob_overall <- function(data_dir = "data", output_dir = "output",
                                 rob_file = NULL, rob_sheet = NULL) {

  if (!dir.exists(output_dir)) {
    dir.create(output_dir, recursive = TRUE)
  }

  # ============================================================
  # Overall AQAT:RoB risk of bias
  # ============================================================


  # ------------------------------------------------------------
  # 1. Load the risk-of-bias workbook
  # ------------------------------------------------------------

  rob_raw <- .review_read_rob(data_dir, rob_file, rob_sheet)
```

```

# Harmonise column names:
# Example: "Tool 1" becomes "Tool_1"
rob_raw <- rob_raw %>%
  rename_with(
    ~ str_replace_all(
      str_trim(.x),
      "\\s+",
      "_"
    )
  )

# ----------------------------------------------------------
# 2. Define and check required columns
# ----------------------------------------------------------

domain_cols <- paste0("Domain_", 1:9)

required_cols <- c(
  "Tool_1",
  domain_cols
)

missing_cols <- setdiff(
  required_cols,
  names(rob_raw)
)

if (length(missing_cols) > 0) {
  stop(
    paste0(
      "The following required columns were not found: ",
      paste(missing_cols, collapse = ", "),
      ". Please check the column names in the Excel file."
    )
  )
}

# ----------------------------------------------------------
# 3. Nature-style RoB colour palette
# ----------------------------------------------------------

rob_cols_nature <- c(
  "Low"     = "#4C956C",
  "Unclear" = "#E9C46A",
  "High"    = "#D95F5F"
)

text_col <- "#2E2E2E"
grid_col <- "#D9D9D9"

# ----------------------------------------------------------
# 4. Define accepted RoB labels
# ----------------------------------------------------------

unclear_values <- c(
  "unclear",
  "not assessable",
  "unclear / not assessable",
  "unclear/not assessable"
)

# ----------------------------------------------------------
# 5. Clean data and include only AQAT-assessed studies
# ----------------------------------------------------------

rob_clean <- rob_raw %>%
  mutate(
    Tool_1 = str_to_lower(
      str_squish(
        as.character(Tool_1)
      )
    ),
    across(
      all_of(domain_cols),
      ~ str_to_lower(
        str_squish(
          as.character(.x)
        )
      )
    )
  ) %>%
```

```
    filter(.review_is_aqat_rob(Tool_1))

  n_included <- nrow(rob_clean)

  message(
    "Number of AQAT-assessed studies included: ",
    n_included
  )

  .review_assert_count(
    n_included,
    REVIEW_N_AQAT_ROB,
    "Overall AQAT:RoB analysis"
  )

  # ----------------------------------------------------------
  # 6. Recalculate Overall RoB using the worst-domain rule
  # ----------------------------------------------------------
  # High:
  # At least one AQAT domain is rated high.
  #
  # Unclear:
  # No domain is high, but at least one domain is unclear,
  # not assessable, missing, or contains an unexpected value.
  #
  # Low:
  # All nine AQAT domains are rated low.

  rob_clean <- rob_clean %>%
    rowwise() %>%
    mutate(
      Overall_RoB = case_when(

        # At least one high-risk domain
        any(
          c_across(all_of(domain_cols)) == "high",
          na.rm = TRUE
        ) ~ "High",

        # At least one missing domain
        any(
          is.na(c_across(all_of(domain_cols)))
        ) ~ "Unclear",

        # At least one unclear or not-assessable domain
        any(
          c_across(all_of(domain_cols)) %in% unclear_values,
          na.rm = TRUE
        ) ~ "Unclear",

        # All domains are low
        all(
          c_across(all_of(domain_cols)) == "low"
        ) ~ "Low",

        # Any unexpected entry is classified conservatively
        TRUE ~ "Unclear"
      )
    ) %>%
    ungroup()

  # ----------------------------------------------------------
  # 7. Summarise overall RoB counts and percentages
  # ----------------------------------------------------------

  risk_levels <- tibble(
    Overall_RoB = c(
      "Low",
      "Unclear",
      "High"
    )
  )

  rob_overall_summary <- rob_clean %>%
    count(
      Overall_RoB,
      name = "n"
    ) %>%
    right_join(
      risk_levels,
      by = "Overall_RoB"
    ) %>%
```

```r
    mutate(
      n = replace_na(n, 0),
      Overall_RoB = factor(
        Overall_RoB,
        levels = c(
          "Low",
          "Unclear",
          "High"
        )
      )
    ) %>%
    arrange(Overall_RoB) %>%
    mutate(
      total = sum(n),
      pct = n / total,
      label = paste0(
        n,
        "/",
        total,
        " (",
        percent(
          pct,
          accuracy = 0.1
        ),
        ")"
      )
    )

  print(rob_overall_summary)

  # ------------------------------------------------------------
  # 8. Create the figure
  # ------------------------------------------------------------

  p_rob_overall <- ggplot(
    rob_overall_summary,
    aes(
      x = Overall_RoB,
      y = n,
      fill = Overall_RoB
    )
  ) +
    geom_col(
      width = 0.62,
      colour = "white",
      linewidth = 0.45
    ) +
    geom_text(
      aes(label = label),
      vjust = -0.45,
      size = 4.4,
      colour = text_col
    ) +
    scale_fill_manual(
      values = rob_cols_nature,
      breaks = c(
        "Low",
        "Unclear",
        "High"
      ),
      drop = FALSE
    ) +
    scale_y_continuous(
      limits = c(
        0,
        max(rob_overall_summary$n) * 1.18
      ),
      breaks = pretty_breaks(n = 6),
      expand = expansion(
        mult = c(0, 0)
      )
    ) +
    labs(
      title = "Overall Risk of Bias",
      caption = paste0(
        "Overall risk was classified by the ",
        "highest-risk domain per study."
      ),
      x = NULL,
      y = "Number of studies",
      fill = NULL
    ) +
```

```
    theme_minimal(
      base_size = 14
    ) +
    theme(
      text = element_text(
        colour = text_col
      ),
      plot.title = element_text(
        size = 16,
        face = "bold",
        hjust = 0.5,
        margin = margin(
          b = 10
        )
      ),
      plot.caption = element_text(
        size = 11,
        face = "italic",
        colour = text_col,
        hjust = 0.5,
        margin = margin(
          t = 12
        )
      ),
      axis.text = element_text(
        colour = text_col
      ),
      axis.text.x = element_text(
        size = 13
      ),
      axis.title.y = element_text(
        size = 14,
        margin = margin(
          r = 8
        )
      ),
      panel.grid.major.x = element_blank(),
      panel.grid.minor = element_blank(),
      panel.grid.major.y = element_line(
        colour = grid_col,
        linewidth = 0.4
      ),
      legend.position = "none",
      plot.margin = margin(
        t = 10,
        r = 20,
        b = 10,
        l = 10
      )
    )

  # Display figure
  print(p_rob_overall)

  invisible(list(
    p_rob_overall = p_rob_overall,
    rob_overall_summary = rob_overall_summary
  ))

}

# ============================================================================
# SECTION: rob_combined
# ============================================================================
analysis_rob_combined <- function(data_dir = "data", output_dir = "output",
                                  rob_file = NULL, rob_sheet = NULL) {

  if (!dir.exists(output_dir)) {
    dir.create(output_dir, recursive = TRUE)
  }

  # ============================================================
  # Combined figure: AQAT:RoB domain-level results and overall results
  # a = Domain-level risk of bias
  # b = Overall risk of bias
  # ============================================================


  # ------------------------------------------------------------
  # 1. Load the risk-of-bias workbook
  # ------------------------------------------------------------

```

```r
rob_raw <- .review_read_rob(data_dir, rob_file, rob_sheet)

# Harmonise column names
rob_raw <- rob_raw %>%
  rename_with(
    ~ str_replace_all(
      str_trim(.x),
      "\\s+",
      "_"
    )
  )

# ------------------------------------------------------------
# 2. Define and check required columns
# ------------------------------------------------------------

domain_cols <- paste0("Domain_", 1:9)

required_cols <- c(
  "Study_ID",
  "Tool_1",
  domain_cols
)

missing_cols <- setdiff(
  required_cols,
  names(rob_raw)
)

if (length(missing_cols) > 0) {
  stop(
    paste0(
      "The following required columns were not found: ",
      paste(missing_cols, collapse = ", "),
      ". Please check the column names in the Excel file."
    )
  )
}

# ------------------------------------------------------------
# 3. Define palette and labels
# ------------------------------------------------------------

rob_cols_nature <- c(
  "Low"     = "#4C956C",
  "Unclear" = "#E9C46A",
  "High"    = "#D95F5F"
)

text_col <- "#2E2E2E"
grid_col <- "#D9D9D9"

unclear_values <- c(
  "unclear",
  "not assessable",
  "unclear / not assessable",
  "unclear/not assessable"
)

risk_levels <- c(
  "Low",
  "Unclear",
  "High"
)

domain_names <- c(
  "Question Source",
  "Question Selection",
  "Question Manipulation",
  "Reference Answer Source",
  "Reference Answer Selection",
  "LLM Answer Selection",
  "Evaluator Selection",
  "Blinding of Evaluators",
  "Performance Metrics"
)

# ------------------------------------------------------------
# 4. Clean data and include only AQAT-assessed studies
# ------------------------------------------------------------

rob_clean <- rob_raw %>%
```

```
    mutate(
      Tool_1 = str_to_lower(
        str_squish(
          as.character(Tool_1)
        )
      ),
      Study_ID = as.character(Study_ID),
      across(
        all_of(domain_cols),
        ~ str_to_lower(
          str_squish(
            as.character(.x)
          )
        )
      )
    ) %>%
    filter(.review_is_aqat_rob(Tool_1))

  n_included <- nrow(rob_clean)

  message(
    "Number of AQAT-assessed studies included: ",
    n_included
  )

  .review_assert_count(
    n_included,
    REVIEW_N_AQAT_ROB,
    "Combined AQAT:RoB analysis"
  )

  # ---------------------------------------------------------------
  # 5. Prepare data for panel a
  # ---------------------------------------------------------------

  rob_domain_long <- rob_clean %>%
    select(
      Study_ID,
      all_of(domain_cols)
    ) %>%
    pivot_longer(
      cols = all_of(domain_cols),
      names_to = "Domain",
      values_to = "Risk"
    ) %>%
    mutate(
      Domain = recode(
        Domain,
        "Domain_1" = "Question Source",
        "Domain_2" = "Question Selection",
        "Domain_3" = "Question Manipulation",
        "Domain_4" = "Reference Answer Source",
        "Domain_5" = "Reference Answer Selection",
        "Domain_6" = "LLM Answer Selection",
        "Domain_7" = "Evaluator Selection",
        "Domain_8" = "Blinding of Evaluators",
        "Domain_9" = "Performance Metrics"
      ),
      Risk = case_when(
        Risk == "low" ~ "Low",
        Risk == "high" ~ "High",
        Risk %in% unclear_values ~ "Unclear",
        is.na(Risk) ~ "Unclear",
        TRUE ~ "Unclear"
      ),
      Risk = factor(
        Risk,
        levels = risk_levels
      ),
      Domain = factor(
        Domain,
        levels = rev(domain_names)
      )
    )

  rob_domain_summary <- rob_domain_long %>%
    count(
      Domain,
      Risk,
      name = "n"
    ) %>%
    complete(
```

```
    Domain,
    Risk = factor(
      risk_levels,
      levels = risk_levels
    ),
    fill = list(n = 0)
  ) %>%
  group_by(Domain) %>%
  mutate(
    total = sum(n),
    pct = n / total
  ) %>%
  ungroup()

print(rob_domain_summary)

# ------------------------------------------------------------
# 6. Create panel a
# ------------------------------------------------------------

p_a <- ggplot(
  rob_domain_summary,
  aes(
    y = Domain,
    x = pct,
    fill = Risk
  )
) +
  geom_col(
    width = 0.70,
    colour = "white",
    linewidth = 0.45
  ) +
  scale_x_continuous(
    labels = percent_format(
      accuracy = 1
    ),
    limits = c(0, 1),
    breaks = seq(
      0,
      1,
      by = 0.2
    ),
    expand = expansion(
      mult = c(0, 0)
    )
  ) +
  scale_fill_manual(
    values = rob_cols_nature,
    breaks = risk_levels,
    drop = FALSE
  ) +
  labs(
    x = "Studies (%)",
    y = NULL,
    fill = NULL
  ) +
  theme_minimal(
    base_size = 14
  ) +
  theme(
    text = element_text(
      colour = text_col
    ),
    plot.title = element_blank(),
    axis.text = element_text(
      colour = text_col
    ),
    axis.text.y = element_text(
      size = 12
    ),
    axis.title.x = element_text(
      size = 14,
      margin = margin(t = 8)
    ),
    panel.grid.major.y = element_blank(),
    panel.grid.minor = element_blank(),
    panel.grid.major.x = element_line(
      colour = grid_col,
      linetype = "dotted",
      linewidth = 0.4
    ),
```

```
      legend.position = "bottom",
      legend.text = element_text(
        size = 12,
        colour = text_col
      ),
      plot.margin = margin(
        t = 10,
        r = 10,
        b = 10,
        l = 10
      )
    )

# ------------------------------------------------------------
# 7. Prepare data for panel b
# ------------------------------------------------------------

rob_overall <- rob_clean %>%
  rowwise() %>%
  mutate(
    Overall_RoB = case_when(
      any(
        c_across(all_of(domain_cols)) == "high",
        na.rm = TRUE
      ) ~ "High",
      any(
        is.na(c_across(all_of(domain_cols)))
      ) ~ "Unclear",
      any(
        c_across(all_of(domain_cols)) %in% unclear_values,
        na.rm = TRUE
      ) ~ "Unclear",
      all(
        c_across(all_of(domain_cols)) == "low"
      ) ~ "Low",
      TRUE ~ "Unclear"
    )
  ) %>%
  ungroup()

rob_overall_summary <- rob_overall %>%
  count(
    Overall_RoB,
    name = "n"
  ) %>%
  right_join(
    tibble(
      Overall_RoB = risk_levels
    ),
    by = "Overall_RoB"
  ) %>%
  mutate(
    n = replace_na(n, 0),
    Overall_RoB = factor(
      Overall_RoB,
      levels = risk_levels
    )
  ) %>%
  arrange(Overall_RoB) %>%
  mutate(
    total = sum(n),
    pct = n / total,
    label = paste0(
      n,
      "/",
      total,
      " (",
      percent(
        pct,
        accuracy = 0.1
      ),
      ")"
    )
  )

print(rob_overall_summary)

# ------------------------------------------------------------
# 8. Create panel b
# ------------------------------------------------------------

p_b <- ggplot(
```

```
    rob_overall_summary,
    aes(
      x = Overall_RoB,
      y = n,
      fill = Overall_RoB
    )
  ) +
    geom_col(
      width = 0.62,
      colour = "white",
      linewidth = 0.45
    ) +
    geom_text(
      aes(label = label),
      vjust = -0.45,
      size = 4.0,
      colour = text_col
    ) +
    scale_fill_manual(
      values = rob_cols_nature,
      breaks = risk_levels,
      drop = FALSE
    ) +
    scale_y_continuous(
      limits = c(
        0,
        max(rob_overall_summary$n) * 1.18
      ),
      breaks = pretty_breaks(n = 6),
      expand = expansion(
        mult = c(0, 0)
      )
    ) +
    labs(
      x = NULL,
      y = "Number of studies",
      fill = NULL
    ) +
    theme_minimal(
      base_size = 14
    ) +
    theme(
      text = element_text(
        colour = text_col
      ),
      plot.title = element_blank(),
      plot.caption = element_blank(),
      axis.text = element_text(
        colour = text_col
      ),
      axis.text.x = element_text(
        size = 12
      ),
      axis.title.y = element_text(
        size = 13,
        margin = margin(r = 8)
      ),
      panel.grid.major.x = element_blank(),
      panel.grid.minor = element_blank(),
      panel.grid.major.y = element_line(
        colour = grid_col,
        linewidth = 0.4
      ),
      legend.position = "none",
      plot.margin = margin(
        t = 10,
        r = 10,
        b = 10,
        l = 10
      )
    )

  # ------------------------------------------------------------
  # 9. Combine panels
  # ------------------------------------------------------------

  p_combined <- (
    p_a | p_b
  ) +
    plot_layout(
      widths = c(2.25, 1),
      guides = "collect"
```

```
    ) +
    plot_annotation(
      tag_levels = "a",
      tag_prefix = "",
      tag_suffix = "",
      theme = theme(
        plot.tag = element_text(
          size = 15,
          face = "bold",
          colour = text_col
        )
      )
    ) &
    theme(
      plot.title = element_blank(),
      plot.caption = element_blank(),
      plot.tag = element_text(
        size = 15,
        face = "bold",
        colour = text_col
      ),
      plot.tag.position = c(0, 1),

      legend.position = "bottom",
      legend.justification = "center",
      legend.box.just = "center",
      legend.box = "horizontal",
      legend.direction = "horizontal",

      legend.title = element_blank(),
      legend.text = element_text(
        size = 12,
        colour = text_col
      )
    )

  # ------------------------------------------------------------
  # 10. Display combined figure
  # ------------------------------------------------------------

  print(p_combined)

  invisible(list(
    p_combined = p_combined,
    rob_domain_summary = rob_domain_summary,
    rob_overall_summary = rob_overall_summary
  ))

}

# ==============================================================================
# SECTION: rob_heatmap
# ==============================================================================
analysis_rob_heatmap <- function(data_dir = "data", output_dir = "output",
                                 rob_file = NULL, rob_sheet = NULL,
                                 heatmap_file = NULL, heatmap_sheet = NULL) {

  if (!dir.exists(output_dir)) {
    dir.create(output_dir, recursive = TRUE)
  }

  # ============================================================
  # AQAT:RoB heatmap per study and domain
  # Output: Figure_RoB_heatmap_per_study_domain.png / .pdf
  # Overall risk is derived by the worst-domain rule, as elsewhere in this file.
  # ============================================================


  # ------------------------------------------------------------
  # 1. Load the risk-of-bias workbook
  # ------------------------------------------------------------

  if (is.null(heatmap_file)) heatmap_file <- rob_file
  if (is.null(heatmap_sheet)) heatmap_sheet <- rob_sheet

  rob_raw <- .review_read_rob(data_dir, heatmap_file, heatmap_sheet)

  # Harmonise column names, e.g. "Tool 1" -> "Tool_1"
  rob_raw <- rob_raw %>%
    rename_with(~ str_replace_all(str_trim(.x), "\\s+", "_"))

  # ------------------------------------------------------------
```

```
# 2. Define AQAT domain columns
# ------------------------------------------------------------

domain_cols <- paste0("Domain_", 1:9)

if (!all(domain_cols %in% names(rob_raw))) {
  stop("Domain_1 to Domain_9 were not found. Please check the column ",
       "names in the loaded Excel file.")
}

if (!"Tool_1" %in% names(rob_raw)) {
  stop("Column 'Tool_1' was not found. Please check whether the Excel ",
       "column is named 'Tool 1'.")
}

if (!"Study_ID" %in% names(rob_raw)) {
  stop("Column 'Study_ID' was not found. Please check the study identifier column.")
}

# ------------------------------------------------------------
# 3. Nature-style RoB colour palette
# ------------------------------------------------------------

rob_cols_nature <- c(
  "Low"     = "#4C956C",
  "Unclear" = "#E9C46A",
  "High"    = "#D95F5F"
)

text_col <- "#2E2E2E"
grid_col <- "#FFFFFF"

# ------------------------------------------------------------
# 4. Include only studies where AQAT was applied
# ------------------------------------------------------------

rob_clean <- rob_raw %>%
  mutate(
    Tool_1 = str_to_lower(str_squish(as.character(Tool_1))),
    Study_ID = as.character(Study_ID),
    across(
      all_of(domain_cols),
      ~ str_to_lower(str_squish(as.character(.x)))
    )
  ) %>%
  filter(.review_is_aqat_rob(Tool_1))

n_included <- nrow(rob_clean)

message(
  "Number of AQAT:RoB-assessed studies included in heatmap: ",
  n_included
)

.review_assert_count(
  n_included,
  REVIEW_N_AQAT_ROB,
  "AQAT:RoB heatmap"
)

# ------------------------------------------------------------
# 5. Recalculate Overall RoB for optional ordering
# ------------------------------------------------------------

rob_clean <- rob_clean %>%
  rowwise() %>%
  mutate(
    Overall_RoB = case_when(
      any(c_across(all_of(domain_cols)) == "high", na.rm = TRUE) ~ "High",
      any(is.na(c_across(all_of(domain_cols)))) ~ "Unclear",
      any(
        c_across(all_of(domain_cols)) %in%
          c(
            "unclear",
            "not assessable",
            "unclear / not assessable",
            "unclear/not assessable"
          ),
        na.rm = TRUE
      ) ~ "Unclear",
      all(c_across(all_of(domain_cols)) == "low") ~ "Low",
      TRUE ~ "Unclear"
```

```
    ),
    Study_Number = as.numeric(str_extract(Study_ID, "\\d+"))
  ) %>%
  ungroup()

# ------------------------------------------------------------
# 6. Choose study order
# ------------------------------------------------------------
# Option A: order studies by Study_ID ascending
# This is usually best for manuscript consistency.

study_order <- rob_clean %>%
  arrange(Study_Number) %>%
  pull(Study_ID)

# Option B: alternatively order by overall RoB, then Study_ID.
# Uncomment this block if you prefer risk-grouped ordering.
#
# study_order <- rob_clean %>%
#   mutate(
#     Overall_RoB = factor(
#       Overall_RoB,
#       levels = c("High", "Unclear", "Low")
#     )
#   ) %>%
#   arrange(Overall_RoB, Study_Number) %>%
#   pull(Study_ID)

# ------------------------------------------------------------
# 7. Prepare heatmap data
# ------------------------------------------------------------

rob_heatmap_long <- rob_clean %>%
  select(Study_ID, all_of(domain_cols), Overall_RoB) %>%
  pivot_longer(
    cols = all_of(domain_cols),
    names_to = "Domain",
    values_to = "Risk"
  ) %>%
  mutate(
    Domain = recode(
      Domain,
      "Domain_1" = "Question Source",
      "Domain_2" = "Question Selection",
      "Domain_3" = "Question Manipulation",
      "Domain_4" = "Reference Answer Source",
      "Domain_5" = "Reference Answer Selection",
      "Domain_6" = "LLM Answer Selection",
      "Domain_7" = "Evaluator Selection",
      "Domain_8" = "Blinding of Evaluators",
      "Domain_9" = "Performance Metrics"
    ),
    Risk = case_when(
      Risk == "low" ~ "Low",
      Risk == "high" ~ "High",
      Risk %in% c(
        "unclear",
        "not assessable",
        "unclear / not assessable",
        "unclear/not assessable"
      ) ~ "Unclear",
      TRUE ~ "Unclear"
    ),
    Risk = factor(
      Risk,
      levels = c("Low", "Unclear", "High")
    ),
    Domain = factor(
      Domain,
      levels = c(
        "Question Source",
        "Question Selection",
        "Question Manipulation",
        "Reference Answer Source",
        "Reference Answer Selection",
        "LLM Answer Selection",
        "Evaluator Selection",
        "Blinding of Evaluators",
        "Performance Metrics"
      )
    ),
    Study_ID = factor(
```

```
      Study_ID,
      levels = rev(study_order)
    )
  )

# ------------------------------------------------------------
# 8. Create heatmap
# ------------------------------------------------------------

p_rob_heatmap <- ggplot(
  rob_heatmap_long,
  aes(
    x = Domain,
    y = Study_ID,
    fill = Risk
  )
) +
  geom_tile(
    colour = grid_col,
    linewidth = 0.35,
    width = 0.95,
    height = 0.95
  ) +
  scale_fill_manual(
    values = rob_cols_nature,
    breaks = c("Low", "Unclear", "High"),
    drop = FALSE
  ) +
  labs(
    title = "Risk of Bias by Study and AQAT:RoB Domain",
    x = NULL,
    y = "Study",
    fill = NULL
  ) +
  theme_minimal(base_size = 13) +
  theme(
    text = element_text(colour = text_col),
    plot.title = element_text(
      size = 16,
      face = "bold",
      hjust = 0.5,
      margin = margin(b = 12)
    ),
    axis.text.x = element_text(
      size = 11,
      angle = 45,
      hjust = 1,
      vjust = 1,
      colour = text_col
    ),
    axis.text.y = element_text(
      size = 8.5,
      colour = text_col
    ),
    axis.title.y = element_text(
      size = 13,
      margin = margin(r = 8)
    ),
    panel.grid = element_blank(),
    legend.position = "bottom",
    legend.text = element_text(size = 12),
    plot.margin = margin(10, 20, 10, 10)
  )

print(p_rob_heatmap)
# ------------------------------------------------------------
# 9. Export heatmap
# ------------------------------------------------------------

ggsave(
  filename = file.path(output_dir, "Figure_RoB_heatmap_per_study_domain.png"),
  plot = p_rob_heatmap,
  width = 10.8,
  height = 13.5,
  dpi = 600
)

ggsave(
  filename = file.path(output_dir, "Figure_RoB_heatmap_per_study_domain.pdf"),
  plot = p_rob_heatmap,
  width = 10.8,
  height = 13.5
```

```
  )

  invisible(list(
    p_rob_heatmap = p_rob_heatmap,
    rob_heatmap_long = rob_heatmap_long
  ))

}

# ==============================================================================
# SECTION: maturity_rob
# ==============================================================================
analysis_maturity_rob <- function(data_dir = "data", output_dir = "output") {

  if (!dir.exists(output_dir)) {
    dir.create(output_dir, recursive = TRUE)
  }

  # ============================================================
  # Figure: Risk of Bias by Translational Maturity
  # ============================================================
  Maturity_RoB_Input <- read_excel(.review_input(data_dir, "Maturity_RoB_Input.xlsx"))
  # ------------------------------------------------------------
  # 1. Load the risk-of-bias workbook
  # ------------------------------------------------------------
  mat_raw <- Maturity_RoB_Input
  # Harmonise column names
  mat_raw <- mat_raw %>%
    rename_with(
      ~ str_replace_all(
        str_trim(.x),
        "\\s+",
        "_"
      )
    )
  # ------------------------------------------------------------
  # 2. Define and check required columns
  # ------------------------------------------------------------
  required_cols <- c(
    "level_order",
    "maturity_level",
    "rob_overall",
    "n_studies"
  )
  missing_cols <- setdiff(
    required_cols,
    names(mat_raw)
  )
  if (length(missing_cols) > 0) {
    stop(
      paste0(
        "The following required columns were not found: ",
        paste(missing_cols, collapse = ", "),
        ". Please check the column names in the Excel file."
      )
    )
  }
  # ------------------------------------------------------------
  # 3. Nature-style RoB colour palette
  # ------------------------------------------------------------
  rob_cols_nature <- c(
    "Low"            = "#4C956C",
    "Unclear"        = "#E9C46A",
    "High"           = "#D95F5F",
    "Not assessable" = "#ABABAB"
  )
  # Label colour per fill, for readability inside the bars
  rob_label_cols <- c(
    "Low"            = "white",
    "Unclear"        = "#2E2E2E",
    "High"           = "white",
    "Not assessable" = "#2E2E2E"
  )
  text_col <- "#2E2E2E"
  grid_col <- "#D9D9D9"
  # ------------------------------------------------------------
  # 4. Order factors
  # ------------------------------------------------------------
  mat_dat <- mat_raw %>%
    mutate(
      n_studies = as.numeric(n_studies),
      level_order = as.numeric(level_order),
```

```
      maturity_level = fct_reorder(
        maturity_level,
        level_order
      ),
      rob_overall = factor(
        rob_overall,
        levels = c(
          "Low",
          "Unclear",
          "High",
          "Not assessable"
        )
      )
    )
  .review_validate_maturity(mat_dat)

  totals <- mat_dat %>%
    group_by(maturity_level) %>%
    summarise(
      n_total = sum(n_studies),
      .groups = "drop"
    )
  message(
    "Total number of studies: ",
    sum(totals$n_total)
  )
  # ------------------------------------------------------------
  # 5. Create figure
  # ------------------------------------------------------------
  p_maturity_rob <- ggplot(
    mat_dat,
    aes(
      x = maturity_level,
      y = n_studies,
      fill = rob_overall
    )
  ) +
    geom_col(
      position = position_stack(reverse = TRUE),
      width = 0.62,
      colour = "white",
      linewidth = 0.45
    ) +
    geom_text(
      aes(
        label = ifelse(
          n_studies >= 2,
          n_studies,
          ""
        ),
        colour = rob_overall
      ),
      position = position_stack(
        vjust = 0.5,
        reverse = TRUE
      ),
      size = 3.6,
      fontface = "bold",
      show.legend = FALSE
    ) +
    scale_fill_manual(
      values = rob_cols_nature,
      drop = FALSE,
      guide = guide_legend(nrow = 1)
    ) +
    scale_colour_manual(
      values = rob_label_cols,
      guide = "none"
    ) +
    scale_x_discrete(
      labels = function(x) str_wrap(x, width = 14)
    ) +
    scale_y_continuous(
      limits = c(
        0,
        max(totals$n_total) * 1.05
      ),
      expand = expansion(
        mult = c(0, 0)
      )
    ) +
    labs(
```

```
      x = "Translational maturity level (increasing from left to right)",
      y = "Number of studies",
      fill = NULL
    ) +
    theme_minimal(
      base_size = 14
    ) +
    theme(
      text = element_text(
        colour = text_col
      ),
      axis.text = element_text(
        colour = text_col
      ),
      axis.text.x = element_text(
        size = 11
      ),
      axis.title.x = element_text(
        size = 13,
        margin = margin(
          t = 10
        )
      ),
      axis.title.y = element_text(
        size = 14,
        margin = margin(
          r = 8
        )
      ),
      panel.grid.major.x = element_blank(),
      panel.grid.minor = element_blank(),
      panel.grid.major.y = element_line(
        colour = grid_col,
        linewidth = 0.4
      ),
      legend.position = "bottom",
      legend.direction = "horizontal",
      legend.text = element_text(
        size = 11
      ),
      legend.key.size = grid::unit(0.9, "lines"),
      legend.margin = margin(
        t = 6
      ),
      plot.margin = margin(
        t = 10,
        r = 20,
        b = 10,
        l = 10
      )
    )
  # Display figure
  print(p_maturity_rob)

  invisible(list(
    p_maturity_rob = p_maturity_rob,
    mat_dat = mat_dat,
    totals = totals
  ))

}

# ============================================================================
# SECTION: maturity_readiness_combined
# ============================================================================
analysis_maturity_readiness_combined <- function(data_dir = "data", output_dir = "output") {

  if (!dir.exists(output_dir)) {
    dir.create(output_dir, recursive = TRUE)
  }

  # ============================================================
  # Combined figure
  #
  # a  Risk of Bias by Translational Maturity
  # b  Implementation-readiness indicators
  #
  # ============================================================


  # ============================================================
  # PANEL A
```

```
# Risk of Bias by Translational Maturity
# ==============================================================

# --------------------------------------------------------------
# 1. Load input
# --------------------------------------------------------------

Maturity_RoB_Input <- read_excel(.review_input(data_dir, "Maturity_RoB_Input.xlsx"))


# --------------------------------------------------------------
# 2. Prepare data
# --------------------------------------------------------------

mat_raw <- Maturity_RoB_Input %>%
  rename_with(
    ~ str_replace_all(
      str_trim(.x),
      "\\s+",
      "_"
    )
  )


# --------------------------------------------------------------
# 3. Check required columns
# --------------------------------------------------------------

required_cols_a <- c(
  "level_order",
  "maturity_level",
  "rob_overall",
  "n_studies"
)

missing_cols_a <- setdiff(
  required_cols_a,
  names(mat_raw)
)

if (length(missing_cols_a) > 0) {
  stop(
    paste0(
      "The following columns are missing in Maturity_RoB_Input: ",
      paste(missing_cols_a, collapse = ", ")
    )
  )
}


# --------------------------------------------------------------
# 4. RoB colour palette
# --------------------------------------------------------------

rob_cols_nature <- c(
  "Low"            = "#4C956C",
  "Unclear"        = "#E9C46A",
  "High"           = "#D95F5F",
  "Not assessable" = "#ABABAB"
)

rob_label_cols <- c(
  "Low"            = "white",
  "Unclear"        = "#2E2E2E",
  "High"           = "white",
  "Not assessable" = "#2E2E2E"
)

text_col <- "#2E2E2E"
grid_col <- "#D9D9D9"


# --------------------------------------------------------------
# 5. Order factors
# --------------------------------------------------------------

mat_dat <- mat_raw %>%
  mutate(
    n_studies = as.numeric(n_studies),
    level_order = as.numeric(level_order),

    maturity_level = fct_reorder(
```

```
      maturity_level,
      level_order
    ),

    rob_overall = factor(
      rob_overall,
      levels = c(
        "Low",
        "Unclear",
        "High",
        "Not assessable"
      )
    )
  )


# Totals per maturity level

.review_validate_maturity(mat_dat)

totals <- mat_dat %>%
  group_by(maturity_level) %>%
  summarise(
    n_total = sum(n_studies),
    .groups = "drop"
  )


# ------------------------------------------------------------
# 6. Create panel a
# ------------------------------------------------------------

p_a <- ggplot(
  mat_dat,
  aes(
    x = maturity_level,
    y = n_studies,
    fill = rob_overall
  )
) +
  geom_col(
    position = position_stack(reverse = TRUE),
    width = 0.62,
    colour = "white",
    linewidth = 0.45
  ) +
  geom_text(
    aes(
      label = ifelse(
        n_studies >= 2,
        n_studies,
        ""
      ),
      colour = rob_overall
    ),
    position = position_stack(
      vjust = 0.5,
      reverse = TRUE
    ),
    size = 3.5,
    fontface = "bold",
    show.legend = FALSE
  ) +
  scale_fill_manual(
    values = rob_cols_nature,
    drop = FALSE,
    guide = guide_legend(
      nrow = 1,
      byrow = TRUE
    )
  ) +
  scale_colour_manual(
    values = rob_label_cols,
    guide = "none"
  ) +

  # Wrap long maturity-level labels
  scale_x_discrete(
    labels = function(x) {
      str_wrap(
        x,
        width = 14
```

```
      )
    }
  ) +

  scale_y_continuous(
    limits = c(
      0,
      max(totals$n_total) * 1.06
    ),
    expand = expansion(
      mult = c(0, 0)
    )
  ) +

  labs(
    x = "Translational maturity level (increasing from left to right)",
    y = "Number of studies",
    fill = NULL
  ) +

  theme_minimal(
    base_size = 12
  ) +

  theme(
    text = element_text(
      colour = text_col
    ),

    axis.text = element_text(
      colour = text_col
    ),

    # Optimised x-axis labels
    axis.text.x = element_text(
      size = 10,
      lineheight = 0.90,
      colour = text_col,
      margin = margin(t = 6)
    ),

    axis.text.y = element_text(
      size = 10
    ),

    axis.title.x = element_text(
      size = 11,
      margin = margin(t = 10)
    ),

    axis.title.y = element_text(
      size = 11,
      margin = margin(r = 8)
    ),

    panel.grid.major.x = element_blank(),

    panel.grid.minor = element_blank(),

    panel.grid.major.y = element_line(
      colour = grid_col,
      linewidth = 0.35
    ),

    legend.position = "bottom",
    legend.direction = "horizontal",
    legend.justification = "center",

    legend.title = element_blank(),

    legend.text = element_text(
      size = 10
    ),

    legend.key.size = grid::unit(
      0.85,
      "lines"
    ),

    legend.margin = margin(
      t = 5
    ),
```

```
    plot.margin = margin(
      t = 12,
      r = 12,
      b = 5,
      l = 8
    )
  )


# ============================================================
# PANEL B
# Implementation-readiness indicators
# ============================================================

# ------------------------------------------------------------
# 7. Load input
# ------------------------------------------------------------

implementation_path <- .review_input(data_dir, "Implementation_Readiness_Input.xlsx")

Implementation_Readiness_Input <- read_excel(
  implementation_path
)


# ------------------------------------------------------------
# 8. Read total number of included studies
# ------------------------------------------------------------

readme_data <- read_excel(
  implementation_path,
  sheet = "README"
)

n_total <- readme_data %>%
  filter(field == "n_total") %>%
  pull(value) %>%
  as.numeric()


# The denominator must be explicitly documented in the input workbook.
if (length(n_total) != 1L || is.na(n_total)) {
  stop("README must contain exactly one numeric n_total value.", call. = FALSE)
}

.review_assert_count(
  n_total,
  REVIEW_N_STUDIES,
  "Implementation-readiness denominator"
)


# ------------------------------------------------------------
# 9. Colour palette for panel b
# ------------------------------------------------------------

pyramid_palette_1 <- c(
  "Deep Navy"  = "#1F3B5B",
  "Petrol"     = "#264653",
  "Teal"       = "#2A9D8F",
  "Sage Green" = "#8AB17D",
  "Sand"       = "#E9C46A",
  "Terracotta" = "#E76F51"
)


# ------------------------------------------------------------
# 10. Prepare implementation-readiness data
# ------------------------------------------------------------

indicators <- Implementation_Readiness_Input %>%
  mutate(
    indicator_order = as.numeric(indicator_order),
    n_studies = as.numeric(n_studies),
    percent = as.numeric(percent)
  ) %>%
  arrange(indicator_order) %>%
  mutate(

    indicator = factor(
      indicator,
```

```
        levels = rev(indicator)
      ),

      bar_color = rep(
        unname(pyramid_palette_1),
        length.out = n()
      ),

      label = paste0(
        n_studies,
        "/",
        n_total,
        " (",
        sprintf("%.1f", percent),
        "%)"
      ),

      # Keep labels relatively close to bars
      label_x = percent + 1.6
    )

  .review_validate_readiness(indicators, n_total)


  # Upper x-axis limit, as in the standalone readiness figure: 80 %, extended in
  # 20 % steps if an indicator exceeds it.
  x_axis_max <- max(
    80,
    ceiling(max(indicators$percent, na.rm = TRUE) / 20) * 20
  )


  # ------------------------------------------------------------
  # 11. Create panel b
  # ------------------------------------------------------------

  p_b <- ggplot(
    indicators,
    aes(
      x = percent,
      y = indicator,
      fill = bar_color
    )
  ) +
    geom_col(
      width = 0.68,
      alpha = 0.95
    ) +

    geom_text(
      aes(
        x = label_x,
        label = label
      ),
      hjust = 0,
      size = 3.15,
      colour = "grey20"
    ) +

    scale_fill_identity() +

    scale_x_continuous(
      breaks = seq(
        0,
        x_axis_max,
        by = 20
      ),
      labels = function(x) {
        paste0(x, "%")
      },
      expand = expansion(
        mult = c(0, 0)
      )
    ) +

    coord_cartesian(
      xlim = c(0, x_axis_max),
      clip = "off"
    ) +

    labs(
      x = "Studies meeting criterion (%)",
```

```
      y = NULL
    ) +

    theme_minimal(
      base_size = 12
    ) +

    theme(
      legend.position = "none",

      text = element_text(
        colour = text_col
      ),

      panel.grid.major.y = element_blank(),

      panel.grid.minor = element_blank(),

      panel.grid.major.x = element_line(
        colour = grid_col,
        linewidth = 0.35
      ),

      axis.text.y = element_text(
        size = 9.2,
        colour = "grey15"
      ),

      axis.text.x = element_text(
        size = 9.2,
        colour = "grey25"
      ),

      axis.title.x = element_text(
        size = 10.5,
        colour = "grey20",
        margin = margin(t = 9)
      ),

      plot.background = element_rect(
        fill = "white",
        colour = NA
      ),

      panel.background = element_rect(
        fill = "white",
        colour = NA
      ),

      # Right margin needed for labels
      plot.margin = margin(
        t = 12,
        r = 45,
        b = 5,
        l = 6
      )
    )


  # ============================================================
  # COMBINE PANELS A AND B
  # ============================================================

  # ------------------------------------------------------------
  # 12. Combine panels
  # ------------------------------------------------------------
  # More horizontal space is deliberately assigned to panel a.

  p_maturity_readiness <- (
    p_a | p_b
  ) +
    plot_layout(
      widths = c(
        1.75,
        0.85
      )
    ) +
    plot_annotation(
      tag_levels = "a",
      tag_prefix = "",
      tag_suffix = "",
      theme = theme(
```

```
        plot.tag = element_text(
          size = 15,
          face = "bold",
          colour = text_col
        )
      )
    ) &
    theme(
      plot.tag = element_text(
        size = 15,
        face = "bold",
        colour = text_col
      )
    )


  # ------------------------------------------------------------
  # 13. Display combined figure
  # ------------------------------------------------------------

  print(p_maturity_readiness)

  invisible(list(
    p_maturity_readiness = p_maturity_readiness,
    mat_dat = mat_dat,
    indicators = indicators
  ))

}

# ============================================================================
# RUNNER: selected sections execute in separate function environments.
# ============================================================================
run_review_analysis <- function(sections = "bubble_map", data_dir = "data",
                                output_dir = "output", rob_file = NULL,
                                rob_sheet = NULL, heatmap_file = NULL,
                                heatmap_sheet = NULL, save_session = TRUE) {

  available <- c(
    "bubble_map", "model_landscape", "disease_landscape",
    "implementation_readiness", "rob_domains", "rob_overall", "rob_combined",
    "rob_heatmap", "maturity_rob", "maturity_readiness_combined"
  )

  # Sections that read the risk-of-bias workbook.
  rob_sections <- c("rob_domains", "rob_overall", "rob_combined", "rob_heatmap")

  if (length(sections) == 1L && identical(sections, "all")) sections <- available

  if (!length(sections) || anyNA(sections) || any(!sections %in% available)) {
    stop("Select sections from: ", paste(available, collapse = ", "),
         call. = FALSE)
  }

  if (!dir.exists(output_dir)) {
    dir.create(output_dir, recursive = TRUE)
  }

  if (save_session) {
    on.exit(
      writeLines(
        capture.output(sessionInfo()),
        file.path(output_dir, "sessionInfo_current_run.txt")
      ),
      add = TRUE
    )
  }

  results <- list()

  for (section in unique(sections)) {

    message("Running section: ", section)

    fn <- get(paste0("analysis_", section),
              envir = environment(run_review_analysis))

    args <- list(data_dir = data_dir, output_dir = output_dir)

    if (section %in% rob_sections) {
      args$rob_file <- rob_file
      args$rob_sheet <- rob_sheet
```

```
    }

    if (identical(section, "rob_heatmap")) {
      args$heatmap_file <- heatmap_file
      args$heatmap_sheet <- heatmap_sheet
    }

    results[[section]] <- do.call(fn, args)
  }

  invisible(results)
}
```
