# Supplementary material for: Large language models in rheumatology: applications, methodological quality and clinical translation – a systematic review
Source: RMD Open. 2026 Oct 28;12(4):e007538. doi: 10.1136/rmdopen-2026-007538 (PMC13723917; doi:10.1136/rmdopen-2026-007538)
Supplement: Supplementary file 2 [file rmdopen-12-4-s001.docx]

**Large Language Models and Rheumatology: Insights from a systematic review - Supplementary Material**

**Contents**

| **Supplementary Material 1.** LLM-assisted data extraction prompt | 2 |
| --- | --- |
| **Supplementary Material 1a.** Codes worksheet | 5 |
| **Supplementary Material 2.** Full search strategies | 9 |
| **Supplementary Figure 1.** Risk of bias per study and AQAT:RoB item | 14 |
| **Supplementary Table 1.** Characteristics, key reported results, and risk of bias of the 93 included studies | 16 |
| **Supplementary Table 2.** Study-level values and reasons for exclusion in the quantitative outcome synthesis | 25 |
| **Supplementary Table 3**. Studies that appeared to meet the eligibility criteria but were excluded after full-text assessment | 28 |

**Material 1: LLM-assisted data extraction prompt**
The prompt shown represents the standardized extraction framework used during the review. Structured data extraction from the 93 included full-text articles was supported using the ChatGPT web interface (OpenAI, San Francisco, CA, USA; chatgpt.com). The model displayed in the interface was GPT-5.4 Thinking, with the reasoning setting manually set to High. The extraction was conducted on **22 March 2026 (S001-S051) and between 13 July and 15 July 2026 (S052-S093).**

The web interface rather than the OpenAI application programming interface was used. Consequently, no API model identifier was available for the individual requests. Sampling parameters such as temperature, top-p, random seed, maximum output tokens and frequency or presence penalties were not exposed or controllable through the interface and were therefore not recorded.

“***Task***

*Extract structured data from the provided full-text publication for a systematic review of large language models and generative artificial intelligence in rheumatology.*

*Use only information explicitly reported in the supplied full text, tables, figures. Do not use external sources and do not infer missing information. Enter “Not reported”, “Not applicable” or “Unclear” when information cannot be determined reliably.*

*Complete and extract data into the provided excel sheet.*

***A. Study_Characteristics worksheet***

1. *Study_ID*
2. *Title*
3. *First_Author*
4. *Publication_Year*
5. *Journal*
6. *DOI*
7. *Country/Region*
8. *Condition*
9. *Study Design according to the authors*
10. *Study_Design — classify the actual evaluation design using the categories in the Codes worksheet*
11. *GenAI_Role*
12. *Application_domain*
13. *Data_Source_Type*
14. *Input_Modality*
15. *Reproducibility_Test*
16. *Prompt_Transparency*
17. *Rater_Groups*
18. *N_Participants*
19. *N_Questions_or_Items*
20. *N_Records_or_Documents*
21. *N_Articles*
22. *N_Raters*
23. *Comparator*
24. *Instruments/Metrics*

*Use the categories defined in the Codes worksheet for Study_Design, GenAI_Role, Application_domain, Data_Source_Type, Input_Modality, Reproducibility_Test, Prompt_Transparency, Rater_Groups and Condition.*

***B. Outcomes worksheet***

*Extract:*

1. *Study ID*
2. *Outcome ID*
3. *Outcome Domain — use the categories in the Codes worksheet*
4. *Grading/ Metric*
5. *Scale/Unit*
6. *Group/Subgroup*
7. *N_Items*
8. *LLM Model Name*
9. *Comparator*
10. *Value Comparator (N) — record the numerical comparator result and associated sample size, dispersion or range as reported*
11. *Effect/Interpretation — record the numerical LLM result where available; otherwise record the reported direction or qualitative interpretation*
12. *p-value/CI*
13. *Notes*

*Preserve all numerical values exactly as reported. Do not convert scales, pool results or calculate new effect estimates. Include means, standard deviations, medians, ranges, proportions and confidence intervals in their original format.*

*If several models, subgroups or time points are reported, create separate outcome rows rather than combining the results.*

*Finally Perform an independent extraction of this study.*“

Material 1a: Codes worksheet

Field names and codes are reproduced as recorded in that worksheet.

Study_Design

**Codes**

Cross-sectional study

Cross-sectional survey

Comparative evaluation

Validation study

Retrospective study

Prospective study

Methods/Protocol

Other/Unclear

GenAI_Role

**Codes**

Primary (LLM outputs evaluated)

Secondary (LLM used as tool)

Mixed

Not LLM-focused

Application

**Codes**

Patient education

Clinical decision support

Medical education

Text analysis / information extraction

Research support / evidence synthesis

Conversational agents / patient-facing interaction

Material 1a: Codes worksheet (continued)

Data_Source_Type

**Codes**

Patient questions / online queries

Guideline-derived questions/treatments

Google Trends (question sourcing)

Scientific articles (open access)

EHR / medical records

Exam questions (MCQ / images)

Clinical vignettes / case scenarios

Reference patient fact sheets

Survey questionnaire

Other/Not specified

Input_Modality

**Codes**

Text-only

Includes images

Documents (EHR/MR)

Mixed/Other

Transparency

**Codes**

Yes

Partial

No/Unclear

Reproducibility_Test

**Codes**

None/Not stated

Repeated (same protocol)

Repeated (different day)

Material 1a: Codes worksheet (continued)

Rater_Groups

**Codes**

Clinicians/experts

Patients/public respondents

Patients + clinicians

Automated

Not stated

LLM-as-judge

Condition

**Codes**

Rheumatoid arthritis (RA)

Psoriatic arthritis (PsA)

Ankylosing spondylitis / axSpA

Systemic lupus erythematosus (SLE)

Juvenile idiopathic arthritis (JIA)

Systemic sclerosis

Dermatomyositis / myositis

Gout / crystal arthropathy

Familial Mediterranean fever (FMF)

Osteoarthritis (OA)

disease not specified

Mixed diseases

Real world data

**Codes**

yes

no

Material 1a: Codes worksheet (continued)

Outcome_Domain

**Codes**

Accuracy

Quality / completeness / usefulness

Readability / comprehensibility

Reliability / agreement

Guideline concordance

Consistency/Reproducibility

Safety/Misinformation

Usability / acceptability / adoption

Attitudes/Trust

Diagnostic/classification performance

Disease activity

Patient-reported outcomes

Efficiency / productivity

Empathy

Response length / verbosity

Sentiment / emotion

Citation / reference quality

Risk appetite

Cost

**Material 2: Full search strategies**

**Search terms Pubmed:**

(

"Generative Artificial Intelligence"[Mesh]

OR "large language model*"[tiab]

OR LLM[tiab] OR LLMs[tiab] OR "LLM-based"[tiab] OR "LLM based"[tiab]

OR ChatGPT[tiab] OR "chat gpt"[tiab] OR "Chat-GPT"[tiab]

OR GPT-4[tiab] OR GPT4[tiab] OR GPT-3.5[tiab] OR GPT3.5[tiab]

OR Claude[tiab] OR "Anthropic Claude"[tiab]

OR Gemini[tiab] OR "Google Gemini"[tiab]

OR Llama[tiab] OR "LLaMA"[tiab] OR "Meta Llama"[tiab] OR lama[tiab]

OR "generative pretrained transformer"[tiab]

OR "generative ai"[tiab]

)

AND

(

"Rheumatology"[Mesh]

OR "Arthritis"[Mesh]

OR "Arthritis, Rheumatoid"[Mesh]

OR "Arthritis, Psoriatic"[Mesh]

OR "Axial Spondyloarthritis"[Mesh]

OR "Non-Radiographic Axial Spondyloarthritis"[Mesh]

OR "Connective Tissue Diseases"[Mesh]

OR "Vasculitis"[Mesh]

OR "Hereditary Autoinflammatory Diseases"[Mesh]

OR "Lupus Erythematosus, Systemic"[Mesh]

OR "Sjogren's Syndrome"[Mesh]

OR "Myositis"[Mesh]

OR "Scleroderma, Systemic"[Mesh]

OR rheumatol*[tiab]

OR arthritis[tiab]

OR "rheumatoid arthritis"[tiab] OR RA[tiab]

OR "psoriatic arthritis"[tiab] OR "psoriasis arthritis"[tiab] OR PsA[tiab]

OR "axial spondyloarthritis"[tiab] OR "axial spondylarthritis"[tiab] OR axSpA[tiab]

OR lupus[tiab]

OR "systemic lupus"[tiab]

OR "systemic lupus erythematosus"[tiab]

OR SLE[tiab]

OR ("LE"[tiab] AND lupus[tiab])

OR myositis[tiab] OR polymyositis[tiab] OR dermatomyositis[tiab] OR IIM[tiab]

OR sjogren*[tiab] OR "sicca syndrome"[tiab]

OR scleroderma[tiab]

OR "systemic sclerosis"[tiab]

OR SSc[tiab]

OR "connective tissue disease*"[tiab]

OR vasculitis*[tiab] OR angiitis[tiab]

OR autoinflammator*[tiab] OR autoinflammation[tiab]

OR "periodic fever"[tiab] OR "periodic fever syndrome*"[tiab]

)

NOT (animals[mh] NOT humans[mh])

**Search Terms Web of Science:**

(

(

"generative artificial intelligence" OR "generative ai" OR

("large language model" OR "large language models") OR

LLM OR LLMs OR

ChatGPT OR "chat gpt" OR "chat-gpt" OR

"GPT-4" OR GPT4 OR "GPT 4" OR

"GPT-3.5" OR GPT3.5 OR "GPT 3.5" OR

Claude OR "Anthropic Claude" OR

Gemini OR "Google Gemini" OR

Llama OR "LLaMA" OR "Meta Llama" OR

("generative pretrained transformer" OR "generative pretrained transformers")

)

AND

(

rheumatol* OR arthritis OR

"rheumatoid arthritis" OR (RA AND arthritis) OR

"psoriatic arthritis" OR "psoriasis arthritis" OR (PsA AND arthritis) OR

"axial spondyloarthritis" OR "axial spondylarthritis" OR axSpA OR

"non-radiographic axial spondyloarthritis" OR "non radiographic axial spondyloarthritis" OR

lupus OR "systemic lupus" OR "systemic lupus erythematosus" OR SLE OR

myositis OR polymyositis OR dermatomyositis OR IIM OR

sjogren* OR "sicca syndrome" OR

scleroderma OR "systemic sclerosis" OR SSc OR

"connective tissue disease" OR "connective tissue diseases" OR

vasculitis* OR angiitis OR

autoinflammator* OR autoinflammation OR

"periodic fever" OR ("periodic fever syndrome" OR "periodic fever"

)

)

**Search strategy Embase via Ovid:**

(

exp generative artificial intelligence/

OR exp large language model/

OR "large language model*".ti,ab.

OR LLM.ti,ab. OR LLMs.ti,ab.

OR "LLM based".ti,ab. OR "LLM-based".ti,ab.

OR ChatGPT.ti,ab. OR "chat gpt".ti,ab. OR "Chat-GPT".ti,ab.

OR "GPT-4".ti,ab. OR GPT4.ti,ab.

OR "GPT-3.5".ti,ab. OR "GPT3.5".ti,ab.

OR Claude.ti,ab. OR (Anthropic adj1 Claude).ti,ab.

OR Gemini.ti,ab. OR "Google Gemini".ti,ab.

OR Llama.ti,ab. OR "LLaMA".ti,ab. OR "Meta Llama".ti,ab. OR lama.ti,ab.

OR "generative pretrained transformer".ti,ab.

OR "generative ai".ti,ab.

)

AND

(

exp rheumatology/

OR exp arthritis/

OR exp rheumatoid arthritis/

OR exp psoriatic arthritis/

OR exp axial spondyloarthritis/

OR exp nonradiographic axial spondyloarthritis/

OR exp connective tissue disease/

OR exp vasculitis/

OR exp autoinflammatory disease/

OR exp systemic lupus erythematosus/

OR exp sjogren syndrome/

OR exp myositis/

OR exp systemic sclerosis/

OR rheumatol*.ti,ab.

OR arthritis.ti,ab.

OR "rheumatoid arthritis".ti,ab. OR RA.ti,ab.

OR "psoriatic arthritis".ti,ab. OR "psoriasis arthritis".ti,ab. OR PsA.ti,ab.

OR "axial spondyloarthritis".ti,ab. OR "axial spondylarthritis".ti,ab. OR axSpA.ti,ab.

OR lupus.ti,ab.

OR "systemic lupus".ti,ab.

OR "systemic lupus erythematosus".ti,ab.

OR SLE.ti,ab.

OR (LE.ti,ab. AND lupus.ti,ab.)

OR myositis.ti,ab. OR polymyositis.ti,ab. OR dermatomyositis.ti,ab. OR IIM.ti,ab.

OR sjogren*.ti,ab. OR "sicca syndrome".ti,ab.

OR scleroderma.ti,ab.

OR "systemic sclerosis".ti,ab.

OR SSc.ti,ab.

OR "connective tissue disease*".ti,ab.

OR vasculitis*.ti,ab. OR angiitis.ti,ab.

OR autoinflammator*.ti,ab. OR autoinflammation.ti,ab.

OR "periodic fever".ti,ab. OR "periodic fever syndrome*".ti,ab.

)

NOT (exp animal/ NOT exp human/)

**Supplementary Figure 1:** Risk of bias per study and AQAT:RoB item. Studies for which AQAT:RoB was not applicable are not shown.

**Supplementary Table 1. Characteristics, key reported results, and risk of bias of the 93 included studies**

| **ID first author, year** | **First author, year, country, DOI** | **Condition** | **Application domain** | **Design** | **LLM(s) evaluated** | **Data source and sample size** | **Comparator** | **Outcome domains assessed** | **Key reported results** | **Prompt reporting, reproducibility, RoB** |
| --- | --- | --- | --- | --- | --- | --- | --- | --- | --- | --- |
| S001 Moss AS 2026 | Moss AS 2026 USA doi: 10.1007/s10067-025-07746-9 | Disease not specified | Patient education | Comparative evaluation | ChatGPT 3.5 | Reference patient fact sheets \| 96 items; 96 records | Original ACR patient education materials | Readability | Gunning Fog Index: ChatGPT-3.5 10.70 ± 1.39 vs ACR materials 14.24 ± 1.85 — Flesch-Kincaid Grade Level: ChatGPT-3.5 8.29 ± 1.21 vs ACR materials 11.50 ± 1.74. | Prompts: Yes Repro: Repeated (same protocol) RoB: Not applicable |
| S002 Carrasco Laraña A 2026 | Carrasco Laraña A 2026 Spain doi: 10.1016/j.rceng.2025.502418 | Disease not specified | Clinical decision support | Validation study | GPT-4 | EHR / medical records \| 101 participants; 101 records | Final specialist consensus diagnosis | Accuracy | Overall diagnostic accuracy: GPT-4 Phase 1: 82.18%; phase 2: 97.03% (98/101) — Disease-level accuracy – SLE: GPT-4 Phase 1: 92%; phase 2: 100%. | Prompts: No/Unclear Repro: None/not stated RoB: Not applicable |
| S003 Yang JW 2024 | Yang JW 2024 USA doi: 10.1016/j.arth.2024.01.029 | Osteoarthritis | Clinical decision support | Comparative evaluation | ChatGPT; Google Bard | Guideline-derived questions/treatments \| 20 items; 2 raters | Google Bard; AAOS clinical practice guideline | Guideline concordance; Safety/misinformation | Treatment concordance with AAOS CPG: ChatGPT 16/20 (80%) — Treatment concordance with AAOS CPG: Google Bard 12/20 (60%). | Prompts: Yes Repro: None/not stated RoB: Unclear |
| S004 Pillai J 2023 | Pillai J 2023 USA doi: 10.1016/j.jtauto.2023.100213 | Mixed diseases | Clinical decision support | Comparative evaluation | GPT-4; GPT-3.5; LLaMA 2 | Clinical vignettes / case scenarios \| 40 items | Internal medicine physician; GPT-3.5; LLaMA 2 | Accuracy | Diagnostic accuracy (correct ID), Top-1: GPT-4 0% vs internist 35% (p=0.0083) — Diagnostic accuracy (correct ID), Top-1: GPT-3.5 0% vs internist 35% (p=0.0083). | Prompts: Yes Repro: None/not stated RoB: Unclear |
| S005 Labinsky H 2024 | Labinsky H 2024 Germany doi: 10.1007/s00296-024-05675-5 | Disease not specified | Clinical decision support | Comparative evaluation | GPT-4; GPT-3.5 | Guideline-derived questions/treatments \| 20 items; 4 raters | Rheumatology board treatment plans; GPT-3.5 vs GPT-4 | Attitudes/trust; Quality/completeness/usefulness; Safety/misinformation | Overall quality rating: GPT-4 3.25 (1.75–4.5) vs Rheumatology board 4.0 (3.75–4.75) (p<0.0001) — Overall quality rating: GPT-3.5 3.25 (1.5–4.25) vs Rheumatology board 4.0 (3.75–4.75) (p<0.0001). | Prompts: Partial Repro: None/not stated RoB: Unclear |
| S006 Tong L 2024 | Tong L 2024 China doi: 10.1186/s13018-024-04996-2 | GIOP | Patient education | Comparative evaluation | ChatGPT-3.5; ChatGPT-4; Google Gemini | Patient questions / online queries \| 59 items; 3 raters | ChatGPT-3.5; ChatGPT-4; Google Gemini; 2022 ACR-GIOP guideline | Consistency/reproducibility; Guideline concordance; Readability | Good answer rate: ChatGPT-4 64% — Good answer rate: Google Gemini 32%. | Prompts: Yes Repro: None/not stated RoB: Unclear |
| S007 Uzun Bektaş A 2025 | Uzun Bektaş A 2025 Türkiye doi: 10.1007/s00431-025-06318-y | FMF | Clinical decision support | Comparative evaluation | ChatGPT-4o; LLaMA-3.1 | Guideline-derived questions/treatments \| 31 items; 2 raters | LLaMA-3.1; SHARE/EULAR recommendations | Guideline concordance; Safety/misinformation | Fully aligned, complete, and accurate responses: ChatGPT-4o 25/31 (80.6%) vs LLaMA 3.1 16/31 (51.6%; reported as significant) — Entirely contradictory responses: 0/31 (0%) vs 4/31 (9.7%), respectively. | Prompts: Yes Repro: None/not stated RoB: Unclear |
| S008 Haase I 2024 | Haase I 2024 Germany doi: 10.1016/S2665-9913(24)00056-0 | SLE | Patient education | Comparative evaluation | ChatGPT-4 | Patient questions / online queries \| 100 items; 3 raters | Lupus100.org physician-generated answers | Attitudes/trust; Empathy; Quality/completeness/usefulness; Readability | Word count: ChatGPT-4 372 (52) vs Lupus100.org 241 (135) (p=0.001) — Quality score: ChatGPT-4 4.55 (0.65) [4.48–4.62] vs Lupus100.org 4.31 (0.72) [4.23–4.39] (p=0.001). | Prompts: Yes Repro: None/not stated RoB: Unclear |
| S009 Çelik NÇ 2025 | Çelik NÇ 2025 Türkiye doi: 10.1007/s10067-025-07417-9 | Rheumatoid arthritis | Clinical decision support | Comparative evaluation | ChatGPT-4 | Guideline-derived questions/treatments \| 50 items | EULAR recommendations; baseline vs day 14 | Accuracy; Consistency/reproducibility; Guideline concordance | Correct MCQ responses: 43/50 (86%) at baseline vs 45/50 (90%) at day 14 (p=0.001) — Guideline-congruent responses: 48/50 (96%) at baseline vs 49/50 (98%) at day 14. | Prompts: No/Unclear Repro: Repeated (different day) RoB: Unclear |
| S010 Sari F 2026 | Sari F 2026 Türkiye doi: 10.1016/j.jadohealth.2025.09.015 | Myositis | Patient education | Comparative evaluation | ChatGPT-4 | Patient questions / online queries \| 70 items; 2 raters | No direct comparator | Accuracy; Quality/completeness/usefulness; Readability; Reliability/agreement | Accuracy rating: ChatGPT-4 4.26 (3.90–5.00) — Flesch Reading Ease: ChatGPT-4 47.79 (42.92–55.61). | Prompts: Partial Repro: None/not stated RoB: Unclear |
| S011 Chen 2025 | Chen 2025 China; USA doi: 10.3389/fdgth.2025.1530442 | Disease not specified | Clinical decision support | Comparative evaluation | ChatGPT-3.5; ChatGPT-4.0; Gemini | Clinical vignettes / case scenarios \| 46 items; 3 participants; 3 raters | Between-model comparison | Accuracy; Quality/completeness/usefulness; Reliability/agreement; Safety/misinformation | Correctness: ChatGPT-4.0 4.28 ± 0.44 vs ChatGPT-3.5 3.74 ± 0.76 and Gemini 3.75 ± 0.80 (p=0.0001 and p=0.0002) — Overall five-dimension score: 199.8 ± 10.4 vs 175.7 ± 16.6 and 179.1 ± 11.8 (p=0.009 and p=0.001). | Prompts: Partial Repro: None/not stated RoB: Unclear |
| S012 Çelik 2025 | Çelik 2025 Türkiye doi: 10.1007/s10067-025-07771-8 | Axial spondyloarthritis | Patient education | Comparative evaluation | ChatGPT-3.5; ChatGPT-4o; DeepSeek-R1; DeepSeek-V3 | Patient questions / online queries \| 10 items; 2 raters | Human experts/clinicians; between-model comparison | Readability; Reliability/agreement | Flesch Reading Ease: ChatGPT-4o 11.28, DeepSeek-R1 13.32, ChatGPT-3.5 5.29, DeepSeek-V3 14.93 — Flesch-Kincaid Grade Level: 15.33, 15.50, 15.93, and 15.41, respectively. | Prompts: Yes Repro: None/not stated RoB: High |
| S013 Mendoza-Pinto 2025 | Mendoza-Pinto 2025 China; United States doi: 10.3389/fdgth.2025.1623399 | Disease not specified | Patient education | Comparative evaluation | ChatGPT | Scientific articles (open access) \| 12 items; 12 articles; 2 raters | Other LLMs | Readability; Response length | FKGL: ChatGPT 10.52 ± 2.17 vs Original rheumatology article text 15.06 ± 1.68 (p<0.0001) — SMOG: ChatGPT 9.48 ± 1.86 vs Original rheumatology article text 14.08 ± 1.21 (p<0.0001). | Prompts: Yes Repro: None/not stated RoB: High |
| S014 Alluri 2025 | Alluri 2025 India; Hungary; Sint Maarten doi: 10.7759/cureus.90600 | Disease not specified | Patient education | Comparative evaluation | ChatGPT-4.0; DeepSeek | Patient questions / online queries \| 3 records | Between-model comparison | Readability; Reliability/agreement | Word count: DeepSeek AI 507.67 ± 127.69 vs ChatGPT 548.67 ± 193.74 (p=0.775) — Sentence count: DeepSeek AI 77.67 ± 29.14 vs ChatGPT 83.00 ± 18.33 (p=0.802). | Prompts: Yes Repro: None/not stated RoB: High |
| S015 Coskun 2023 | Coskun 2023 Turkey; Germany doi: 10.1007/s00296-023-05473-5 | Disease not specified | Patient education | Comparative evaluation | ChatGPT-3.5; GPT-4; Google Bard; Microsoft Bing | Patient questions / online queries \| 23 items; 2 raters | Between-model comparison | Accuracy; Quality/completeness/usefulness | Correct-answer rate: GPT-4 100%, GPT-3.5 100%, Bard 73.91%, Bing 73.91% — Completely correct outputs: GPT-4 100%, GPT-3.5 86.96%, Bard and Bing 60.87% each. | Prompts: No/Unclear Repro: None/not stated RoB: High |
| S016 Forte 2025 | Forte 2025 Italy; United States doi: 10.1016/j.ard.2025.11.012 | Psoriatic arthritis | Patient education | Comparative evaluation | ChatGPT-4 | Patient questions / online queries \| 32 items; 76 participants; 12 physician and 67 patient raters | Human experts/clinicians | Accuracy; Attitudes/trust; Quality/completeness/usefulness; Readability | Accuracy score: ChatGPT-4 5 vs Rheumatologist-generated answers 5 (not significant) — Flesch Italian readability score: ChatGPT-4 90.07 ± 1.69 vs Rheumatologist-generated answers 88.61 ± 1.99 (p<0.001). | Prompts: Yes Repro: None/not stated RoB: Low |
| S017 Hayıroğlu 2025 | Hayıroğlu 2025 Turkey; USA doi: 10.1007/s10067-025-07745-w | FMF | Patient education | Comparative evaluation | ChatGPT-4.0; Gemini; Grok | Patient questions / online queries \| 49 items; 2 raters | Between-model comparison | Accuracy; Consistency/reproducibility; Safety/misinformation | Comprehensive/correct responses: Gemini 87.7%, Grok 83.6%, ChatGPT-4.0 81.6% — mDISCERN ranges: Gemini 4.10–4.63, Grok 3.64–4.56, ChatGPT-4.0 3.63–4.58 (not significant). | Prompts: Yes Repro: Repeated (different day) RoB: High |
| S018 Sari 2025 | Sari 2025 China; Turkey; United States doi: 10.1007/s10067-025-07789-y | Axial spondyloarthritis | Patient education | Comparative evaluation | ChatGPT-4; DeepSeek-V3 | Patient questions / online queries \| 50 items; 3 raters | Between-model comparison | Accuracy; Quality/completeness/usefulness; Readability; Reliability/agreement | mDISCERN: DeepSeek-V3 4 (3–4) vs ChatGPT-4 3 (3–3) (p<0.001) — Flesch Reading Ease: DeepSeek-V3 28.77 ± 17.77 vs ChatGPT-4 30.4 ± 14.37 (not significant). | Prompts: Yes Repro: None/not stated RoB: High |
| S019 Wu 2025 | Wu 2025 China; USA doi: 10.1016/j.knee.2025.06.007 | Osteoarthritis | Clinical decision support | Comparative evaluation | ChatGPT-4.0; DeepSeek-R1 | Guideline-derived questions/treatments \| 17 items; 2 raters | Between-model comparison | Guideline concordance; Reliability/agreement | Concordant recommendations: ChatGPT-4.0 59% vs DeepSeek-R1 71% (not significant) — Discordant recommendations: 12% vs 18% (not significant). | Prompts: Yes Repro: None/not stated RoB: High |
| S020 Xu 2024 | Xu 2024 China; USA; Hong Kong; Singapore doi: 10.1093/rheumatology/keae238 | SLE | Patient education | Comparative evaluation | ChatGPT-4 | Patient questions / online queries \| 95 items; 4 physician and 6 patient raters | Human experts/clinicians | Accuracy; Empathy; Quality/completeness/usefulness; Readability; Usability/acceptability | Selected item ratings (physicians/patients): ChatGPT-4 9.30 ± 1.11/9.39 ± 0.57 vs specialist answers 5.38 ± 2.47/8.76 ± 1.07 (p<0.001/p=0.002) — A second item was rated 6.83 ± 1.72/9.21 ± 0.67 vs 8.33 ± 1.83/9.33 ± 0.68 (p<0.001/p=0.459). | Prompts: Partial Repro: Repeated (same protocol) RoB: Low |
| S021 Nakaphan 2025 | Nakaphan 2025 USA; Germany doi: 10.1007/s00296-025-06053-5 | Disease not specified | Medical education | Comparative evaluation | GPT-3.5; GPT-4; Claude Sonnet 4; Gemini; Perplexity AI; DeepSeek; OpenEvidence | Exam questions (MCQ/images) \| 75 records | Between-model comparison | Accuracy; Safety/misinformation | Text-only MCQ accuracy: GPT-3.5 92%, GPT-4 92%, Claude Sonnet 4 94%, Gemini 92%, Perplexity AI 90%, DeepSeek 96%, OpenEvidence 92% — Image-based accuracy: 44%, 44%, 16%, 56%, 44%, not assessed, and not assessed, respectively. | Prompts: Partial Repro: None/not stated RoB: Unclear |
| S022 Is 2024 | Is 2024 Turkey; USA doi: 10.1007/s10067-024-07154-5 | Disease not specified | Medical education | Comparative evaluation | ChatGPT-4o; Gemini | Exam questions (MCQ/images) \| 420 items | Between-model comparison | Accuracy; Consistency/reproducibility | Correct answers first run: ChatGPT-4o 86.9% vs Google Gemini 60.2% (p<0.001) — Correct answers second run: ChatGPT-4o 86.7% vs Google Gemini 60.5% (p<0.001). | Prompts: Partial Repro: Repeated (same protocol) RoB: Not applicable |
| S023 Du 2025 | Du 2025 China doi: 10.2196/67830 | Osteoarthritis | Patient education | Comparative evaluation | GPT-4 | Clinical vignettes / case scenarios \| 50 items; 50 participants; 2 raters | Clinician-created guidance | Accuracy; Efficiency/productivity; Quality/completeness/usefulness; Readability; Safety/misinformation | Accuracy score: GPT-4 5.31 ± 1.73 vs clinician-created guidance 4.76 ± 1.10 (p=0.05) — Flesch-Kincaid Grade Level: 11.56 ± 1.08 vs 12.67 ± 0.95 (p<0.001). | Prompts: Yes Repro: None/not stated RoB: Unclear |
| S024 Yao 2025 | Yao 2025 China doi: 10.1016/j.ijmedinf.2025.106026 | Disease not specified | Clinical decision support | Comparative evaluation | GPT-4o-mini; GPT-4o; ERNIE-4.0-Turbo-128K; Meta-LLaMA-3.1-405B-Instruct | Clinical vignettes / case scenarios \| 1,122 items; one physician and GPT-4o-mini as judges | Between-model comparison | Accuracy; Other | Hit@1 zero-shot: GPT-4o-mini 81.8%, GPT-4o 82.4%, ERNIE-4.0 82.9%, LLaMA 3.1 82.7% — Few-shot: 82.2%, 85.9%, 81.1%, and 80.9%, respectively (p<0.05). | Prompts: Partial Repro: None/not stated RoB: High |
| S025 Lushington 2025 | Lushington 2025 USA doi: 10.3390/diagnostics15182409 | SLE | Clinical decision support | Retrospective study | Claude 3.5 | EHR / medical records \| 78 items; 78 participants | Clinical determination / expert classification | Classification performance | Sensitivity: Claude 3.5 0.78 ± 0.09 (95% CI) — Specificity: Claude 3.5 0.60 ± 0.11 (95% CI). | Prompts: No/Unclear Repro: Repeated (same protocol) RoB: Not applicable |
| S026 Krusche 2023 | Krusche 2023 Germany doi: 10.1007/s00296-023-05464-6 | Disease not specified | Clinical decision support | Comparative evaluation | ChatGPT-4 | Clinical vignettes / case scenarios \| 132 items from 33 physicians in the original publication; 21 vignettes in the supplied supplement | Ada; physicians | Accuracy; Classification performance | Correct top diagnosis: ChatGPT-4 0.35 vs Physicians 0.39 (p=0.3) — Correct diagnosis among top 3: ChatGPT-4 0.6 vs Physicians 0.55 (p=0.38). | Prompts: Partial Repro: None/not stated RoB: High |
| S027 Özbek 2025 | Özbek 2025 United Kingdom doi: 10.4274/tod.galenos.2024.76743 | Disease not specified | Patient education | Comparative evaluation | ChatGPT; Google Bard; Microsoft Copilot | Patient questions / online queries \| 30 items; 2 raters | ChatGPT, Bard, Copilot | Quality/completeness/usefulness; Readability | Flesch Reading Ease: ChatGPT 31.65, Copilot 42.35, Bard 43.00 (p<0.001) — Flesch-Kincaid Grade Level: 12.35, 10.50, and 10.45, respectively (p=0.001). | Prompts: Yes Repro: None/not stated RoB: High |
| S028 Ye 2024 | Ye 2024 Canada doi: 10.1002/art.42737 | Disease not specified | Patient education | Comparative evaluation | ChatGPT-4 | Patient questions / online queries \| 10 patient-facing responses; 30 physician-facing responses; 17 participants; 4 raters | Physician-generated responses | Accuracy; Attitudes/trust; Quality/completeness/usefulness; Readability | Accuracy: ChatGPT-4 6.48 ± 2.07 vs physician responses 9.08 ± 0.64 (p<0.0001) — Readability: ChatGPT-4 7.90 ± 0.90 vs physician responses 7.80 ± 0.75 (p=0.5905). | Prompts: Partial Repro: None/not stated RoB: High |
| S029 Li 2025 | Li 2025 China doi: 10.2196/76458 | SLE | Patient education | Comparative evaluation | ChatGPT-4o | Web source / LLM generated \| 20 items; 2 raters | Google Web Search; Other LLMs | Accuracy; Quality/completeness/usefulness; Readability | Accuracy: ChatGPT-4o 5.83 ± 0.49 vs Google Web Search 4.75 ± 0.94 — Flesch Reading Ease: ChatGPT-4o 17.3 vs Google Web Search 20.0. | Prompts: Yes Repro: None/not stated RoB: High |
| S030 Erden 2025 | Erden 2025 Türkiye doi: 10.5114/reum/207526 | Disease not specified | Patient education | Comparative evaluation | ChatGPT-4 | Physician-generated questions \| 60 items (4 per disease); 3 raters | ACR fact sheets | Quality/completeness/usefulness; Readability; Reliability/agreement | FKGL: ChatGPT-4 12.57 ± 1.0 vs ACR fact sheets 11.14 ± 1.42 (p=0.007) — Flesch Reading Ease: ChatGPT-4 35.83 ± 5.5 vs ACR fact sheets 43.75 ± 9.40 (p=0.005). | Prompts: Yes Repro: None/not stated RoB: Unclear |
| S031 Munguía-Realpozo 2025 | Munguía-Realpozo 2025 Mexico doi: 10.1177/09612033251324501 | SLE | Patient education | Comparative evaluation | ChatGPT-3.5; ChatGPT-4; Microsoft Copilot | Patient questions / online queries \| 13 items; 2 raters | Between-model comparison | Accuracy; Quality/completeness/usefulness | Mean accuracy score: ChatGPT-4 5.92 ± 0.28, GPT-3.5 5.92 ± 0.28, Copilot 5.85 ± 0.38 (not significant) — Completely accurate responses: ChatGPT-4 100%, GPT-3.5 100%, Copilot 84.6% (not significant). | Prompts: Partial Repro: Repeated (same protocol) RoB: Unclear |
| S032 Wang 2026 | Wang 2026 China; United States; Spain doi: 10.1016/j.ijmedinf.2025.106172 | Disease not specified | Patient education | Comparative evaluation | ChatGPT-4.0; Microsoft Copilot; Gemini 2.0; DeepSeek-R1 | Guideline-derived questions/treatments \| 250 items; 2 raters | Between-model comparison | Accuracy; Quality/completeness/usefulness; Readability | Six-point rating: DeepSeek-R1 5.35 ± 0.56, ChatGPT-4.0 5.23 ± 0.60, Gemini 2.0 5.12 ± 0.74, Copilot 4.77 ± 1.01 (p<0.001) — Flesch Reading Ease: 79.95 ± 8.91, 78.42 ± 7.26, 80.26 ± 6.37, and 83.22 ± 4.75, respectively (p<0.001). | Prompts: Yes Repro: None/not stated RoB: Unclear |
| S033 Ren 2025 | Ren 2025 China doi: 10.1136/bmjopen-2024-097528 | Axial spondyloarthritis | Patient education | Comparative evaluation | ChatGPT-3.5; ChatGPT-4.0; Gemini 1.5 Flash; Kimi | Patient questions / online queries \| 15 items; 178 participants; 4 raters | Medical guidelines; between-model comparison | Quality/completeness/usefulness; Usability/acceptability | Mean scientificity scores: Kimi 4.77, ChatGPT-4.0 4.68, ChatGPT-3.5 4.43, Gemini 1.5 Flash 4.13, guideline answers 4.72 — Of 178 patients, 54.8% preferred LLM responses, 16.4% preferred guideline information, and 38.7% considered guideline and at least one LLM response equally good. | Prompts: Partial Repro: None/not stated RoB: Unclear |
| S034 Megafu 2025 | Megafu 2025 USA doi: 10.1016/j.jseint.2025.03.011 | Osteoarthritis | Clinical decision support | Comparative evaluation | ChatGPT-4.0; Gemini | Guideline-derived questions/treatments \| 20 items; 2 participants | Between-model comparison | Guideline concordance | Guideline concordance for recommended treatments: ChatGPT-4.0 100%, Gemini 50%; for non-recommended treatments: 80% and 100%; for neutral recommendations: 100% and 40%. | Prompts: Yes Repro: None/not stated RoB: Unclear |
| S035 Kara 2025 | Kara 2025 Türkiye doi: 10.1371/journal.pone.0326351 | Axial spondyloarthritis | Patient education | Comparative evaluation | ChatGPT 4.0; Perplexity; Gemini | Patient questions / online queries (keywords only) \| 25 items | Between-model comparison | Readability; Reliability/agreement | Pairwise readability comparisons were not significant; readability ranked Gemini easiest, followed by ChatGPT-4.0 and Perplexity — Median DISCERN/GQS/EQIP scores were 2/3/48.50 for ChatGPT-4.0, 3/4/64.95 for Perplexity, and 2/3/48.65 for Gemini. | Prompts: Yes Repro: None/not stated RoB: Not applicable |
| S036 Kılınç 2025 | Kılınç 2025 Türkiye doi: 10.1007/s10067-025-07648-w | Axial spondyloarthritis | Patient education | Cross-sectional study | ChatGPT 4.0 | Patient questions / online queries \| 75 items; 2 raters | No direct comparator | Accuracy; Quality/completeness/usefulness; Readability | Six-point ratings were 5.32 ± 1.40 for patient questions, 5.36 ± 1.10 for recommendation-derived questions, and 4.24 ± 1.96 for physician-perspective questions — Corresponding three-point ratings were 2.76 ± 0.50, 2.72 ± 0.45, and 2.36 ± 0.63. | Prompts: Yes Repro: None/not stated RoB: Low |
| S037 Usen 2025 | Usen 2025 Türkiye doi: 10.3390/diagnostics15121455 | Axial spondyloarthritis | Clinical decision support | Comparative evaluation | ChatGPT-3.5; ChatGPT-4.0; Gemini 2.0 Flash | Guideline-derived questions/treatments \| 15 items; 2 raters | Between-model comparison | Quality/completeness/usefulness; Readability; Reliability/agreement | Flesch Reading Ease, median (range): ChatGPT-3.5 6.74 (−0.10–20.48), ChatGPT-4.0 4.14 (−2.94–16.73), Gemini 2.0 Flash 15.31 (1.13–27.01) — FKGL: 17.8 (14.6–19.0), 17.1 (15.0–20.3), and 15.7 (13.6–17.9), respectively. | Prompts: Yes Repro: None/not stated RoB: Low |
| S038 Sood 2025 | Sood 2025 USA doi: 10.1097/RHU.0000000000002198 | Disease not specified | Patient education | Cross-sectional study | ChatGPT-3.5 | Guideline-derived questions/treatments \| 20 items; 5 raters | Human experts/clinicians; ACR fact sheets | Reliability/agreement | Mean accuracy was 4.04/5 (80% of responses scored ≥4); mean completeness was 2.30/3 (95% scored ≥2) — Inter-rater agreement was low (Fleiss κ=0.14 for accuracy and κ=0.19 for completeness). | Prompts: Yes Repro: None/not stated RoB: Unclear |
| S039 Tai 2024 | Tai 2024 Hong Kong; Japan doi: 10.3233/SHTI240557 | SLE | Text analysis / information extraction | Comparative evaluation | LLaMA 2; Mistral; BioMistral | EHR / medical records \| 20 items | Between-model comparison | Accuracy | Mistral achieved the highest extraction performance (F1=0.996, 95% CI 0.993–0.999); Mistral and BioMistral outperformed LLaMA 2. | Prompts: Partial Repro: Repeated (same protocol) RoB: Not applicable |
| S040 Berghea 2025 | Berghea 2025 Romania doi: 10.3390/app15105700 | Rheumatoid arthritis | Clinical decision support | Comparative evaluation | ChatGPT 4.5; Gemini 2.0; Qwen 2.5 MAX; DeepSeek-V3; Perplexity | Clinical vignettes / case scenarios \| 20 items | Between-model comparison | Accuracy; Consistency/reproducibility; Other | All five systems independently ranked the same RA case as best prognosis and the same case as worst prognosis — Within-model consistency was generally good (CV<30%), except Gemini in the good-prognosis scenario (CV=39.15%). | Prompts: Partial Repro: Repeated (different day) RoB: High |
| S041 La Bella 2025 | La Bella 2025 Italy; Türkiye; Canada; Kenya; India doi: 10.1093/rheumatology/keaf329 | JIA | Patient education | Other/Unclear | ChatGPT-4o | Guideline-derived questions/treatments \| 10 items; 3 raters | ACR recommendations / expert validation | Consistency/reproducibility; Guideline concordance; Readability | Responses rated mostly or fully adherent to the 2021 ACR recommendations ranged from 26/50 (52%) to 42/50 (84%) across raters; no response was rated completely non-adherent. | Prompts: Yes Repro: Repeated (same protocol) RoB: Unclear |
| S042 Zhu 2025 | Zhu 2025 China doi: 10.1002/ksa.12639 | Osteoarthritis | Clinical decision support | Retrospective study | ChatGPT-4o | EHR / medical records \| 117 items; 117 participants; 3 raters | Orthopaedic surgeons | Accuracy; Classification performance; Reliability/agreement | ChatGPT-4o grading accuracy at two runs was 33.3%/32.5% (Kellgren–Lawrence), 42.7%/47.9% (Ahlbäck), and 23.1%/33.3% (IKDC), versus >80% for surgeons — ICCs were 0.28, 0.19, and 0.16, respectively. | Prompts: Yes Repro: Repeated (different day) RoB: Not applicable |
| S043 Ma 2025 | Ma 2025 China doi: 10.1038/s41598-025-02601-y | Disease not specified | Clinical decision support | Comparative evaluation | ChatGPT-4.0; Claude 3.5 Sonnet; Gemini 1.5 Pro | Guideline-derived questions/treatments \| 65 items; 8 raters | Human experts/clinicians; between-model comparison | Accuracy; Quality/completeness/usefulness; Readability; Safety/misinformation | Report-interpretation accuracy: Claude 3.5 Sonnet 100%, Gemini 1.5 Pro 97%, ChatGPT-4.0 90%, senior doctors 87%, junior doctors 67–73% — Overall 10-point ratings: ChatGPT-4.0 9.15 ± 0.48, Claude 3.5 Sonnet 9.15 ± 0.42, Gemini 1.5 Pro 9.03 ± 0.26. | Prompts: Partial Repro: None/not stated RoB: Unclear |
| S044 Aizawa 2024 | Aizawa 2024 Japan doi: 10.1093/mr/road115 | SAPHO | Clinical decision support | Comparative evaluation | ChatGPT-4.0 | EHR / medical records \| 151 patient records | Human experts/clinicians | Accuracy | English reports: sensitivity 83.5%, specificity 69.4%, accuracy 76.8% — Japanese reports: sensitivity 13.9%, specificity 98.6%, accuracy 54.3%; English reports without skin findings: accuracy 72.2%. | Prompts: Partial Repro: None/not stated RoB: Not applicable |
| S045 Bürgisser 2024 | Bürgisser 2024 Switzerland doi: 10.1136/rmdopen-2024-005003 | Mixed diseases | Clinical decision support | Comparative evaluation | Meta-LLaMA-3-8B | EHR / medical records \| 757 items; 1500 records | regex based natural language processing | Accuracy | Gout extraction: LLaMA PPV 92.7%, NPV 96.6%, accuracy 95.4% vs regex 92.3% for each metric — CPPD extraction: PPV 92.3%, NPV 95.9%, accuracy 94.1%. | Prompts: Yes Repro: Repeated (same protocol) RoB: Not applicable |
| S046 Cao 2025 | Cao 2025 China; United States; USA doi: 10.1016/j.jshs.2024.101016 | Osteoarthritis | Patient education | Comparative evaluation | ChatGPT-3.5; ChatGPT-4.0; Perplexity | Patient questions / online queries \| 25 items | Between-model comparison | Accuracy; Quality/completeness/usefulness | Mean quality score: ChatGPT-4.0 3.61 ± 0.57, ChatGPT-3.5 3.29 ± 0.77, Perplexity 3.11 ± 0.67 (p=0.038 and p<0.001 vs ChatGPT-4.0) — Mean comprehensiveness score: 4.56 ± 0.53, 3.96 ± 0.74, and 3.88 ± 0.85, respectively (ChatGPT-4.0 vs ChatGPT-3.5 p<0.001). | Prompts: Yes Repro: None/not stated RoB: Unclear |
| S047 Bayala 2025 | Bayala 2025 Burkina Faso doi: 10.1186/s41927-025-00512-z | Disease not specified | Clinical decision support | Comparative evaluation | ChatGPT-4.0; Copilot; Gemini 2.0 Flash; Claude 3.5 Sonnet | EHR / medical records \| 103 participants | Human experts/clinicians; between-model comparison | Accuracy | Accuracy using clinical data only: ChatGPT-4.0 77.66%, Claude 76.69%, Copilot 56.31%, Gemini 54.36% — With clinical plus paraclinical data: 86.41%, 85.44%, 75.73%, and 71.84%, respectively (p<0.001). | Prompts: Yes Repro: Repeated (different day) RoB: Not applicable |
| S048 Yoo 2025 | Yoo 2025 Korea doi: 10.1177/14604582251315587 | Osteoarthritis | Patient education | Other/Unclear | ChatGPT-3.5 | Patient questions / online queries \| 30 items | Prompt variants (detailed vs simplified instructions) | Quality/completeness/usefulness; Readability | Baseline readability: FKGL 13.65 ± 1.80 and SMOG 15.62 ± 1.55; all outputs exceeded grade 6 (p<0.001) — After detailed/simplified prompts, FKGL improved to 10.56/10.12 and SMOG to 13.04/12.40, but no output reached grade 6. | Prompts: Yes Repro: None/not stated RoB: Unclear |
| S049 Li 2024 | Li 2024 China; USA doi: 10.1136/bmjopen-2023-082344 | Osteoarthritis | Clinical decision support | Other/Unclear | GPT-4 | Guideline-derived questions/treatments | Human experts/clinicians | Accuracy; Guideline concordance | AAOS comparison: 13/28 (46.4%) correct, 50.0% largely correct, 3.6% incorrect; mean accuracy 4.3 ± 1.6 and completeness 2.8 ± 0.6 — In 50 case scenarios, comprehensive responses were obtained in 88–90% across radiology, diagnosis, and treatment. | Prompts: Partial Repro: None/not stated RoB: Unclear |
| S050 Gürses 2025 | Gürses 2025 Türkiye doi: 10.1007/s10916-025-02207-x | Osteoarthritis | Clinical decision support | Other/Unclear | ChatGPT-4o; Gemini Advanced | Guideline-derived questions/treatments \| 40 participants | Between-model comparison | Guideline concordance | Agreement with physiotherapist consensus was 74% for ChatGPT-4o (13/50 discrepancies) and 70% for Gemini Advanced (15/50 discrepancies). | Prompts: Yes Repro: None/not stated RoB: Not applicable |
| S051 Yang 2025 | Yang 2025 China; USA doi: 10.1097/JS9.0000000000002494 | Osteoarthritis | Patient education | Other/Unclear | ChatGPT-4.0 | Other/Not specified \| 60 items; 60 participants | Human experts/clinicians | Accuracy; Quality/completeness/usefulness | Answer quality: supervised ChatGPT 92.1 ± 4.3 vs free ChatGPT 81.4 ± 10.4 (p=0.001) — Knowledge score: supervised ChatGPT 95.3 ± 4.7 vs physician education 95.6 ± 5.3 (p=0.879); free ChatGPT 82.1 ± 12.3 (p<0.001). | Prompts: Partial Repro: None/not stated RoB: High |
| S052 Pratama MZ 2026 | Pratama MZ 2026 Indonesia doi: 10.1111/1756-185x.70713 | SLE | Clinical decision support | Comparative evaluation | ChatGPT-4.0; Gemini; DeepSeek; Claude Sonnet 4 | EHR / medical records \| 100 participants; 100 records | Final diagnosis established by the attending rheumatologist; between-model comparison | Accuracy; Classification performance | Accuracy: ChatGPT-4 93% (95% CI 88.4–97.7%) — Accuracy: Gemini 96% (95% CI 91.2–100.0%). | Prompts: Partial Repro: None/not stated RoB: Not applicable |
| S053 Kim JH 2026 | Kim JH 2026 South Korea doi: 10.4258/hir.2026.32.2.125 | Mixed diseases | Patient education | Comparative evaluation | Mi:dm-2.0-mini-Instruct; EXAONE-3.5-2.4B-Instruct; Kanana-1.5-2.1B-Instruct-2505; HyperCLOVAX-SEED-Text-Instruct-1.5B; LLaMA-3.2-3B-Instruct | Patient questions / online queries \| 597 items; 1 rater | Reference answers from AI Hub; base-model performance; between-model comparison | Accuracy; Quality/completeness/usefulness; Safety/misinformation | BERTScore-F1 after vs before fine-tuning: Mi:dm-2.0-mini-Instruct 0.7290 vs 0.6900 — EXAONE-3.5-2.4B-Instruct 0.7390 vs 0.7020. | Prompts: No/Unclear Repro: None/not stated RoB: Not applicable |
| S054 Kerkhof P 2026 | Kerkhof P 2026 Netherlands; USA; UK doi: 10.1007/s00296-026-06128-x | Myositis | Text analysis / information extraction | Cross-sectional study | GPT-5.1 API | Patient questions / online queries \| 200 items; 968 participants; 1,529 records; 2 raters | Manual coding by two independent coders | Accuracy; Classification performance | Accuracy: GPT-5.1 API 92% — Precision: GPT-5.1 API 0.93. | Prompts: Yes Repro: Repeated (same protocol) RoB: Not applicable |
| S055 Honda S 2026 | Honda S 2026 Japan doi: 10.1093/mr/roag039 | Rheumatoid arthritis | Clinical decision support | Cross-sectional study | Meditron 70B; fine-tuned LLaMA 2; fine-tuned Meditron | EHR / medical records \| 8 items; 11,865 participants; 11,865 records | No direct comparator; logistic regression; random forest; XGBoost; base and fine-tuned LLaMA 2 and Meditron | Accuracy; Classification performance; Disease activity; Patient-reported outcomes | DAS28-ESR formula accuracy: Meditron 70B 60.0% (3/5) vs LLaMA 2 70B 0 — DAS28-ESR interpretation accuracy: Meditron 70B 100.0% (5/5) vs LLaMA 2 70B 0. | Prompts: Partial Repro: Repeated (same protocol) RoB: Not applicable |
| S056 Walker A 2026 | Walker A 2026 USA doi: 10.1002/acr.25687 | SLE | Text analysis / information extraction | Cross-sectional study | Instruction-fine-tuned LLaMA 3.2 Instruct 3B | Patient questions / online queries \| 10,857 participants; 31,785 records; 2 raters | Human-annotated structured summaries; topic-modeling output | Accuracy; Quality/completeness/usefulness; Reliability/agreement; Safety/misinformation | Structured summaries meeting predefined criteria: 90/100 (90%) — Mean thematic accuracy: 3.11/4; hallucination rate: 8%. | Prompts: Partial Repro: None/not stated RoB: High |
| S057 Moon P 2026 | Moon P 2026 United Kingdom doi: 10.1016/j.ymeth.2025.10.010 | Psoriatic arthritis | Clinical decision support | Comparative evaluation | Qwen-2.5-7B; Qwen-2.5-32B; Qwen-2.5-72B; Qwen/LGBM hybrids | EHR / medical records \| 32 participants; 32 records | Between-model comparison | Disease activity | PsA flare prediction: Sensitivity: Qwen-2.5–7B 0.71 vs LGBM using structured data 0.81 — PsA flare prediction: Specificity: Qwen-2.5–7B 0.38 vs LGBM using structured data 0.62. | Prompts: No/Unclear Repro: None/not stated RoB: Not applicable |
| S058 Kremer P 2025 | Kremer P 2025 Germany doi: 10.1016/j.ero.2025.04.007 | Mixed diseases | Clinical decision support | Comparative evaluation | Gemini 1.5 Pro; LLaMA 3.3 70B; ChatGPT-4o; Claude 3.5 Sonnet | Clinical vignettes / case scenarios \| 60 items; 3 raters | Ada; Symptoma; Isabel DDx; between-model comparison | Accuracy; Efficiency/productivity | Identical top-1 diagnosis: Claude 3.5 Sonnet 40.0%, ChatGPT-4o 38.3%, Gemini 1.5 Pro 35.0%, LLaMA 3.3 26.7% — Traditional DDSS: Ada 15.0%, Isabel DDx 10.0%, Symptoma 1.7%. | Prompts: Yes Repro: None/not stated RoB: Unclear |
| S059 Madrid-García A 2025 | Madrid-García A 2025 Spain doi: 10.1016/j.ero.2025.08.001 | Disease not specified | Clinical decision support | Comparative evaluation | o3-mini with RAG | Clinical vignettes / case scenarios \| 740 items; 74 articles; 2 raters | o3-mini without RAG; full EULAR/ACR guideline context | Accuracy; Guideline concordance; Quality/completeness/usefulness; Safety/misinformation | Automatic LLM-as-a-judge: Factual accuracy, mean ± SD: o3-mini with RAG 4.9 ± 0.3 vs o3-mini without RAG 4.4 ± 0.7 (p<0.001) — Manual Factual accuracy, mean ± SD: o3-mini with RAG 4.4 ± 0.9 vs o3-mini without RAG 3.6 ± 1.1 (p<0.001). | Prompts: Yes Repro: None/not stated RoB: Unclear |
| S060 Shao Z 2026 | Shao Z 2026 China doi: 10.1007/s10067-026-08073-3 | Gout/crystal arthropathy | Patient education | Comparative evaluation | DeepSeek-V3; DeepSeek-R1; GPT-5 | Guideline-derived questions/treatments \| 42 items; 3 raters | ACR gout and hyperuricemia guideline content; between-model comparison | Accuracy; Readability; Reliability/agreement; Response length | Expert accuracy score: DeepSeek-V3 4.67 (4.00–5.00) (p<0.001) — Expert accuracy score: DeepSeek-R1 5.00 (4.33–5.00) (p<0.001). | Prompts: Yes Repro: None/not stated RoB: Unclear |
| S061 Atilan AU 2026 | Atilan AU 2026 Türkiye doi: 10.1016/j.ijmedinf.2025.106246 | Psoriatic arthritis | Patient education | Comparative evaluation | Gemini 2.0 Flash; Claude 3.7 Sonnet; ChatGPT-4o; Mistral Large 2; Grok 3; DeepSeek-R1; Qwen 2.5 | Guideline-derived questions/treatments \| 40 items; 14 records; 2 raters | Between-model comparison; DISCERN criteria | Readability; Reliability/agreement; Response length | Ateşman readability index: All seven LLMs 61.6–80.2; mean 71.3 ± 6.9 — Total DISCERN score: Claude 3.7 Sonnet 60.5/80 (75.6%) (Friedman p<0.0001). | Prompts: Yes Repro: None/not stated RoB: Unclear |
| S062 Polyzou M 2026 | Polyzou M 2026 Germany; Greece doi: 10.1186/s41927-026-00618-y | Mixed diseases | Clinical decision support | Comparative evaluation | ChatGPT-5; DeepSeek-R1 | EHR / medical records \| 28 items; 116 participants; 116 records | Clinical findings recorded in routine care; ChatGPT vs DeepSeek | Classification performance; Consistency/reproducibility | Agreement with clinical findings: ChatGPT-5 0.030 (p=0.086) — Agreement with clinical findings: DeepSeek-R1 -0.009 (p=0.595). | Prompts: Yes Repro: None/not stated RoB: Not applicable |
| S063 Bai J 2026 | Bai J 2026 China doi: 10.2196/79153 | Axial spondyloarthritis | Patient education | Comparative evaluation | GPT-4.0; DeepSeek-R1; Kimi k1.5; Hunyuan T1; Wenxin X1 | Patient questions / online queries \| 42 items; 110 participants; 1,052 records; 2 raters | Guideline-informed expert scoring; between-model comparison | Accuracy; Readability; Reliability/agreement; Safety/misinformation | Domain-level expert score: diagnosis/examination 20.4 ± 0.9 vs treatment/medication 19.3 ± 1.7 — Inter-rater reliability: 0.947. | Prompts: Yes Repro: None/not stated RoB: Unclear |
| S064 Bayala YLT 2026 | Bayala YLT 2026 Burkina Faso doi: 10.1177/14604582261448687 | Disease not specified | Clinical decision support | Comparative evaluation | ChatGPT-4; Claude 3.5 Sonnet; Gemini 1.5 Pro; DeepSeek-V3 | Scientific articles / case reports \| 9 items; 9 records; 9 articles; 2 raters | Published final diagnoses; between-model comparison | Accuracy; Consistency/reproducibility; Quality/completeness/usefulness; Safety/misinformation | Correct primary diagnosis: ChatGPT-4 9/9 (100%) — Correct primary diagnosis: Claude 3.5 Sonnet 9/9 (100%). | Prompts: Yes Repro: Repeated (different day) RoB: Unclear |
| S065 Fornaro M 2026 | Fornaro M 2026 Italy; United Kingdom; USA doi: 10.1007/s00296-026-06205-1 | Myositis | Clinical decision support | Comparative evaluation | Claude v3.5 Sonnet | Scientific articles / case reports \| 30 items; 30 records; 2 raters | Two expert rheumatologists | Disease activity; Efficiency/productivity | Agreement with expert rheumatologists: global CDASI activity ICC 0.71 (95% CI 0.45–0.86); global CDASI damage ICC 0.41 (95% CI 0.25–0.67) — Assessment time: 42 seconds vs 8.4 minutes for experts (92% reduction). | Prompts: Partial Repro: None/not stated RoB: Not applicable |
| S066 Yemeshev Y 2026 | Yemeshev Y 2026 Kazakhstan doi: 10.1007/s00296-026-06103-6 | Rheumatoid arthritis | Clinical decision support | Comparative evaluation | Gemini; GPT-5 Thinking; DeepSeek | Medical images \| 8 items; 20 records; 6 raters | Consensus radiographic assessment by radiologists | Accuracy; Classification performance; Reliability/agreement | Overall radiographic accuracy: Gemini 65% — Overall radiographic accuracy: GPT-5 Thinking 66%. | Prompts: Partial Repro: None/not stated RoB: Not applicable |
| S067 Du K 2026 | Du K 2026 China doi: 10.1016/j.ijmedinf.2026.106409 | Osteoarthritis | Patient education | Comparative evaluation | GPT-4o; Claude 3.5 Sonnet | EHR / medical records \| 150 items; 150 participants; 150 records; 3 raters | Personalized explanations written by two experienced orthopedic clinicians | Efficiency/productivity; Quality/completeness/usefulness; Readability | Expert-rated understandability: GPT-4o 72 (IQR 6) (p<0.001) — Expert-rated understandability: Claude 3.5 Sonnet 60 (IQR 6) (p<0.001). | Prompts: Partial Repro: None/not stated RoB: Unclear |
| S068 Widhani A 2026 | Widhani A 2026 Indonesia doi: 10.2196/89156 | SLE | Patient education | Comparative evaluation | DeepSeek-V3; Gemini 2.5 Flash; ChatGPT-4o | Patient questions / online queries \| 22 items; 5 participants; 66 records; 5 raters | Clinical immunologist ratings; between-model comparison | Accuracy; Readability; Reliability/agreement; Response length | Expert accuracy score: DeepSeek-V3 1.48 ± 0.63 (p<0.001) — Expert accuracy score: Gemini 2.5 Flash 1.25 ± 0.53 (p<0.001). | Prompts: Yes Repro: None/not stated RoB: Unclear |
| S069 Munguía-Realpozo P 2026 | Munguía-Realpozo P 2026 Mexico doi: 10.1177/20552076261467480 | Disease not specified | Research support / evidence synthesis | Comparative evaluation | Systematic Review Extractor Pro; GPT-Reviewer | Scientific articles / case reports \| 34 records; 34 articles | Human-adjudicated extraction and QUIPS assessments | Accuracy; Efficiency/productivity; Reliability/agreement | Data-extraction concordance: Age: Systematic Review Extractor Pro 0/15 (0%) — Data-extraction concordance: Study design: Systematic Review Extractor Pro 11/15 (73.3%). | Prompts: Yes Repro: None/not stated RoB: Unclear |
| S070 Choi SR 2026 | Choi SR 2026 South Korea doi: 10.1186/s12911-026-03467-4 | Rheumatoid arthritis | Conversational agents / patient-facing interaction | Prospective study | RA shared-decision chatbot | Survey / questionnaire \| 20 participants; 20 records; 20 raters | Phase 1 vs phase 2; pre- vs post-chatbot decisional conflict | Attitudes/trust; Patient-reported outcomes; Quality/completeness/usefulness; Usability/acceptability | Usefulness: RA shared-decision chatbot 3.7 vs Phase 1 3.7 (Not significant) — System Usability Scale: RA shared-decision chatbot 70.0 ± 10.6 vs Phase 1 66.0 ± 11.4 (p=0.440). | Prompts: No/Unclear Repro: None/not stated RoB: Unclear |
| S071 Kremer P 2026 | Kremer P 2026 Germany; Norway doi: 10.1007/s00296-025-06068-y | Mixed diseases | Clinical decision support | Comparative evaluation | OpenEvidencee; ChatGPT-5 Thinking; Prof. Valmed | Clinical vignettes / case scenarios \| 60 items; 60 records; 50 articles; 3 raters | Blinded rheumatologist assessment; between-model comparison | Accuracy; Efficiency/productivity; Reliability/agreement | Identical top-1 diagnosis: OpenEvidence 35.0% (Cochran Q p=0.001; no significant pairwise differences after Holm correction) — Identical diagnosis within the top five: 56.7%. | Prompts: Yes Repro: None/not stated RoB: Unclear |
| S072 Ohlsen SM 2026 | Ohlsen SM 2026 USA doi: 10.1016/j.xrrt.2026.100715 | Osteoarthritis | Patient education | Comparative evaluation | ChatGPT; Gemini | Guideline-derived questions/treatments \| 12 items; 24 records; 2 raters | AAOS glenohumeral osteoarthritis clinical practice guideline | Guideline concordance; Reliability/agreement; Safety/misinformation | Agreement with AAOS recommendation: ChatGPT 6/12 (50%) (p=0.20 for overall distribution) — Neutral/uncertain response: ChatGPT 1/12 (8%). | Prompts: Yes Repro: None/not stated RoB: Unclear |
| S073 Lalitkanjanakul T 2026 | Lalitkanjanakul T 2026 Thailand doi: 10.1177/20420986261460173 | Rheumatoid arthritis | Clinical decision support | Retrospective study | Gemini 2.5 Pro | EHR / medical records \| 259 participants; 259 records; 1 rater | Micromedex; WebMD; real-world co-prescription; expert rheumatologist | Quality/completeness/usefulness; Reliability/agreement; Safety/misinformation | Gemini 2.5 Pro showed the closest alignment with expert assessment of drug–drug interaction relevance — Agreement between Micromedex and WebMD: Brennan–Prediger κ=0.3161; for preselected serious interactions: κ=0.9807. | Prompts: Yes Repro: None/not stated RoB: Unclear |
| S074 Ji X 2026 | Ji X 2026 China doi: 10.1038/s41746-026-02372-4 | Axial spondyloarthritis | Clinical decision support | Validation study | SpAgents; SpAgents-assisted diagnosis | EHR / medical records \| 596 participants; 596 records | Primary care physicians; junior and senior rheumatologists | Accuracy; Classification performance | Diagnostic accuracy: 0.8444 in the validation cohort and 0.8627 in the independent test cohort — Corresponding F1 scores: 0.8889 and 0.8955. | Prompts: Yes Repro: None/not stated RoB: Not applicable |
| S075 Shah N 2026 | Shah N 2026 USA doi: 10.1002/acr.80109 | Systemic sclerosis | Conversational agents / patient-facing interaction | Prospective study | Sprout AI health coach | Survey / questionnaire \| 20 participants; 20 records; 20 raters | High- vs low-engagement participant groups | Patient-reported outcomes; Usability/acceptability | Sessions: Sprout AI health coach 21.0 vs Low-engagement group 7.5 — Total chat duration (seconds): Sprout AI health coach 5861.3 vs Low-engagement group 1193.6. | Prompts: No/Unclear Repro: None/not stated RoB: Not applicable |
| S076 Mirza Y 2026 | Mirza Y 2026 Türkiye doi: 10.1111/1756-185x.70676 | Rheumatoid arthritis | Patient education | Comparative evaluation | DeepSeek-R1; Gemini 2.5; ChatGPT-5 | Patient questions / online queries \| 46 items; 138 records; 2 raters | Between-model comparison | Accuracy; Readability; Reliability/agreement | Accuracy score: DeepSeek-R1 4 (4–4) (p<0.001) — Accuracy score: Gemini 2.5 3 (3–4) (p<0.001). | Prompts: Yes Repro: None/not stated RoB: Unclear |
| S077 Schütze K 2026 | Schütze K 2026 Australia doi: 10.1016/j.nmd.2026.106418 | Myositis | Patient education | Comparative evaluation | ChatGPT | Patient questions / online queries \| 6 items; 6 records; 12 raters | Expert physiotherapist ratings | Accuracy; Safety/misinformation | Overall safe/mostly accurate questions: ChatGPT 5/6 — Expert rating distribution for question 1: ChatGPT 66.7% comprehensive/accurate; 25.0% safe but incomplete; 8.3% other. | Prompts: Yes Repro: None/not stated RoB: Unclear |
| S078 Lilly M 2026 | Lilly M 2026 USA doi: 10.7759/cureus.106160 | Mixed diseases | Patient education | Comparative evaluation | ChatGPT; Google Gemini; OpenEvidence | Patient questions / online queries \| 9 items; 189 records; 8 raters | Expert rheumatology ratings; between-model comparison | Accuracy; Consistency/reproducibility; Readability; Response length | Expert votes for best RA response: ChatGPT 1, Gemini 1, OpenEvidence 6 — For OA: ChatGPT 2, Gemini 3, OpenEvidence 3. | Prompts: Yes Repro: Repeated (different day) RoB: Unclear |
| S079 Hassan F 2026 | Hassan F 2026 Israel doi: Not reported | Disease not specified | Medical education | Comparative evaluation | GPT-4-turbo (English/Hebrew base prompts, few-shot chain-of-thought, and knowledge-augmented configurations) | Exam questions (MCQ/images) \| 200 items; 200 records | Official Israeli rheumatology board answer key; prompting variants | Accuracy | Overall examination accuracy: English base prompt 77.0% (no significant overall difference among configurations) — Text-only accuracy: 82.5%; image-based accuracy: 34.8%. | Prompts: No/Unclear Repro: None/not stated RoB: Not applicable |
| S080 Meral HB 2026 | Meral HB 2026 Türkiye doi: 10.1038/s41598-026-35166-5 | Gout/crystal arthropathy | Clinical decision support | Comparative evaluation | ChatGPT-4o; Gemini 2.0 Flash | Guideline-derived questions/treatments \| 25 items; 50 records; 2 raters | 2016/2018 EULAR gout recommendations; between-model comparison | Guideline concordance; Quality/completeness/usefulness; Readability; Reliability/agreement; Safety/misinformation | Guideline-alignment score: ChatGPT-4o 4 (2–4) (p<0.001) — Guideline-alignment score: Gemini 2.0 Flash 3 (1–4) (p<0.001). | Prompts: Yes Repro: None/not stated RoB: Unclear |
| S081 Rafiei F 2026 | Rafiei F 2026 Iran doi: 10.1038/s41598-026-38716-z | Disease not specified | Medical education | Comparative evaluation | GPT-4o; GPT-5.1 | Exam questions (MCQ/images) \| 204 items; 204 records; 6 raters | Official Iranian rheumatology board answer key; between-model comparison | Accuracy; Consistency/reproducibility; Quality/completeness/usefulness; Reliability/agreement | Board-examination accuracy: GPT-4o 64.5% (McNemar p=0.002) — Board-examination accuracy: GPT-5.1 76.0% (McNemar p=0.002). | Prompts: Partial Repro: None/not stated RoB: Unclear |
| S082 Wang Y 2026 | Wang Y 2026 China doi: 10.3389/fmed.2025.1716122 | Disease not specified | Medical education | Comparative evaluation | DeepSeek-R1; DeepSeek-R1-32B; DeepSeek-V3Pro; GPT-4.1; GPT-4o; LLaMA 4 Scout; Gemma 2-27B; Gemma 3-27B; GLM-4-32B; Qwen 3-235B; Qwen 3-32B | Exam questions (MCQ/images) \| 2,829 items; 2,829 records | Official examination answer key; between-model comparison | Accuracy; Consistency/reproducibility | Total examination accuracy: DeepSeek-R1 91.91% — Total examination accuracy: DeepSeek-R1–32B 86.78%. | Prompts: No/Unclear Repro: Repeated (same protocol) RoB: Not applicable |
| S083 Öner SK 2026 | Öner SK 2026 Türkiye doi: 10.5152/j.aott.2026.25618 | Osteoarthritis | Patient education | Comparative evaluation | Gonarthrosis Advisor; ChatGPT-5 | Patient questions / online queries \| 30 items; 60 records; 2 raters | Between-model comparison | Readability; Reliability/agreement | Flesch-Kincaid Grade Level: Gonarthrosis Advisor 7.8 ± 0.7 vs ChatGPT-5 9.6 ± 0.9 (p<0.001) — Flesch Reading Ease: Gonarthrosis Advisor 54.3 ± 3.4 vs ChatGPT-5 46.7 ± 3.7 (p<0.001). | Prompts: Yes Repro: None/not stated RoB: Unclear |
| S084 Roemer A 2026 | Roemer A 2026 Germany doi: 10.1186/s12909-026-09079-w | Disease not specified | Medical education | Prospective study | ChatGPT-4o (standalone and clinician-assisted) | Clinical vignettes / case scenarios \| 3 items; 68 participants; 204 records | Conventional resources without ChatGPT-4o | Accuracy; Attitudes/trust; Efficiency/productivity | Correct top diagnosis: ChatGPT-4o-assisted group 77.5% vs conventional resources 32.4% (adjusted OR 7.0, 95% CI 3.8–14.4; p<0.001) — ChatGPT-4o alone: 71.6%. | Prompts: Yes Repro: None/not stated RoB: Low |
| S085 Felde S 2026 | Felde S 2026 Germany doi: 10.3389/fmed.2026.1817215 | Disease not specified | Clinical decision support | Comparative evaluation | Mixtral-8×7B-32768; LLaMA 3.1-Nemotron-70B; Qwen-Turbo 2.5; Claude-3.5-Sonnet; GPT-4o | Clinical vignettes / case scenarios \| 10 items; 200 records; 2 raters | Expert reference; five models under four RAG/pre-diagnosis configurations | Classification performance; Quality/completeness/usefulness; Reliability/agreement; Safety/misinformation | Diagnostic F1: Mixtral-8×7B-32768 72% — Therapeutic F1: Mixtral-8×7B-32768 73%. | Prompts: Partial Repro: None/not stated RoB: Unclear |
| S086 Venerito V 2026 | Venerito V 2026 International/multinational (GRAPPA) doi: 10.1007/s11739-026-04396-w | Psoriatic arthritis | Text analysis / information extraction | Cross-sectional survey | Meta-LLaMA 3.2 3B Instruct | Survey questionnaire \| 4 items; 570 participants; 234 records; 2 raters | Human-gold consensus from two board-certified rheumatologists | Accuracy; Other; Reliability/agreement | LLM agreement with human-gold consensus: Meta-LLaMA 3.2 3B Instruct κ=0.78 (95% CI 0.70–0.85) — Human inter-rater agreement: κ=0.81 (95% CI 0.74–0.87). | Prompts: Yes Repro: None/not stated RoB: Not applicable |
| S087 Akyuz B 2026 | Akyuz B 2026 Türkiye doi: 10.1080/17538157.2026.2654150 | Mixed diseases | Patient education | Comparative evaluation | GPT-4o; DeepSeek-V3 | Patient questions / online queries \| 16 items; 90 participants; 32 records; 30 physician and 60 patient raters | Between-model comparison | Accuracy; Attitudes/trust; Efficiency/productivity; Quality/completeness/usefulness; Readability; Reliability/agreement | Scientific accuracy: GPT-4o 4.11 vs DeepSeek-V3 3.84; mean difference 0.275 (95% CI 0.13–0.42; p<0.001) — Treatment/drug-selection difference: 0.287 (95% CI 0.11–0.46; p=0.002; Holm-adjusted p=0.013). | Prompts: Partial Repro: None/not stated RoB: Unclear |
| S088 Ulutaş F 2026 | Ulutaş F 2026 Türkiye doi: 10.3390/diagnostics16010113 | Mixed diseases | Clinical decision support | Comparative evaluation | ChatGPT-4o | Clinical vignettes / case scenarios \| 6 items; 47 participants; 47 records; 3 blinded reviewers; multidisciplinary team of 10 specialists | Multidisciplinary team consensus decisions | Reliability/agreement | Clinical-diagnosis concordance with MDT consensus: 31/47 (65.96%), κ=0.52 (p<0.001) — Radiological-diagnosis concordance: 30/47 (63.83%), κ=0.55 (p<0.001). | Prompts: Yes Repro: None/not stated RoB: Unclear |
| S089 Ji X 2026 | Ji X 2026 China doi: 10.20517/ais.2025.93 | Mixed diseases | Clinical decision support | Comparative evaluation | Baichuan-13B; LLaMA 2-7B-Chat; Qwen-7B-Chat; ChatGLM2-6B-32K; SpAD-LLM-10B; SpAD-LLM-34B; Huatuo2-13B; LLaMA 2-70B-Chat; Falcon-40B; Meditron-70B; Mistral-7B; Orca2-13B; Yi-34B; GPT-4 | Exam questions (MCQ/images) \| 222 items; 100 records; 2 raters | GPT-4 and public/open-source baseline models; before-versus-after domain-specific fine-tuning | Accuracy; Quality/completeness/usefulness; Reliability/agreement | Overall SpAMCQA accuracy after vs before fine-tuning: Baichuan-13B 79.81% vs 62.54% — LLaMA 2-7B-Chat 62.37% vs 34.02%. | Prompts: Partial Repro: None/not stated RoB: Unclear |
| S090 van der Weegen W 2026 | van der Weegen W 2026 Netherlands doi: 10.1016/j.pecinn.2025.100446 | Osteoarthritis | Conversational agents / patient-facing interaction | Prospective study | ChatGPT-3.5-supported virtual assistant | Survey questionnaire \| 18 items; 40 participants; 40 records; 40 raters | No control group; modified ASAQ benchmark dimensions | Attitudes/trust; Efficiency/productivity; Empathy; Safety/misinformation; Usability/acceptability | Generated-history summaries contained 22 hallucinations in 16/40 patients (1–4 per affected summary) — 87.5% of participants agreed that the virtual assistant behaved like a human. | Prompts: No/Unclear Repro: None/not stated RoB: High |
| S091 van 't Klooster JWJR 2026 | van 't Klooster JWJR 2026 Netherlands; Italy doi: 10.3389/fdgth.2025.1653168 | Osteoarthritis | Conversational agents / patient-facing interaction | Prospective study | GPT-3.5-reinforced Furhat social robot; GPT-4o-reinforced Furhat social robot | Patient questions / online queries \| 48 participants; 48 records; 48 raters | UEQ benchmarks; patients versus healthcare professionals | Attitudes/trust; Usability/acceptability | Pilot UEQ scores: attractiveness 1.50 and perspicuity 1.63 — Clinical-practice overall UEQ score: 2.13 among patients and 1.89 among healthcare professionals. | Prompts: Yes Repro: None/not stated RoB: Unclear |
| S092 Güngör Demir U 2026 | Güngör Demir U 2026 Türkiye doi: 10.5152/ArchRheumatol.2026.25254 | Axial spondyloarthritis | Clinical decision support | Retrospective study | ChatGPT | EHR / medical records \| 530 items; 266 participants; 266 records; 3 raters | Consensus modified New York grades from two experts with third-reviewer adjudication | Accuracy; Classification performance; Reliability/agreement | Five-grade classification accuracy: ChatGPT 30.4% — Agreement with expert grading was slight (κ=0.136, p<0.001). | Prompts: Yes Repro: None/not stated RoB: Not applicable |
| S093 Pan X 2026 | Pan X 2026 China doi: 10.3389/fmed.2026.1758735 | Gout/crystal arthropathy | Medical education | Comparative evaluation | DeepSeek-R1; ChatGPT-4.0 | Patient questions / online queries \| 15 items; 25 participants; 30 records; 2 raters | Between-model comparison | Other; Quality/completeness/usefulness; Readability; Reliability/agreement; Safety/misinformation | Flesch-Kincaid Grade Level: DeepSeek-R1 13.04 ± 1.62 vs ChatGPT-4.0 11.41 ± 1.74 (p=0.013) — Flesch Reading Ease: DeepSeek-R1 40.50 ± 8.12 vs ChatGPT-4.0 49.08 ± 8.90 (p=0.010). | Prompts: Yes Repro: None/not stated RoB: Unclear |

Prompt reporting: Yes = all prompts and items reproduced verbatim in the article or supplement; Partial = only part reproduced; No/Unclear = no verbatim wording available. Reproducibility refers to repeated querying of the model to assess answer stability.

RoB = overall risk of bias according to the Alberta Quality Assessment Tool: Risk of Bias, using the worst-item rule. “Not applicable” indicates study designs outside the scope of the tool, that is, studies without human evaluation of free-text model answers.

ACR, American College of Rheumatology; axSpA, axial spondyloarthritis; CPPD, calcium pyrophosphate deposition disease; EHR, electronic health record; FMF, familial Mediterranean fever; GIOP, glucocorticoid-induced osteoporosis; JIA, juvenile idiopathic arthritis; LLM, large language model; OA, osteoarthritis; RA, rheumatoid arthritis; RoB, risk of bias; SLE, systemic lupus erythematosus.

Supplementary Table 2. Study-level values and reasons for exclusion in the quantitative outcome synthesis

*One value was taken per study. Where several models were evaluated, the best-performing model was used for accuracy and guideline concordance and the median across models for readability. Study identifiers refer to Supplementary Table 1.*

**A Accuracy in clinical decision support (38 studies in the domain)**

**A1 Included in the synthesis (n = 17)**

| **Study** | **Best-performing model** | **Metric used for the synthesis** | **Accuracy (%)** |
| --- | --- | --- | --- |
| **S002** | GPT-4 | Overall diagnostic accuracy, records plus immunology | 97.0 |
| **S004** | GPT-4 | Top-1 diagnostic accuracy, FMF vignettes | 50.0 |
| **S009** | ChatGPT-4 | Correct multiple-choice responses, day 14 | 90.0 |
| **S024** | GPT-4o | Hit@1, few-shot prompting | 85.9 |
| **S026** | ChatGPT-4 | Correct top-1 diagnosis | 35.0 |
| **S043** | Claude 3.5 Sonnet | Accuracy, report interpretation | 100.0 |
| **S044** | ChatGPT-4.0 | Accuracy, English scintigraphy reports | 76.8 |
| **S045** | Meta-Llama-3-8B | Accuracy, gout detection in electronic health records | 95.4 |
| **S047** | ChatGPT-4.0 | Accuracy, clinical plus paraclinical data | 86.4 |
| **S052** | Gemini | Accuracy, SLE versus mimicking conditions | 96.0 |
| **S058** | Claude 3.5 Sonnet | Identical top-1 diagnosis, rare diseases | 40.0 |
| **S064** | ChatGPT-4 / Claude 3.5 Sonnet / Gemini 1.5 Pro | Correct primary diagnosis (9 cases) | 100.0 |
| **S066** | GPT-5 Thinking | Overall radiographic accuracy | 66.0 |
| **S071** | OpenEvidence | Identical top-1 diagnosis | 35.0 |
| **S074** | SpAgents (multi-agent system) | Diagnostic accuracy, validation cohort | 86.3 |
| **S089** | SpAD-LLM-34B (domain-fine-tuned) | Overall benchmark accuracy after fine-tuning | 92.4 |
| **S092** | ChatGPT-5o | Overall five-grade sacroiliitis classification | 30.4 |

S004 (Pillai 2023) evaluated two diseases in separate vignette sets. Section A1 reports the top-1 accuracy of the best-performing model (GPT-4) in familial Mediterranean fever (50.0 %), which was the single value carried forward into the pooled median in accordance with the pre-specified one-value-per-study rule. Section D and Supplementary Table 1 report the head-to-head comparison for deficiency of the interleukin-1 receptor antagonist, in which GPT-4 achieved 0 % versus 35 % for the internist (p = 0.0083). Both values are correct and refer to the same study but to different disease cohorts.

**A2 Reported an accuracy or classification outcome but not eligible for a proportion-based summary (n = 9)**

| **Study** | **Reason for exclusion** |
| --- | --- |
| **S011** | Correctness reported only as a 5-point Likert rating (4.28 ± 0.44) |
| **S025** | No overall accuracy reported; only sensitivity, specificity and predictive values |
| **S040** | Accuracy reported only qualitatively as concordance of prognostic ranking across systems |
| **S042** | Overall accuracy reported for the human comparator only; model results given as precision, recall and F1 per grading system |
| **S049** | Accuracy reported only as 5-point Likert mean scores |
| **S055** | Accuracy reported only per individual score formula with denominators of five items |
| **S059** | Factual accuracy reported only on a 5-point Likert scale |
| **S062** | Agreement with clinical findings reported only as a kappa coefficient |
| **S085** | Performance reported only as diagnostic and therapeutic F1 scores |

**A3 No accuracy or classification outcome reported and therefore not eligible (n = 12)**

**Studies**

**S003, S005, S007, S019, S034, S037, S050, S057, S065, S073, S080, S088**

**B Readability of patient-facing output, Flesch-Kincaid Grade Level (33 studies assessed readability)**

**B1 Included in the synthesis (n = 13)**

| **Study** | **Model(s)** | **Output evaluated** | **FKGL (grade)** |
| --- | --- | --- | --- |
| **S001** | ChatGPT-3.5 | Rewritten ACR patient education materials | 8.29 |
| **S012** | ChatGPT-4o | Ankylosing spondylitis frequently asked questions | 15.33 |
| **S013** | ChatGPT | Plain-language summaries of rheumatology articles | 10.52 |
| **S023** | GPT-4 | Personalised knee osteoarthritis self-management guidance | 11.56 |
| **S027** | ChatGPT | Spondyloarthropathy patient texts | 12.35 |
| **S029** | ChatGPT-4o | Information on biologic therapy in SLE | 11.70 |
| **S030** | ChatGPT-4 | Rheumatic disease patient education texts | 12.57 |
| **S037** | ChatGPT-3.5 and GPT-4.0 | axSpA management recommendations (median of two models) | 17.45 |
| **S048** | ChatGPT-3.5 | Knee osteoarthritis questions, pre-intervention | 13.65 |
| **S060** | DeepSeek-V3, DeepSeek-R1 and GPT-5 | Gout management responses (median of three models) | 11.79 |
| **S080** | ChatGPT-4o and Gemini 2.0 Flash | Gout guideline-derived questions (median of two models) | 14.53 |
| **S083** | Gonarthrosis Advisor | Purpose-built custom model for knee osteoarthritis | 7.80 |
| **S093** | DeepSeek-R1 and ChatGPT-4.0 | Gout nursing care questions (median of two models) | 12.23 |

**B2 Reported a grade-level metric but not eligible (n = 3)**

| **Study** | **Reason for exclusion** |
| --- | --- |
| **S014** | Reported as "grade level difference" without specifying the underlying index |
| **S036** | Flesch Reading Ease reported qualitatively only ("very difficult, university level"); no numerical grade level |
| **S078** | Hemingway grade level reported, which is not the Flesch-Kincaid index |

**B3 Readability assessed with other indices only and therefore not eligible (n = 17)**

| **Studies** | **Indices used instead** |
| --- | --- |
| **S006, S008, S028** | Word count or qualitative readability rating only |
| **S010, S018, S032, S041, S068, S076** | Flesch Reading Ease only |
| **S016, S061, S063** | Language-specific formulas only (Flesch Italian, Gulpease, Ateşman, Chinese-language index) |
| **S067** | Automated Readability Index, Coleman-Liau Index and Gunning Fog Index only |
| **S020, S043, S087** | Readability rated on Likert scales by physicians or patients only |
| **S035** | Multiple indices reported without a numerical Flesch-Kincaid grade level |

**C Guideline concordance (12 studies reported a guideline-concordance outcome)**

**C1 Included in the synthesis (n = 10)**

| **Study** | **Best-performing model** | **Metric used for the synthesis** | **Concordance (%)** |
| --- | --- | --- | --- |
| **S003** | ChatGPT | Treatment concordance with AAOS clinical practice guideline, hip and knee osteoarthritis | 80.0 |
| **S006** | ChatGPT-4 | Good answer rate against the 2022 ACR guideline on glucocorticoid-induced osteoporosis | 64.0 |
| **S007** | ChatGPT-4o | Fully aligned, complete and accurate responses, SHARE/EULAR recommendations for FMF | 80.6 |
| **S009** | ChatGPT-4 | Guideline-congruent responses at baseline, EULAR recommendations for rheumatoid arthritis | 96.0 |
| **S019** | DeepSeek-R1 | Concordant recommendations, Chinese knee osteoarthritis guidelines | 71.0 |
| **S034** | ChatGPT-4.0 | Guideline concordance, AAOS glenohumeral osteoarthritis (median of three question sets) | 100.0 |
| **S049** | GPT-4 | Correct match rate with US and Chinese osteoarthritis treatment guidelines | 46.4 |
| **S050** | ChatGPT-4o | Parameter-level agreement with physiotherapist consensus | 74.0 |
| **S072** | Gemini | Agreement with AAOS glenohumeral osteoarthritis recommendations | 75.0 |
| **S080** | ChatGPT-4o | Fully aligned responses, 2016 and 2018 EULAR gout recommendations | 76.0 |

**C2 Not eligible for a proportion-based summary (n = 2)**

| **Study** | **Reason for exclusion** |
| --- | --- |
| **S041** | Adherence to ACR recommendations reported qualitatively on a 5-point Likert scale ("mostly or fully adherent") |
| **S059** | Faithfulness to the retrieved guideline context reported only as a median score on a 5-point scale |

**D Direction of effect versus a human comparator (20 paired comparisons from 15 studies)**

| **Comparison family** | **Study** | **Direction of effect** | **Supporting values** |
| --- | --- | --- | --- |
| **Diagnostic accuracy versus clinicians** | S043 | Favours LLM | Claude 3.5 Sonnet 100% versus senior physicians 87% |
| **Diagnostic accuracy versus clinicians** | S026 | No clear difference | Top-1 35% versus 39% (p=0.3); top-3 60% versus 55% (p=0.38) |
| **Diagnostic accuracy versus clinicians** | S004 | Favours clinicians | Top-1 0% versus 35% (p=0.0083) |
| **Diagnostic accuracy versus clinicians** | S088 | Not directional | Concordance with the multidisciplinary team, kappa 0.49–0.64 |
| **Answer quality versus human-written or reference material** | S008 | Favours LLM | Quality 4.55 versus 4.31 (p=0.001); rater preference 57% versus 43% |
| **Answer quality versus human-written or reference material** | S020 | Favours LLM | Physician-rated accuracy 9.30 versus 5.38 (p<0.001) |
| **Answer quality versus human-written or reference material** | S016 | No clear difference | Accuracy and completeness scores identical (not significant) |
| **Answer quality versus human-written or reference material** | S028 | Mixed | Physician-rated comprehensiveness not significant; patient-rated 5.52 versus 8.76 (p<0.0001) |
| **Answer quality versus human-written or reference material** | S030 | Favours reference material | DISCERN 46 versus 52 (p<0.001) |
| **Answer quality versus human-written or reference material** | S005 | Favours clinicians | Rheumatology board plans preferred in 68.8% versus 16.3% of ratings |
| **Readability versus human-written or reference material** | S001 | Favours LLM | FKGL 8.29 versus 11.50 |
| **Readability versus human-written or reference material** | S013 | Favours LLM | FKGL 10.52 versus 15.06 (p<0.0001) |
| **Readability versus human-written or reference material** | S016 | Favours LLM | Gunning Fog Index 6.9 versus 8.8 (p<0.001) |
| **Readability versus human-written or reference material** | S023 | Favours LLM | FKGL 11.56 versus 12.67 (p<0.001) |
| **Readability versus human-written or reference material** | S020 | Favours human | Physician-rated readability 6.83 versus 8.33 (p<0.001) |
| **Readability versus human-written or reference material** | S028 | Favours human | Readability rating 7.85 versus 8.75 (p=0.0003) |
| **Readability versus human-written or reference material** | S030 | Favours reference material | FKGL 12.57 versus 11.14 (p=0.007); SMOG 14.30 versus 12.72 (p<0.001) |
| **Time or efficiency versus humans** | S023 | Favours LLM | 530 versus 37 words per minute (p<0.001) |
| **Time or efficiency versus humans** | S065 | Favours LLM | 42 seconds versus 8.4 minutes per assessment |
| **Time or efficiency versus humans** | S069 | Favours LLM | 5.67 versus 30.4 minutes per study extraction (p<0.00001) |

Comparisons were eligible when the study reported a numerical value for both the model output and a human comparator or reference material. Studies naming a human comparator without paired numerical values were not eligible.

Where a study evaluated both general-purpose and purpose-built or fine-tuned systems, the best-performing system as evaluated in that study was used (S089 SpAD-LLM-34B 92.4%; the best general-purpose model, GPT-4, reached 86.1%). S074 evaluated a multi-agent system and S083 a purpose-built custom model.

For S004, the value shown is the top-1 accuracy in deficiency of the interleukin-1 receptor antagonist; the corresponding familial Mediterranean fever estimate (50.0 %) is reported in section A1 and was the value used for the pooled median.

AAOS, American Academy of Orthopaedic Surgeons; ACR, American College of Rheumatology; axSpA, axial spondyloarthritis; EULAR, European Alliance of Associations for Rheumatology; FKGL, Flesch-Kincaid Grade Level; FMF, familial Mediterranean fever; LLM, large language model; SLE, systemic lupus erythematosus.

**Supplementary Table 3. Studies that appeared to meet the eligibility criteria but were excluded after full-text assessment**

*Reported in accordance with PRISMA 2020 item 16b.*

| **Study** | **Reason for exclusion** |
| --- | --- |
| La Bella S, Attanasi M, Porreca A, et al. Reliability of a generative artificial intelligence tool for pediatric familial Mediterranean fever: insights from a multicentre expert survey. Pediatr Rheumatol Online J. 2024;22(1):78. Published 2024 Aug 23. doi:10.1186/s12969-024-01011-0 | ineligible study design — expert-perception survey; reported outcomes are properties of the appraising experts (inter-rater agreement, trust) rather than of the model output. |
| Del Vescovo S, Vij P, Casteleyn C, et al. Clinical utility of large language models for osteoporosis referral triage: a pilot study. *RMD Open*. 2025;11(2):e005604. Published 2025 Jun 5. doi:10.1136/rmdopen-2025-005604 | Ineligible publication type — research letter; not entered into study-level extraction and risk-of-bias assessment. |
